# Green Light-Driven Hydroxylation of Boronic Acids Employing g-C_3_N_4_ as the Photocatalyst

**DOI:** 10.3390/molecules31081371

**Published:** 2026-04-21

**Authors:** Alexandros Emmanouil Troulos, Anastasia Maria Antonaki, Maria Zografaki, Vassilios Binas, Petros L. Gkizis

**Affiliations:** 1 Department of Chemistry, Aristotle University of Thessaloniki, 54124 Thessaloniki, Greece; atroulos@chem.auth.gr (A.E.T.); aantonaa@chem.auth.gr (A.M.A.); mzografaki@iesl.forth.gr (M.Z.); 2 Institute of Electronic Structure and Laser, Foundation for Research and Technology-Hellas (FORTH-IESL), 70013 Heraklion, Greece

**Keywords:** boronic acids, graphitic carbon nitride, hydroxylation, phenols, photochemistry

## Abstract

Phenol derivatives display a prominent role in many biologically active molecules. Boron-containing molecules are considered valuable precursors for their synthesis. Therefore, the rise of photochemistry has led many researchers to develop novel, sustainable protocols that exploit the advantages offered by different irradiation sources. For this reason, the application of novel photocatalysts that promote challenging organic transformations is highly valued. Graphitic carbon nitride (g-C_3_N_4_) is a semiconductor photocatalyst widely used in organic chemistry for promoting complex organic transformations. Herein, we report a green and efficient methodology for the hydroxylation of boronic acids to the corresponding hydroxyl derivatives, using g-C_3_N_4_ as the photocatalyst. The heterogeneous photocatalyst (g-C_3_N_4_) was prepared by thermal polycondensation of melamine and characterized by XRD, FESEM/EDS, and UV–Vis diffuse reflectance spectroscopy. Green LED irradiation was employed as the energy source and air as the active oxidant. A variety of substrates were tested, showcasing excellent functional group tolerance in the aerobic photochemical protocol. Mechanistic studies were conducted to investigate the reaction pathway and to identify the oxygen species generated.

## 1. Introduction

Phenols and alcohols are considered as valuable class of molecules and intermediates in Organic Synthesis. Accordingly, a plethora of synthetic approaches have been developed for their synthesis employing numerous compounds such as areres and halides [1,2]. Boronic acids and their ester derivatives are considered versatile substrates for the synthesis of hydroxy derivatives in high yields. Thus, boronic acids and/or esters attracted significant attention in recent years [3]. This specific transformation readily occurs due to the inherent stability of boronic acid derivatives and their compatibility with diverse reaction conditions. As a result, this reaction represents a key step in the synthesis of pharmaceuticals, agrochemicals, and materials science applications [4].

Over the past decades, the uprise of photochemistry has led many scientists to focus their research on developing sustainable and environmentally friendly methodologies for various challenging organic transformations [5,6]. The niche field of photochemistry has flourished over the last two decades, with a series of light-induced methodologies enabling organic transformations under milder and safer reaction conditions. This feature was quickly adopted by the pharmaceutical industry [7].

**Scheme 1:**
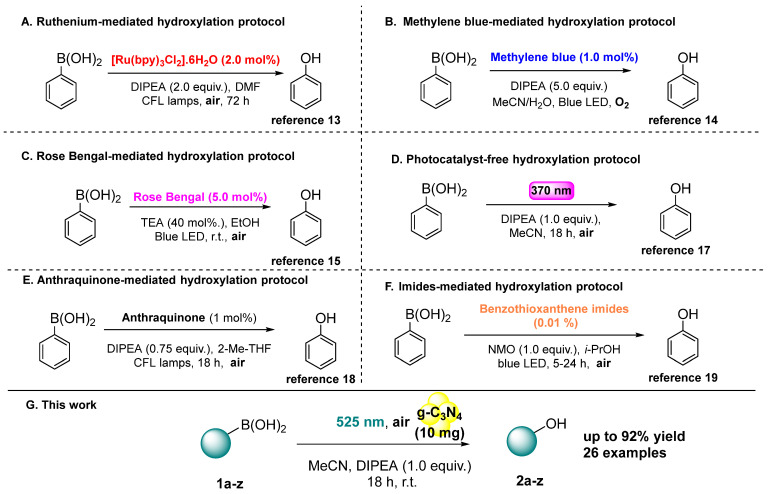
(**A**–**F**). Previous photochemical hydroxylation protocols; (**G**). this work.

The direct photochemical conversion of boronic acids and/or esters into the corresponding hydroxyl derivatives is a well-studied transformation [8] In order to avoid the use of metal-based catalysts [9,10] and/or stoichiometric amounts of oxidants [11,12], a plethora of photochemical protocols for the aerobic oxidation of boronic acids have been reported, utilizing various photocatalysts to promote the reaction. Jørgensen et al. exploited a ruthenium-based catalyst, performing the reaction in DMF (Scheme 1A) [13]. From a pharmaceutical industry perspective, the use of DMF, along with the prolonged reaction time, narrows its application in organic synthesis and raises major environmental concerns due to DMF’s toxicity. Later, Scaiano et al. investigated the use of an organic photocatalyst, such as methylene blue (Scheme 1B) [14]. Comparing the experimental data, the authors showcased the superiority of methylene blue over [Ru(bpy)_3_Cl_2_]·6H_2_O. Despite the excellent mechanistic approach, the photochemical protocol suffers from a limited substrate scope. In the same vein, Zhang et al. employed another organic dye, Rose Bengal, for the same conversion (Scheme 1C) [15]. This approach, however, suffers from high catalyst loading (5 mol%). Recently, the use of visible light and the application of low-energy irradiation sources, such as LED lamps or sunlight, for the promotion of chemical reactions have been recognized as a major research focus due to their potential to create green and low-energy protocols [16]. From 2023 onward, Kokotos et al., taking advantage of wavelength-dependent irradiation effects, reported a series of elegant photochemical protocols for the aerobic hydroxylation of boronic acid derivatives. More specifically, they reported a UVA-promoted methodology [17] and two low-catalyst-loading methodologies utilizing anthraquinone and benzothioxanthene imides as photocatalysts, with CFL lamps or blue LEDs as the irradiation source (Scheme 1D–F) [18,19]. In these elegant synthetic approaches, either harsh photochemical reaction conditions or photocatalysts that cannot be easily reused are required. In an alternative catalyst-free strategy, Zhang et al. reported an aerobic hydroxylation protocol that proceeds without the use of either a photocatalyst or any additives [20]. In this study, blue LED irradiation at 420 nm was employed, with THF serving as the reaction solvent. However, a significant limitation of this method is the use of THF, which can form peroxides in the presence of oxygen, raising important safety concerns for industrial applications.

Graphitic carbon nitride (g-C_3_N_4_) is a well-known polymeric photocatalyst used in photochemical organic transformations [21]. Recently, it has attracted great attention as a visible light, metal-free, and low-cost photocatalyst, adding an alternative option to the researcher’s arsenal. g-C_3_N_4_ is considered a versatile and eco-friendly photocatalyst with a remarkable ability to address pressing environmental and energy challenges [22,23]. Its wide range of applications spans various domains, each contributing to the advancement of sustainability. At the molecular level, g-C_3_N_4_ consists of heptazine-based units arranged in a layered, π-conjugated polymeric framework, which endows the material with unique electronic and photophysical properties. Owing to its moderate band gap and heterogeneous nature, g-C_3_N_4_ can efficiently mediate photoinduced energy- and electron-transfer processes while being easily recovered and reused. Importantly, g-C_3_N_4_ has been widely reported to promote the generation of reactive oxygen species (ROS) under visible-light irradiation, making it particularly attractive for sustainable aerobic oxidation reactions. These characteristics make g-C_3_N_4_ an ideal platform for the development of green, low-energy photochemical transformations.

In our group, over the past years, we have focused our research on developing a series of eco-friendly, sustainable protocols [24]. Within this context, we examined the impact of the irradiation wavelength on the aerobic hydroxylation of boronic acids to promote the transformation under milder reaction conditions. In 2020, Yu et al. reported the use of copper-doped g-C_3_N_4_ for the oxidative hydroxylation of aryl boronic acids [25]. In this approach, overstoichiometric amounts of DIPEA were employed. Additionally, the reaction was carried out under blue-light irradiation (460 nm), under which it is known to proceed efficiently even in the absence of a photocatalytic medium [17]. In a related approach, Wahid et al. developed a heterogeneous photoredox catalyst comprising a MOF-derived nickel phosphide integrated with barbituric acid-modified graphitic carbon nitride for the aerobic hydroxylation of boronic acid derivatives [26]. Despite the elegance of this system, it relies on relatively harsh reaction conditions, including high-intensity xenon lamp irradiation (125 W). Moreover, the use of metal-based photocatalysts is generally viewed unfavorably in the pharmaceutical industry.

With the excellent photocatalytic activity of g-C_3_N_4,_ we envisioned its application toward the hydroxylation of boronic acid derivatives. Herein, we report a novel, sustainable aerobic photochemical protocol for the hydroxylation reaction of boronic acids into the corresponding hydroxyl derivatives. In collaboration with Binas’ research group, we explored the use of graphitic carbon nitride (g-C_3_N_4_) as the photocatalyst to generate oxygen species and promote this oxidative transformation. Having examined the impact of irradiation wavelength on the aerobic hydroxylation process of boronic acid derivatives, we sought to develop a photochemical protocol under even milder reaction conditions. Thus, we report a green-light-driven, sustainable, aerobic hydroxylation protocol for the photochemical conversion of boronic acids into their hydroxyl derivatives.

## 2. Results and Discussion

At the outset, we examined the impact of irradiation wavelength on the hydroxylation reaction, utilizing phenyl boronic acid (**1a**) as the model substrate (Supplementary Materials). To this end, we conducted the reaction under various LED wavelengths (Supplementary Materials). We observed that use of LED lamps as the irradiation source promoted the reaction, leading to the desired product. It was found that decreasing the wavelength of the LED irradiation increased the conversion. On the other hand, under green-light irradiation, the reaction did not take place (Supplementary Materials). With the goal of developing a photochemical protocol under mild reaction conditions, we continued our efforts using green LED lamps as the irradiation source. Since the hydroxylation reaction does not proceed in the absence of a photocatalyst, we examined the use of graphitic carbon nitride (g-C_3_N_4_) in this role. The g-C_3_N_4_ photocatalyst employed herein was prepared by thermal polycondensation of melamine and characterized by XRD, FESEM/EDS, and UV–Vis diffuse reflectance spectroscopy (Supplementary Materials). The diffraction pattern and morphology are consistent with layered polymeric carbon nitride, while EDS confirms the presence of C and N as the dominant elements. The optical response (Tauc analysis) indicates a band gap of ca. 2.74 eV, supporting photoactivation under the applied low-energy irradiation conditions, likely via absorption tail or defect-related states (Supplementary Materials). We performed the reaction utilizing phenyl boronic acid (**1a**), g-C_3_N_4_ (6 mg), and *N*,*N*′-diisopropylethylamine (DIPEA) in acetonitrile under green-light irradiation for 18 h. The reaction proceeded, affording the desired phenol (**2a**) in 58% yield.

Next, we turned our attention to identifying the optimum reaction medium (Table 1) (Supplementary Materials). Conducting the reaction in polar solvents such as dichloromethane, chloroform, 1,2-dichloroethane, or ethyl acetate led to moderate reaction yields (Table 1, entries 2–5). The use of polar protic solvents such as methanol or isopropanol promoted the reaction poorly (Table 1, entries 6–7). On the other hand, the use of ethereal solvents, dimethylsulfoxide (DMSO), or acetone proved to be a poor choice, as the reaction did not take place (Table 1, entries 8–11). In non-polar solvents (petroleum ether) and aromatic solvents such as benzene and toluene, the desired product was formed in moderate reaction yields (Table 1, entries 12–14). With a greener solvent in mind, we conducted the reaction in water, which failed to form the desired product (Table 1, entry 15). The best result was obtained when acetonitrile was employed as the reaction medium (Table 1, entry 1).

Continuing our efforts, we studied the impact of the base on the reaction outcome (Table 2). Having already employed DIPEA as the base, we examined its use at lower stoichiometric ratios (Supplementary Materials). In this case, we observed that the reaction yield decreased (Table 2, entries 1-3). Surprisingly, use of other amines did not promote the reaction (Table 2, entries 4–9). Primary amines, such as butylamine, or secondary amines, such as morpholine, pyrrolidine, or piperidine did not lead to the formation of phenol (**2a**) (Table 2, entries 4–7). In the same vein, tertiary amines such as triethylamine or *N*,*N*,*N*′,*N*′-tetramethylenediamine proved to be poor choices (Table 2, entries 8–9). Additional experiments to determine the optimum reaction concentration resulted in the use of 4 mL/mmol acetonitrile (Supplementary Materials). 

To increase the reaction yield of the hydroxylation protocol, we also examined the catalyst loading. Increasing the quantity of g-C_3_N_4_ from 6 mg to 10 mg led to full consumption of phenyl boronic acid (**1a**) and formation of phenol (**2a**) in 92% yield (Supplementary Materials). Control experiments confirmed the crucial role of the reaction parameters (Supplementary Materials). Indeed, in the absence of any of these parameters, the photochemical hydroxylation of phenyl boronic acid (**1a**) does not take place (Supplementary Materials). 

**Scheme 2:**
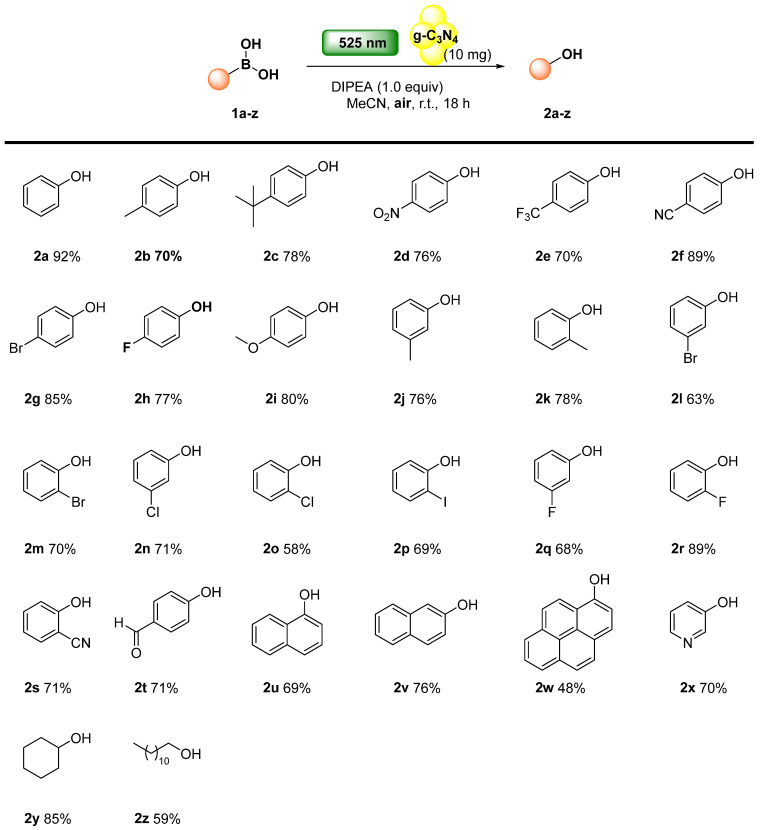
Substrate scope for the photochemical hydroxylation of boronic acid derivatives.

Having in hand the optimal reaction conditions, we turned our interest to the substrate scope to examine the functional group tolerance of the photochemical hydroxylation protocol. For this reason, a wide variety of boronic acid derivatives were used under the optimal reaction conditions (Scheme 2). Initially, a variety of *p*-substituted arylboronic acids were tested, affording the corresponding phenol derivatives in good to excellent yields (Scheme 2, **2a**–**i** and **2t**). Alkyl substituents attached to the phenyl moiety afforded the corresponding phenols in excellent yields (Scheme 2, **2b**–**c**). The presence of an electron-withdrawing group attached to the benzene ring led to the corresponding phenols in good to excellent yields (Scheme 2, **2d**–**h** and **2t**). Aromatic boronic acid derivatives decorated with electron-donating groups, such as a methoxy group, afforded the desired product in good yield (80%) (Scheme 2, **2i**). It must be mentioned that, in some cases, despite the full consumption of the starting material, lower yields were obtained (Scheme 2, **2o**–**q**), which can be attributed to the possible sublimation of the formed product.

Next, we examined the impact of the substituent position on the reaction yield. In all cases, it was found that the position of the substituent on the phenyl moiety did not affect the reaction output (Scheme 2, **2j**–**s**). Regarding the functional group tolerance, alkyl, nitro, trifluoromethyl, cyano, halogen, and formyl groups attached to the benzene ring remained intact under the reaction conditions (Scheme 2, **2a**–**t**). Furthermore, fused aromatic boronic acid derivatives were tested, affording the corresponding phenol derivatives in good to excellent yields (Scheme 2, **2u**–**w**). Finally, when pyridine boronic acid (**1u**) was employed, the corresponding 3-hydroxy pyridine (**2x**) was formed as the sole product, without observing the formation of an *N*-oxidized byproduct derived from oxidation of the sensitive pyridine moiety. Seeking additional candidates for our photochemical protocol, we also examined the use of alkyl boronic acid derivatives, employing cyclohexyl (**1y**) and dodecyl boronic acid (**1z**) derivatives. Under the reaction conditions, the corresponding alcohols (**2y**–**z**) were formed in excellent yields (Scheme 2).

**Scheme 3:**
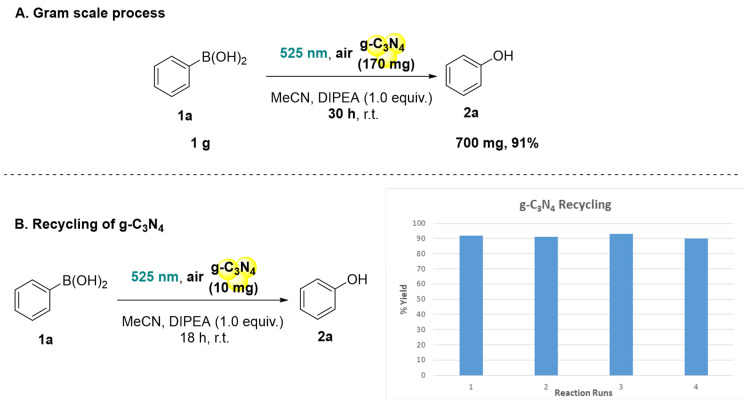
(**A**). Gram-scale reaction for the photochemical hydroxylation of phenyl boronic acid (**1a**); (**B**). Recycling of g-C_3_N_4_ in the photochemical hydroxylation of phenyl boronic acid (**1a**).

To further showcase the industrial feasibility and scalability of our photochemical aerobic protocol, we performed the hydroxylation of phenyl boronic acid (**1a**) on a gram scale, leading to phenol (**2a**) in excellent yield (**91%**) after 30 h of irradiation (Scheme 3A). Since the industrial applicability of a reaction protocol is directly linked to catalyst reuse, we also examined the recyclability of g-C_3_N_4_. The photochemical hydroxylation of phenyl boronic acid (**1a**) proceeded over four consecutive cycles without significant loss in the reaction yield (Scheme 3B).

## 3. Mechanistic Studies

**Scheme 4:**
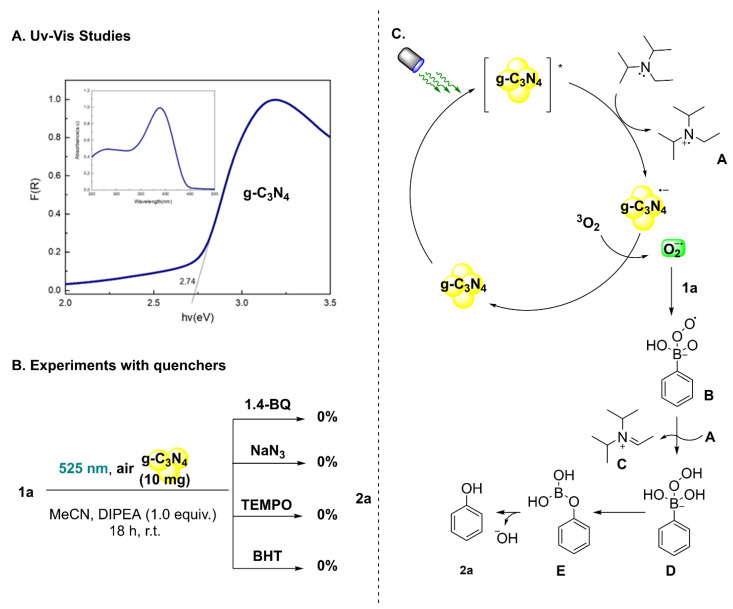
(**A**). UV–Vis spectrum of catalyst; (**B**). Quench studies; (**C**). Mechanistic scenario.

The most challenging aspect of every novel photochemical protocol appears to be the identification of the oxygen species generated under these specific photochemical conditions. Initially, a series of UV–Vis studies was conducted to investigate the possible formation of an EDA complex [27]. The crucial role of EDA complexes in photochemical reaction protocols is well reported in the literature [27]. In our trials, we did not observe any interaction between g-C_3_N_4_, phenyl boronic acid (**1a**), and/or DIPEA, likely due to the low dispersion of g-C_3_N_4_. Therefore, the formation of an EDA complex remains inconclusive. On the other hand, despite the low dispersion of g-C_3_N_4_ in methanol, low absorption in the 400–500 nm range was observed (Scheme 4A) (Supplementary Materials). Steady-state PL spectra of pristine g C_3_N_4_ (Supplementary Materials, Figure S7a) show a strong emission peak at 440–450 nm, together with a distinct low-energy tail extending beyond 550 nm. This spectral feature is consistent with the presence of defect- or surface-related emissive states and indicates the existence of mid-gap or sub-bandgap electronic levels. In combination with the diffuse reflectance data and the observed photocatalytic activity under 525 nm irradiation, these results support the proposed defect-assisted sub-bandgap activation pathway of g-C_3_N_4_ under green light (Supplementary Materials). 

To further identify the generated active oxygen species, we conducted the photochemical hydroxylation of phenyl boronic acid (**1a**) in the presence of several scavengers, such as sodium azide (NaN_3_) and 1,4-benzoquinone (1,4-BQ) (Scheme 4B). When NaN_3_ was employed, the reaction yield was 0%. Since NaN_3_ is considered a singlet oxygen scavenger, this result supports the generation of singlet oxygen triggered by an energy transfer event from the excited state of g-C_3_N_4_. A similar result was obtained when 1,4-benzoquinone (1,4-BQ), a known superoxide anion scavenger, was employed. This supports the mechanistic scenario in which singlet oxygen interacts with DIPEA and, via a SET event, is converted to a superoxide anion that promotes the reaction (Scheme 4C). Furthermore, in the presence of TEMPO and BHT, the reaction did not proceed, revealing the radical character of our photochemical protocol (Scheme 4B).

We also performed control experiments to detect the possible formation of H_2_O_2_, which is known to promote this oxidative transformation. Additionally, it is well established in the literature that the formation of H_2_O_2_ promotes the reaction. In our case, all control experiments showed that H_2_O_2_ is not generated (Supplementary Materials). 

Additionally, the quantum yield of the photochemical reaction protocol was determined based on the protocol reported by Yoon et al [28]. The value of *Φ* = 0.08 supports a closed catalytic cycle.

Taking into account our findings, a plausible mechanistic pathway is depicted in Scheme 4C. Upon green-light irradiation (525 nm), g-C_3_N_4_ is photoactivated, most likely through sub-bandgap absorption features (e.g., absorption tail and defect/surface states), enabling energy-transfer processes with molecular oxygen. In line with its metal-free nature, FESEM/EDS analysis confirms that carbon and nitrogen are the predominant elements, with no detectable metal impurities that could otherwise complicate the mechanistic interpretation. The excited state photocatalyst undergoes an SET event, generating intermediate **A** from DIPEA. It is well established in the literature that the generated radical anion interacts with ground state oxygen through a SET event to the form superoxide anion [14]. The latter interacts with phenyl boronic acid (**1a**), forming intermediate **B**. A HAT event mediated by DIPEA radical cation **A** and intermediate **B** leads to the formation of intermediate **D**. Finally, the desired product (**2a**) is formed through a rearrangement step from **D**, followed by hydrolysis.

## 4. Materials and Methods

### 4.1. General Remarks

All commercially available products and solvents were purchased from Fluorochem (Fluorochem Ltd., Hadfield, Glossop, UK), Sigma-Aldrich (Sigma-Aldrich, Saint Louis, MO, USA), Fluka (Fluka Chemicals Ltd., Gillingham, Dorset, UK), Merck (Merck, Darmstadt, Germany), and Alfa Aesar (Alfa Aesar, Ward Hill, MA, USA). Chromatographic purification of products was accomplished using forced-flow chromatography on Merck^®^ (Merck, Darmstadt, Germany) Kieselgel 60 F254 (230–400 mesh). Thin-layer chromatography (TLC) was performed on aluminum-backed silica plates (0.2 mm, 60 F254). Visualization of the developed chromatogram was performed by fluorescence quenching using phosphomolybdic acid. Melting points were measured on a Büchi 530 apparatus (Büchi, Flawil, Switzerland) and are uncorrected. High-resolution mass spectra were obtained on a Bruker Maxis Impact QTOF spectrometer (Bruker Daltonics, Bremen, Germany). ^1^H-NMR, ^13^C-NMR, and ^19^F-NMR spectra were recorded on an Ascend^TM^ Bruker (300 MHz (300 MHz, 75 MHz, and 282 MHz, respectively) or an Agilent Technologies DD2 (500 MHz, 125 MHz, and 470 MHz, respectively) and are internally referenced to residual solvent signals. Data for ^1^H-NMR are reported as follows: chemical shift (*δ* ppm), multiplicity (s = singlet, t = triplet, quin = quintet, m = multiplet, br s = broad signal), coupling constant, integration, and assignment. Data for ^13^C-NMR are reported in terms of chemical shift (*δ* ppm).

### 4.2. Preparation of g-C_3_N_4_

Graphitic carbon nitride (g-C_3_N_4_) was synthesized via thermal polycondensation of melamine. In a typical experiment, melamine (50 g) was placed in a covered ceramic crucible and heated in a muffle furnace under airflow at a heating rate of 2 °C/min. The temperature was maintained at 510 °C for 2 h and subsequently at 530 °C for an additional 2 h. After natural cooling to room temperature, the obtained yellow solid was ground into a fine powder and used as received. Calcination of 50 g melamine typically afforded ~30 g-C_3_N_4_ (ca. 60% yield).

### 4.3. Characterization of g-C_3_N_4_

X-ray diffraction (XRD) was used to confirm the formation of polymeric carbon nitride. The morphology was examined by field-emission scanning electron microscopy (FESEM), while elemental composition was assessed by energy-dispersive X-ray spectroscopy (EDS). The optical properties were evaluated by UV–Vis diffuse reflectance spectroscopy (DRS), and the band gap was estimated using Tauc analysis. Further details and representative spectra/micrographs are provided in the Supplementary Materials.

### 4.4. General Procedure for the Photochemical Hydroxylation Reaction of Boronic Acid Derivatives

In a glass vial containing the corresponding boronic acid (0.50 mmol) and *N*,*N*′-diisopropylethylamine (65 mg, 0.50 mmol), g-C_3_N_4_ (10 mg) in acetonitrile (2.0 mL) were added. The reaction mixture was stirred vigorously under irradiation from LED lamps (Kessil PR 160L, 525 nm) for 18 h. Alcohols **2a**–**z** were purified by flash chromatography on silica gel, eluting with a petroleum ether (bp 40–60 °C)/ethyl acetate (60:40) system.

*Phenol* (**2a**) Yield: 92%; White solid; m.p.: 41–42 °C; ^1^H-NMR (300 MHz, CDCl_3_) δ: 7.25 (2H, t, *J* = 7.5 Hz, ArH), 6.94 (1H, t, *J* = 7.5 Hz, ArH), 6.85 (2H, d, *J* = 7.5 Hz, ArH), 4.28 (1H, br s, OH); ^13^C-NMR (75 MHz, CDCl_3_) δ: 155.5, 129.7, 120.8, 115.3; MS (ESI) *m*/*z* 93 [M − H]^−^.

*p-Cresol* (**2b**)Yield: 70%; White solid; m.p.: 33–35 °C; ^1^H-NMR (500 MHz, CDCl_3_) δ: 7.06 (2H, d, *J* = 8.2 Hz, ArH), 6.78 (2H, d, *J* = 8.2 Hz, ArH), 5.44 (1H, br s, OH), 2.30 (1H, s, CH_3_); ^13^C-NMR (125 MHz, CDCl_3_) δ: 153.6, 130.4, 130.3, 115.4, 20.8; MS (ESI) *m*/*z* 107 [M − H]^−^.

*4-(tert-Butyl)phenol* (**2c**) Yield: 78%; White solid; m.p.: 90–92 °C; ^1^H-NMR (300 MHz, CDCl_3_) δ: 7.28 (2H, d, *J* = 7.0 Hz, ArH), 6.80 (2H, d, *J* = 7.0 Hz, ArH), 4.95 (1H, br s, OH), 1.32 (9H, s, CH_3_); ^13^C-NMR (75 MHz, CDCl_3_) δ: 153.0, 143.6, 126.5, 114.8, 34.1, 31.5; MS (ESI) *m*/*z* 150 [M]+.

*4-Nitrophenol* (**2d**) Yield: 76%; Yellow solid; m.p.: 110–112 °C; ^1^H-NMR (300 MHz, CDCl_3_) δ: 8.18 (2H, d, *J* = 9.1 Hz, ArH), 6.93 (2H, d, *J* = 9.1 Hz, ArH), 5.78 (1H, br s, OH); ^13^C-NMR (75 MHz, CDCl_3_) δ: 116.7, 142.0, 126.6, 116.0; MS (ESI) *m*/*z* 140 [M + H]^+^.

*4-(Trifluoromethyl)phenol* (**2e**) Yield: 60%; White solid; m.p.: 40–42 °C; ^1^H-NMR (500 MHz, CDCl_3_) δ: 7.51 (2H, d, *J* = 8.4 Hz, ArH), 6.90 (2H, d, *J* = 8.4 Hz, ArH), 5.38 (1H, br s, OH); ^13^C-NMR (125 MHz, CDCl_3_) δ: 158.6, 127.3 (q, *J* = 3.8 Hz), 124.5 (q, *J* = 271.0 Hz), 123.2 (q, *J* = 32.7 Hz), 115.8; ^19^F-NMR (470 MHz, CDCl_3_) δ: -61.5; MS (ESI) *m*/*z* 161 [M − H]^−^.

*4-Hydroxybenzonitrile* (**2f**) Yield: 89%; Brown solid; m.p.: 98–100 °C; ^1^H-NMR (500 MHz, CDCl_3_) δ: 7.54 (2H, d, *J* = 8.9 Hz, ArH), 6.94 (2H, d, *J* = 8.9 Hz, ArH); ^13^C-NMR (125 MHz, CDCl_3_) δ: 160.7, 134.7, 119.6, 116.8, 103.3; MS (ESI) *m*/*z* 120 [M + H]^+^.

*4-Bromophenol* (**2g**) Yield: 85%; Red solid; m.p.: 66–68 °C; ^1^H-NMR (300 MHz, CDCl_3_) δ: 7.34 (2H, d, *J* = 8.9 Hz, ArH), 6.73 (2H, d, *J* = 8.9 Hz, ArH); ^13^C-NMR (75 MHz, CDCl_3_) δ: 154.9, 132.9, 117.6, 113.4; MS (ESI) *m*/*z* 172/174 [M]^+^.

*4-Fluorophenol* (**2h**) Yield: 77%; Red solid; m.p.: 41–43 °C; ^1^H-NMR (500 MHz, CDCl_3_) δ: 6.91 (2H, t, *J* = 8.5 Hz, ArH), 6.78 (2H, dd, *J* = 8.5 and 4.2 Hz, ArH), 4.14 (1H, br s, OH); ^13^C-NMR (125 MHz, CDCl_3_) δ: 157.3 (d, *J* = 237.9 Hz), 151.4 (d, *J* = 2.1 Hz), 116.3 (d, *J* = 8.0 Hz), 116.0 (d, *J* = 23.2 Hz); ^19^F-NMR (470 MHz, CDCl_3_) δ: -124.2; MS (ESI) *m*/*z* 111 [M − H]^−^.

*4-Methoxyphenol* (**2i**) Yield: 80%; White solid; m.p.: 55–58 °C; ^1^H-NMR (300 MHz, CDCl_3_) δ: 6.84 (4H, m, ArH), 3.76 (3H, s, OCH_3_); ^13^C-NMR (75 MHz, CDCl_3_) δ: 153.7, 148.4, 116.2, 115.0, 56.0; MS (ESI) *m*/*z* 123 [M − H]^−^.

*m-Cresol* (**2j**) Yield: 76%; Colorless oil; ^1^H-NMR (500 MHz, CDCl_3_) δ: 7.14 (1H, t, *J* = 7.7 Hz, ArH), 6.78 (1H, d, *J* = 7.7 Hz, ArH), 6.70–6.64 (2H, d, *J* = 7.7 Hz ArH), 5.31 (1H, br s, OH), 2.32 (3H, s, CH_3_); ^13^C-NMR (125 MHz, CDCl_3_) δ: 155.7, 140.2, 129.8, 122.0, 116.4, 112.7, 21.6; MS (ESI) *m*/*z* 107 [M − H]^−^.

*o-Cresol* (**2k**)Yield: 78%; Colorless oil; ^1^H-NMR (500 MHz, CDCl_3_) δ: 7.13 (1H, d, *J* = 7.5 Hz, ArH), 7.09 (1H, d, *J* = 7.5 Hz, ArH), 6.85 (1H, t, *J* = 7.5 Hz, ArH), 6.78 (1H, d, *J* = 7.5 Hz, ArH), 4.70 (1H, br s, OH), 2.26 (3H, s, CH_3_); ^13^C-NMR (125 MHz, CDCl_3_) δ: 153.8, 131.1, 127.2, 123.7, 120.8, 114.9, 15.7; MS (ESI) *m*/*z* 107 [M − H]^−^.

*3-Bromophenol* (**2l**) Yield: 63%; Colorless oil; ^1^H-NMR (500 MHz, CDCl_3_) δ: 7.12–7.06 (2H, m, ArH), 7.03-7.02 (1H, m, ArH), 6.79–6.76 (1H, m, ArH), 4.69 (1H, br, OH); ^13^C-NMR (125 MHz, CDCl_3_) δ: 156.6, 131.2, 124.4, 123.1, 119.2, 114.6; MS (ESI) *m*/*z* 172/174 [M]+.

*2-Bromophenol* (**2m**) Yield: 70%; Yellow oil; ^1^H-NMR (500 MHz, CDCl_3_) δ: 7.47 (1H, d, *J* = 8.1 Hz, ArH), 7.22 (1H, t, *J* = 8.1 Hz, ArH), 7.03 (1H, d, *J* = 8.1 Hz, ArH), 6.81 (1H, t, *J* = 8.1 Hz, ArH), 5.46 (1H, br s, OH); ^13^C-NMR (125 MHz, CDCl_3_) δ: 152.6, 132.4, 129.5, 122.2, 116.5, 110.6; MS (ESI) *m*/*z* 172/174 [M]^+^.

*3-Chlorophenol* (**2n**) Yield: 71%; White solid; m.p.: 30–33 °C; ^1^H-NMR (500 MHz, CDCl_3_) δ: 7.16 (1H, t, *J* = 8.1 Hz, ArH), 6.92 (1H, d, *J* = 8.1 Hz, ArH), 6.86 (1H, s, ArH), 6.72 (1H, d, *J* = 8.1, ArH), 5.07 (1H, br s, OH); ^13^C-NMR (125 MHz, CDCl_3_) δ: 156.6, 135.3, 130.8, 121.5, 116.3, 114.1; MS (ESI) *m*/*z* 127/129 [M − H]^−^.

*2-Chlorophenol* (**2n**) Yield: 58%; Colorless oil; ^1^H-NMR (500 MHz, CDCl_3_) δ: 7.29 (1H, d, *J* = 8.2 Hz, ArH), 7.20–7.16 (1H, m, ArH), 7.00 (1H, d, *J* = 8.2 Hz, ArH), 6.85 (1H, t, *J* = 8.2 Hz, ArH), 5.55 (1H, br s, OH); ^13^C-NMR (125 MHz, CDCl_3_) δ: 151.6, 129.3, 128.7, 121.6, 120.2, 116.5; MS (ESI) *m*/*z* 127/129 [M − H]^−^.

*2-Iodophenol* (**2p**) Yield: 69%; Yellow oil; ^1^H-NMR (500 MHz, CDCl_3_) δ: 7.65 (1H, dd, *J* = 7.9 and 1.5 Hz, ArH), 7.26–7.22 (1H, m, ArH), 7.00 (1H, dd, *J* = 7.9 and 1.5 Hz, ArH), 6.69-6.65 (1H, m, ArH), 5.28 (1H, br s, OH); ^13^C-NMR (125 MHz, CDCl_3_) δ: 155.2, 138.6, 130.6, 122.8, 115.5, 86.1; MS (ESI) *m*/*z* 221 [M + H]^+^.

*3-Fluorophenol* (**2q**)Yield: 68%; Orange solid; m.p.: 150–152 °C ^1^H-NMR (500 MHz, CDCl_3_) δ: 7.21–7.15 (1H, m, ArH), 6.66-6.56 (3H, m, ArH), 4.87 (1H, br s, OH); ^13^C-NMR (125 MHz, CDCl_3_) δ: 163.8 (d, *J* = 245.5 Hz), 159.6 (d, *J* = 11.3 Hz), 130.6 (d, *J* = 10.1 Hz), 111.2 (d, *J* = 2.9 Hz), 107.9 (d, *J* = 21.2 Hz), 103.4 (d, *J* = 24.5 Hz); ^19^F-NMR (470 MHz, CDCl_3_) δ: -111.8; MS (ESI) *m*/*z* 111 [M − H]^−^.

*2-Fluorophenol* (**2r**)Yield: 89%; Colorless oil; ^1^H-NMR (500 MHz, CDCl_3_) δ: 7.10–6.98 (3H, m, ArH), 6.88-6.82 (1H, m, ArH), 5.10 (1H, br s, OH); ^13^C-NMR (125 MHz, CDCl_3_) δ: 151.2 (d, *J* = 237.3 Hz), 143.9 (d, *J* = 14.2 Hz), 125.1 (d, *J* = 3.8 Hz), 121.1 (d, *J* = 6.5 Hz), 117.6, 115.8 (d, *J* = 18.0 Hz); ^19^F-NMR (470 MHz, CDCl_3_) δ: -141.4; MS (ESI) *m*/*z* 111 [M − H]^−^.

*2-Hydroxybenzonitrile* (**2s**) Yield: 71%; Yellow solid; m.p.: 88–90 °C; ^1^H-NMR (500 MHz, CDCl_3_) δ: 7.51–7.44 (2H, m, ArH), 7.02 (2H, d, *J* = 8.4 Hz, ArH), 6.82 (1H, br s, OH); ^13^C-NMR (125 MHz, CDCl_3_) δ: 159.0, 135.1, 133.3, 121.3, 117.0, 116.8, 99.8; MS (ESI) *m*/*z* 120 [M + H]^+^.

*4-Formyl-phenol* (**2t**) Yield: 71%; brown solid; m.p.: 114–116 °C; ^1^H-NMR (300 MHz, CDCl_3_) δ: 9.85 (1H, s, CHO), 7.82 (2H, d, *J* = 8.5 Hz, ArH), 6.98 (2H, d, *J* = 8.5 Hz, ArH), 6.56 (1H, br s, OH); ^13^C-NMR (75 MHz, CDCl_3_) δ: 191.3, 161.6, 132.6, 129.9, 116.0; MS (ESI) *m*/*z* 121 [M − H]^−^.

*Naphthalen-1-ol* (**2u**) Yield: 69%; White solid; m.p.: 118–120 °C; ^1^H-NMR (500 MHz, CDCl_3_) δ: 8.20–8.18 (1H, m, ArH), 7.84-7.81 (1H, m, ArH), 7.51-7.49 (2H, m, ArH), 7.45 (1H, d, *J* = 7.8 Hz, ArH), 7.31 (1H, t, *J* = 7.8 Hz, ArH), 6.82 (1H, d, *J* = 7.8 Hz, ArH), 5.30 (1H, br s, OH); ^13^C-NMR (125 MHz, CDCl_3_) δ: 151.7, 135.1, 128.0, 126.8, 126.2, 125.6, 124.7, 121.9, 121.1, 109.0; MS (ESI) *m*/*z* 143 [M − H]^−^.

*Naphthalen-2-ol* (**2v**)Yield: 76%; White solid; m.p.: 90–92 °C; ^1^H-NMR (500 MHz, CDCl_3_) δ: 7.77 (2H, t, *J* = 8.5 Hz, ArH), 7.68 (1H, d, *J* = 8.5 Hz, ArH), 7.44 (1H, t, *J* = 8.5 Hz, ArH), 7.34 (1H, t, *J* = 8.5 Hz, ArH), 7.13 (2H, t, *J* = 8.5 Hz, ArH), 4.49 (1H, br s, OH); ^13^C-NMR (125 MHz, CDCl_3_) δ: 153.7, 134.9, 130.2, 129.3, 128.1, 126.7, 126.7, 124.0, 118.1, 109.9; MS (ESI) *m*/*z* 143 [M − H]^−^.

*Pyren-1-ol* (**2w**) Yield: 48%; Brown solid; m.p.: 168–170 °C; ^1^H-NMR (500 MHz, CDCl_3_) δ: 8.34 (1H, d, *J* = 8.9 Hz, ArH), 8.11 (2H, d, *J* = 7.3 Hz, ArH), 8.04 (2H, t, *J* = 8.3 Hz, ArH), 7.97 (2H, dd, *J* = 8.3 and 7.3 Hz, ArH), 7.90 (1H, d, *J* = 8.9 Hz, ArH), 7.47 (1H, d, *J* = 8.3 Hz, ArH), 5.57 (1H, br s, OH); ^13^C-NMR (125 MHz, CDCl_3_) δ: 149.7, 131.8, 131.7, 127.3, 126.6, 126.2, 126.1, 125.7, 125.5, 125.0, 124.5, 124.3, 120.5, 118.7, 113.1; MS (ESI) *m*/*z* 219 [M + H]^+^.

*Pyridin-3-ol* (**2x**) Yield: 67%; White solid; m.p.: 119–121 °C; ^1^H-NMR (500 MHz, CDCl_3_) δ: 8.32 (1H, d, *J* = 2.8 Hz, ArH), 8.09 (1H, dd, *J* = 4.7 and 1.3 Hz, ArH), 7.36 (1H, ddd, *J* = 8.4, 2.8 and 1.4 Hz, ArH), 7.29 (1H, dd, *J* = 8.4 and 4.7 Hz, ArH), 5.76 (1H, br s, OH); ^13^C-NMR (125 MHz, CDCl_3_) δ: 155.3, 138.6, 136.0, 125.6, 125.3; MS (ESI) *m*/*z* 96 [M + H]+.

*Cyclohexanol* (**2y**) Yield: 85%; Colorless oil; ^1^H-NMR (300 MHz, CDCl_3_) δ: 3.61–3.58 (1H, m, CHOH), 1.91–1.86 (2H, m, 2 x CHH), 1.76–1.70 (2H, m, CHH and OH), 1.56–1.51 (1H, m, CHH), 1.30–1.23 (6H, m, 6 x CHH); ^13^C-NMR (75 MHz, CDCl_3_) δ: 69.9, 35.3, 25.3, 24.0; MS (ESI) *m*/*z* 101 [M + H]^+^.

*1-Dodecanol* (**2z**) Yield: 59%; Colorless oil; ^1^H-NMR (300 MHz, CDCl_3_) δ: 3.63 (2H, t, *J* = 6.4 Hz, CH_2_), 1.58–1.56 (2H, m, CH_2_), 1.32–1.21 (18H, m, 9 x CH_2_), 0.88 (3H, t, *J* = 6.4 Hz, CH_3_); ^13^C-NMR (75 MHz, CDCl_3_) δ: 63.1, 32.8, 31.9, 29.6, 29.6, 29.6, 29.6, 29.4, 29.3, 25.7, 22.7, 14.1; MS (ESI) *m*/*z* 186 [M]^+^.

## 5. Conclusions

The rise of photochemistry has opened new opportunities by enabling access to previously unexplored reactivities. Researchers in both academia and industry have incorporated photochemical strategies as an additional tool in the design of novel synthetic routes. Over the years, oxygen-mediated photochemical hydroxylation of boronic acids has attracted considerable attention. Herein, we report a new photochemical protocol for the hydroxylation of boronic acid derivatives to their corresponding phenols using graphitic carbon nitride (g-C_3_N_4_) as a photocatalyst. g-C_3_N_4_ proved to be an excellent mediator for boronic acid photochemical hydroxylation under mild reaction conditions. Moreover, the use of a readily prepared, recyclable, metal-free g-C_3_N_4_ photocatalyst enables efficient aerobic hydroxylation under low-energy green-light irradiation, providing a practical and sustainable alternative to homogeneous photocatalytic systems. The ability of g-C_3_N_4_ to generate singlet oxygen, along with its conversion to superoxide anion in the presence of DIPEA, is effectively exploited in this aerobic photochemical process. A wide range of substituted boronic acid derivatives was successfully employed, demonstrating the broad functional group tolerance of the methodology. Furthermore, extensive mechanistic investigations were conducted to identify the reactive species involved in the transformation.

## Figures and Tables

**Table 1:** Solvent study for the photochemical hydroxylation of **1a**.

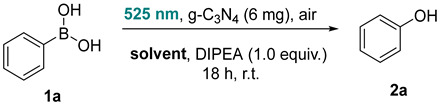			
**Entry**	**Solvent**	**Conversion (%)** ^a^	**Yield (%)** ^b^
**1**	**Acetonitrile**	**60**	**58**
2	CH_2_Cl_2_	40	34
3	CHCl_3_	10	-
4	Ethyl acetate	27	25
5	1,2-Dichloroethane	35	33
6	MeOH	13	10
7	iPrOH	5	-
8	THF	4	-
9	Dioxane	0	-
10	DMSO-d_6_	10	-
11	Acetone	5	-
12	Petroleum Ether	33	30
13	Benzene	40	31
14	Toluene	33	31
15	H_2_O	0	-

[^a^] Yield was determined by ^1^H-NMR using an internal standard. [^b^] Yield of **2a** after purification by column chromatography. The reaction was performed with phenyl boronic acid (**1a**) (60 mg, 0.50 mmol), *N*,*N*′-diisopropylethylamine (DIPEA) (65 mg, 0.50 mmol, 1.0 equiv.), and g-C_3_N_4_ (6 mg) in solvent (2 mL) under LED lamp (Kessil PR 160L, 525 nm) irradiation for 18 h. The bold values represent the best results in the table.

**Table 2:** Additive study for the photochemical hydroxylation of **1a**.

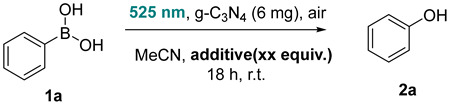			
**Entry**	**Additive (equiv.)**	**Conversion (%)** ^a^	**Yield (%)** ^b^
1	**DIPEA (1)**	**60**	**58**
**2**	DIPEA (0.75)	40	35
3	DIPEA (0.5)	25	22
4	Butylamine (1)	0	-
5	Morpholine (1)	5	-
6	Pyrrolidine (1)	1	-
7	Piperidine (1)	5	-
8	TEA (1)	0	-
9	TMEDA (1)	0	-

[^a^] Yield was determined by ^1^H-NMR using an internal standard. [^b^] Yield of **2a** after purification by column chromatography. The reaction was performed with phenyl boronic acid (**1a**) (60 mg, 0.50 mmol), additive (xx mmol, xx equiv.), and g-C_3_N_4_ (6 mg) in acetonitrile (2 mL) under LED lamp (Kessil PR 160L, 525 nm) irradiation for 18 h. The bold values represent the best results in the table.

## Data Availability

All data supporting this study are included in the article and the Supplementary Materials.

## Supplementary Materials

The following supporting information can be downloaded at: https://www.mdpi.com/article/10.3390/molecules31081371/s1, ^1^H NMR and ^13^C NMR spectra of the synthesized compounds; detailed experimental procedures, optimization studies and mechanistic experiments (PDF). References [29,30,31,32] are cited in the supplementary materials.
