# Supplementary material for: Green Light-Driven Hydroxylation of Boronic Acids Employing g-C3N4 as the Photocatalyst
Source: Molecules. 2026 Apr 21;31(8):1371. doi: 10.3390/molecules31081371 (PMC13119400; doi:10.3390/molecules31081371)
Supplement: Supplementary file 1 [file molecules-31-01371-s001.zip › molecules-4155238-supplementary.pdf]

Photooxidation of Boronic Acids employing g-C_3N_4 as the Photocatalyst

Alexandros Emmanouil Troulos,[a] Anastasia Maria Antonaki,[a] Maria Zografaki,[b,c] Vassilios Binas*[b,c] and Petros L. Gkizis*[a]

[a]*Laboratory of Organic Chemistry, Department of Chemistry, Aristotle University of Thessaloniki, University Campus 54124, Thessaloniki, Greece*

[b]*Physical Chemistry Laboratory, Department of Chemistry, Aristotle University of Thessaloniki, University Campus 54124, Thessaloniki, Greece*

[c]*Institute of Electronic Structure and Laser, Foundation for Research and Technology-Hellas (FORTH-IESL), 70013 Heraklion, Greece.*

SUPPORTING INFORMATION

	Page
General Remarks	S2
Synthesis and Characterization of g-C_3N_4	S4
Optimization of the Reaction Conditions for the Photochemical Hydroxylation of Phenyl Boronic Acid	S9
General Procedure for the Photochemical Aerobic Hydroxylation of Boronic Acids	S15
Gram-scale reaction for the Photochemical Aerobic Hydroxylation of Phenyl Boronic Acid	S24
Procedure for g-C_3N_4 Recovery and Recycling	S26
Quenching Studies on the Phenyl Boronic Acid Hydroxylation	S27
Test for the Detection of H_2O_2 Production	S28
Determination of the Quantum Yield	S31
Mechanistic Investigation with UV-Vis	S33
References	S34
NMR Spectra	S35

General Remarks

Chromatographic purification of products was accomplished using forced-flow chromatography on Merck® Kieselgel 60 70-230 mesh. Thin-layer chromatography (TLC) was performed on aluminum-backed silica plates (0.2 mm, 60 F_{254}). Visualization of the developed chromatogram was performed by fluorescence quenching using phosphomolybdic acid, anisaldehyde or potassium permanganate stains. Melting points were determined on a Buchi® 530 hot stage apparatus and are uncorrected. Mass spectra (ESI) were recorded on a Finningan® Surveyor MSQ LC-MS spectrometer. HRMS spectra were recorded on a Bruker® Maxis Impact QTOF spectrometer. ^{1}H-NMR, ^{19}F-NMR, and ^{13}C-NMR spectra were recorded on an Ascend™ Bruker 300 MHz (300 MHz, 282 MHz, and 75 MHz, respectively) or on an Agilent Technologies DD2 500 MHz (500 MHz, 470 MHz, and 125 MHz, respectively) and are internally referenced to residual solvent signals. Data for ^{1}H-NMR are reported as follows: chemical shift (δ ppm), integration, multiplicity (s = singlet, d = doublet, t = triplet, q = quartet, m = multiplet, br s = broad signal), coupling constant, and assignment. Data for ^{13}C-NMR are reported in terms of chemical shift (δ ppm). Data for ^{19}F-NMR are reported in terms of chemical shift (δ ppm) and are internally referenced to fluoroform. Mass spectra and conversions of the reactions were recorded on a Shimadzu® GC-MS-QP2010 Plus Gas Chromatograph Mass Spectrometer utilizing a MEGA® column (MEGA-5, F.T: 0.25 μm, I.D.: 0.25 mm, L': 30 m, T_{max}: 350 °C, Column ID# 11475). A Varian® Cary 50 UV-Vis spectrophotometer was used for the quantum yield measurements and the UV-Vis data. Kessil lamps PR160L were used as the irradiation source. For all experiments, the intensity of the Kessil lamps was controlled at the maximum level with power consumption: 370 nm (max 43W), 390 nm (max 52W), 427 nm (max 45W), 440 nm (max 45W), 456 nm (max 50W), 467 nm (max 44W) and 525 nm (max 44W).

Photocatalyst Characterization:

X-ray Diffraction (XRD): Patterns were obtained using a Bruker AXS D8 Advance diffractometer with a Cu anode (λ = 0.154 nm), operated at 40 kV and 40 mA.

Surface Morphology (FESEM/EDS): Analysis was performed on a JEOL JSM6390LV SEM (20 kV) and a FE-SEM (JEOL 7000) operating at 15 keV, equipped with an Oxford INCA PentaFETx3 EDS detector.

Transmission Electron Microscopy (TEM): Morphological features were studied using a JEOL-2100 instrument operating at an accelerating voltage of 200 kV.

X-ray Photoelectron Spectroscopy (XPS): Spectra were acquired using a SPECS "FlexMod XPS" with a monochromatic Al Kα source. All spectra were calibrated using the adventitious C 1s peak at 284.6 eV.

Photoluminescence (PL & TRPL): Spectra were acquired at room temperature using an Edinburgh FS5 spectrofluorometer equipped with a 150 W Xenon arc lamp (λ_{ex}c = 325 nm) and a 375 nm EPL picosecond pulsed diode laser for lifetime measurements.

Optical Properties: UV-Vis diffuse reflectance spectra (DRS) were measured on a Perkin Elmer Lambda 950 spectrometer. Quantum yield measurements and UV-Vis mechanistic data were recorded on a Varian® Cary 50 UV-Vis spectrophotometer.

Synthesis and Characterization of g-C_3N_4

The structural, morphological, and photophysical properties of the synthesized g-C_3N_4 were thoroughly investigated using XRD, TEM, FESEM/EDS, XPS, and PL/TRPL.

Crystalline Structure (XRD): The formation of polymeric carbon nitride was confirmed by X-ray diffraction, with characteristic reflections at $2\theta \approx 13.1°$ (100) and 27.4° (002). Comparative XRD analysis of the fresh and recovered catalyst (after four cycles) confirms that the crystalline framework remains intact.

Morphology (TEM & FESEM): High-resolution TEM and FESEM imaging revealed a characteristic layered, flaky morphology. FESEM images of the catalyst before and after the reaction demonstrate that the aggregated, sheet-like structure remains stable, confirming its robustness.

Surface Composition (XPS & EDS): Elemental analysis by EDS verified that C and N are the predominant elements, with the absolute absence of metallic impurities. High-resolution XPS spectra further confirm the structural and electronic integrity of the tri-s-triazine core, which is maintained after catalytic use (shifts ≤ 0.5 eV).

Photophysical Properties (PL & TRPL): Steady-state PL spectroscopy (Exc. 325 nm) shows a strong emission peak at ~440 nm, corresponding to the band-edge

transition, with a significant emission tail extending beyond 550 nm. This tail provides direct evidence of surface defect states that facilitate sub-bandgap photoactivation under 525 nm irradiation. Furthermore, Time-Resolved PL (TRPL) analysis reveals a multi-exponential decay with an average carrier lifetime of 3.15 ns. The presence of long-lived components (τ_2 = 4.25 ns) suggests that defect states act as temporary traps, prolonging the lifetime of photogenerated charge carriers and facilitating energy transfer to molecular oxygen for ROS generation.

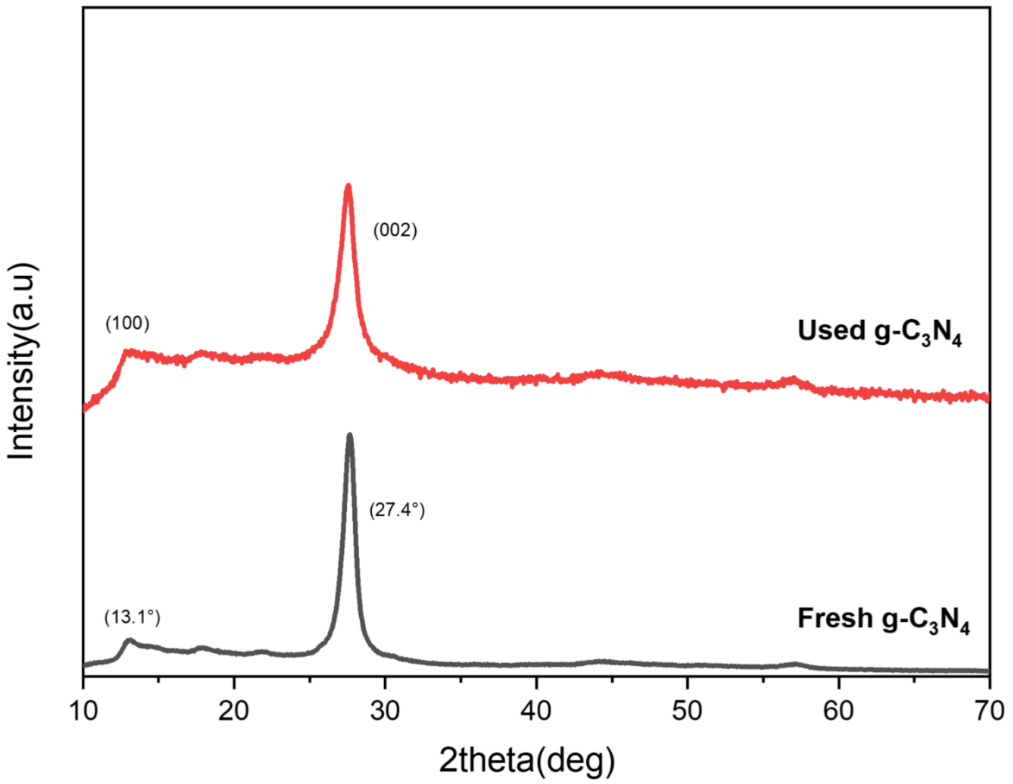

Figure S1. Comparative XRD patterns of fresh (black line) and used (red line) g-C_3N_4 photocatalyst after four catalytic cycles. The preservation of the characteristic reflections at 2θ ≈ 13.1° (100) and 27.4° (002) confirms the structural integrity and crystalline stability of the polymeric framework under the reaction conditions.

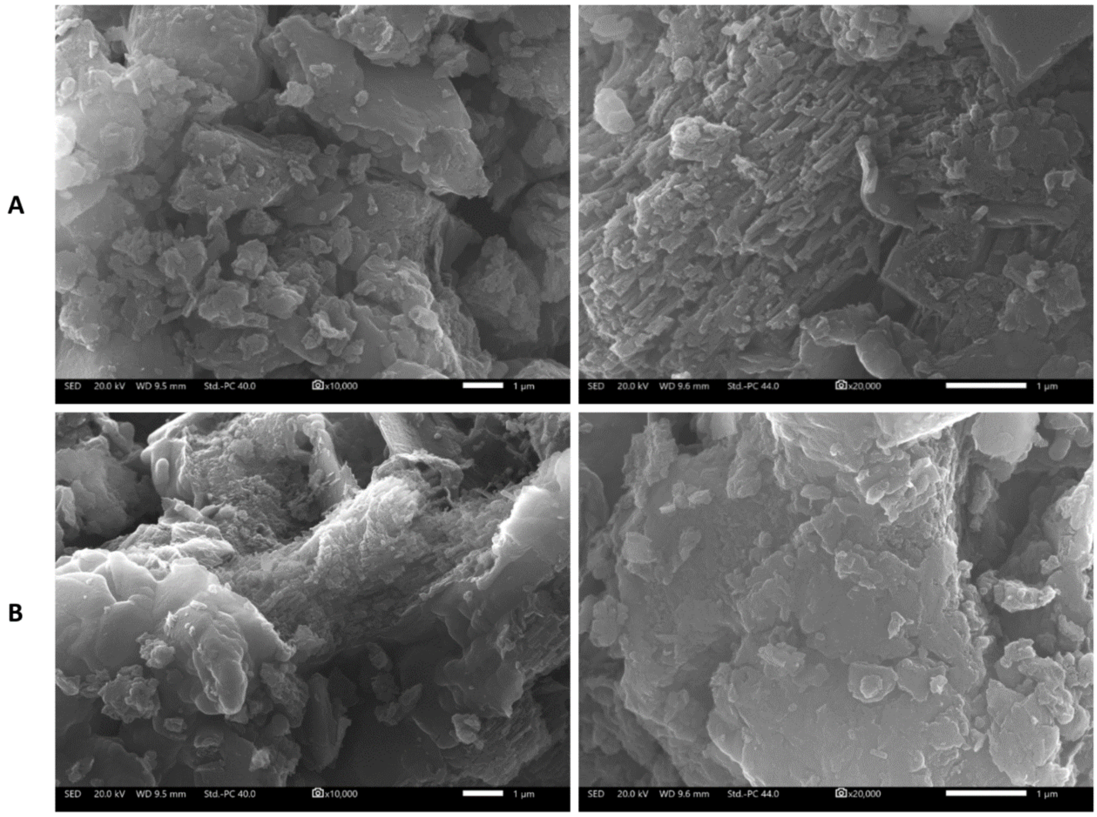

Figure S2. FESEM micrographs of g-C_3N_4: (**A**) fresh catalyst and (**B**) recovered catalyst after four catalytic cycles. Both samples exhibit the characteristic aggregated, sheet-like layered structures, confirming the morphological stability of the photocatalyst during the reaction.

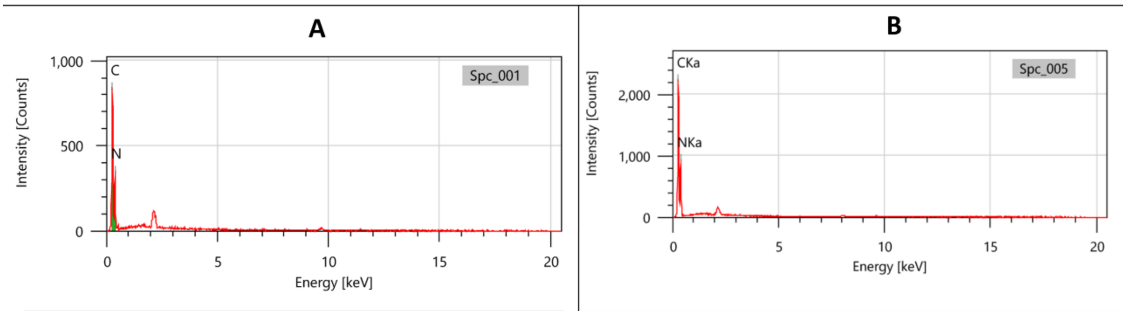

Element	Line	Mass%	Atom%
C	K	30.64±0.38	34.00±0.42
N	K	69.36±2.05	66.00±1.95
Total		100.00	100.00
Spc_001			Fitting ratio 0.1490

Element	Line	Mass%	Atom%
C	K	30.43±0.24	33.77±0.26
N	K	69.57±1.27	66.23±1.21
Total		100.00	100.00
Spc_005			Fitting ratio 0.0717

Figure S3. EDS spectra and elemental composition of: (**A**) fresh *g-C_3N_4* and (**B**) recovered *g-C_3N_4*. The results confirm the stability of the C/N ratio and the absolute absence of metallic impurities in both samples.

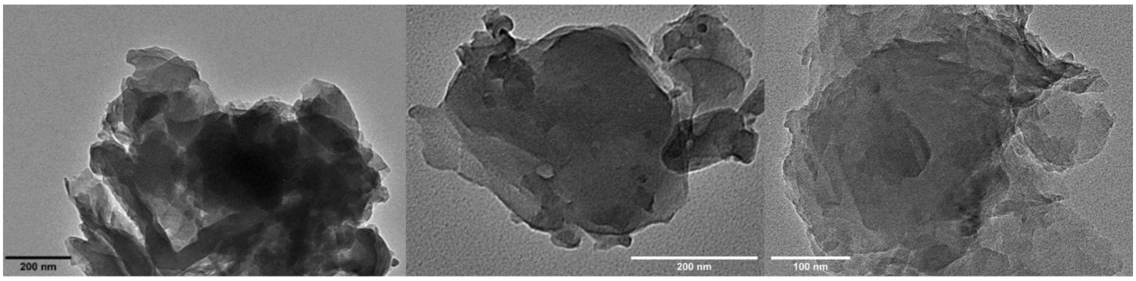

Figure S4. High-resolution TEM images of the synthesized g-C_3N_4, revealing the flaky, layered morphology and thin nanosheet structure of the polymeric framework.

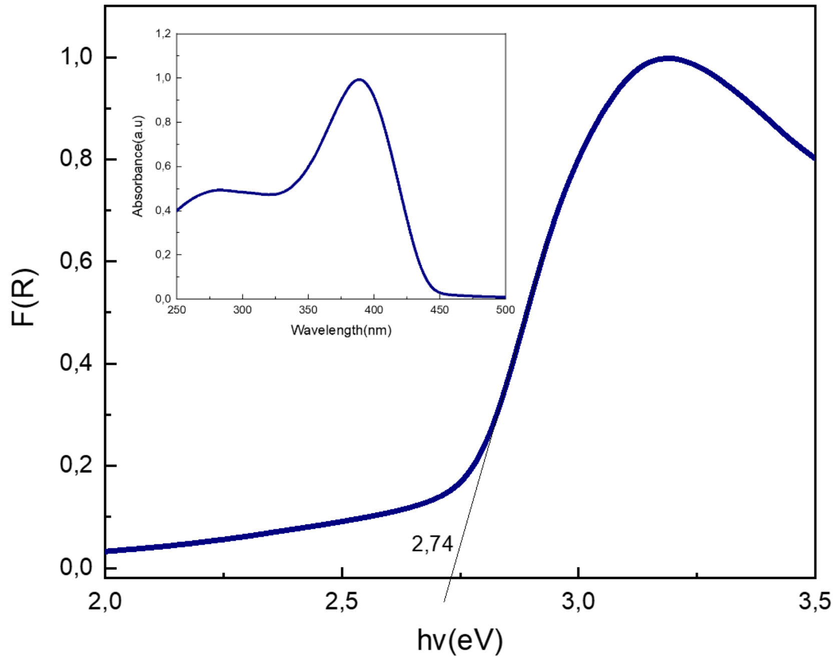

Figure S5. UV–Vis diffuse reflectance spectrum of g-C_3N_4 and corresponding Tauc plot used for the estimation of the optical band gap (Eg = 2.74 eV).

a)

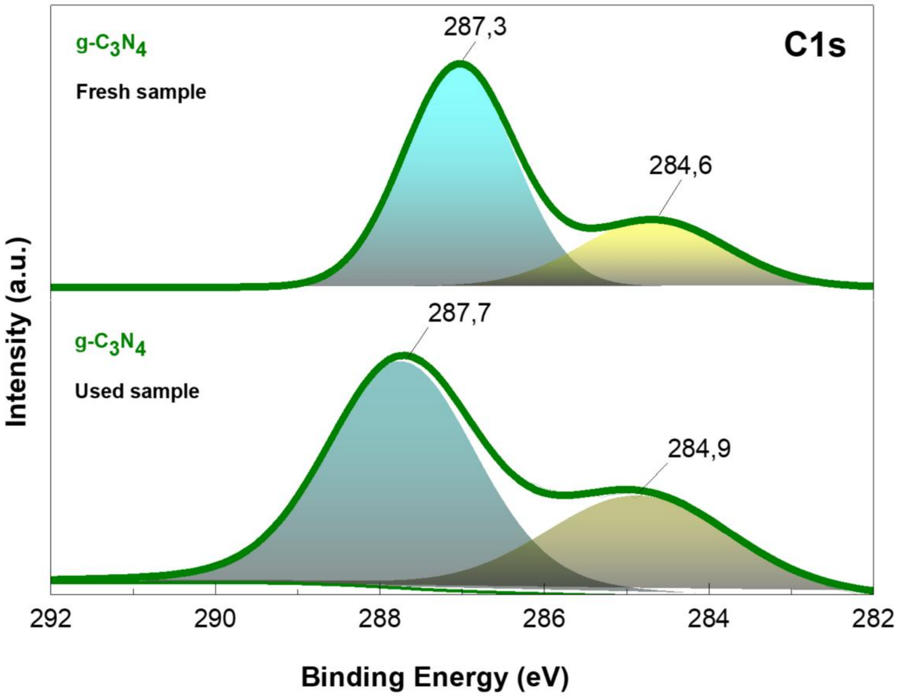
g-C3N4
Fresh sample
287,3
C1s
284,6
g-C3N4
Used sample
287,7
284,9
Intensity (a.u.)
292
290
288
286
284
282
Binding Energy (eV)

b)

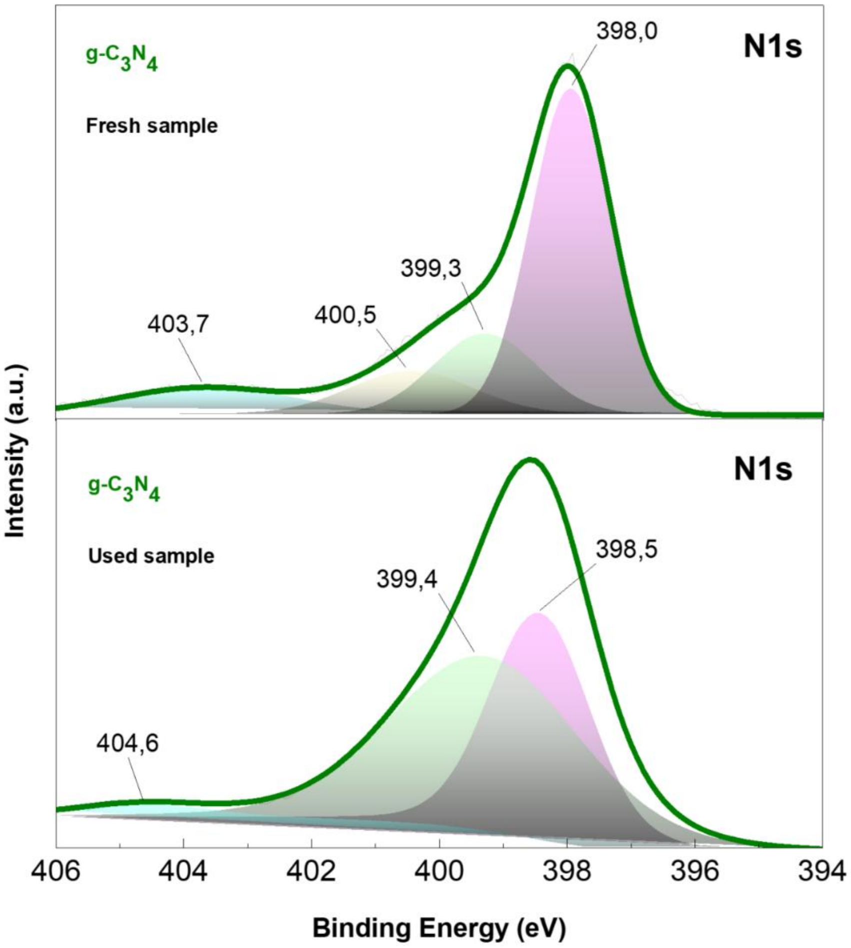
g-C3N4
Fresh sample
398,0
N1s
399,3
400,5
403,7
g-C3N4
Used sample
N1s
398,5
399,4
404,6
Intensity (a.u.)
406
404
402
400
398
396
394
Binding Energy (eV)

Figure S6. High-resolution XPS spectra of fresh and used g-C_3N_4 catalyst: (a) C 1s region and (b) N 1s region. The binding energy values for the tri-s-triazine framework (C 1s at ~287.3 eV and N 1s at ~398.0 eV) remain essentially consistent after four catalytic cycles (shifts ≤ 0.5 eV). The deconvolution of the N 1s region in the used sample shows a merging of the tertiary and amino nitrogen signals (~399.4 eV), attributed to subtle surface charging and interactions with the reaction medium, while the overall structural and electronic integrity of the photocatalyst is preserved.

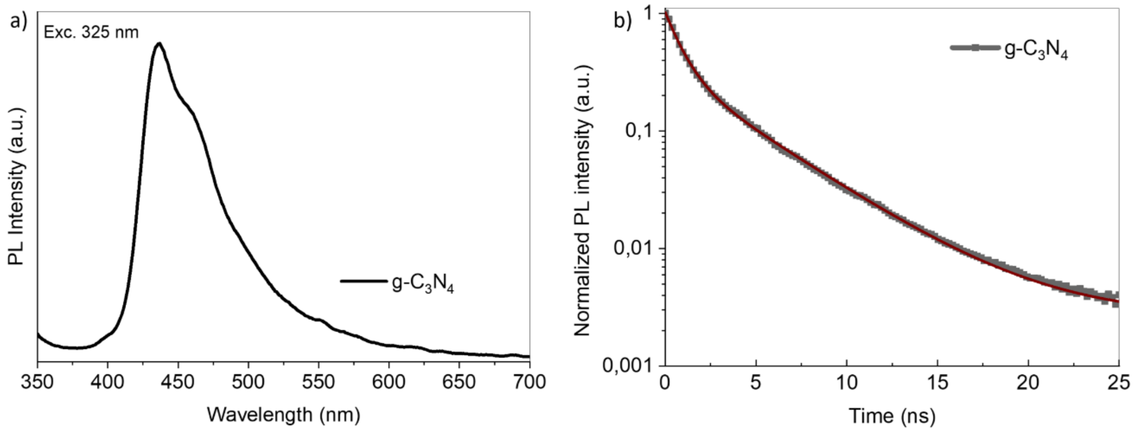

Figure S7. (a) Steady-state photoluminescence (PL) spectrum (Excitation at 325 nm) of g-C_3N_4, showing a significant emission tail into the visible region. (b) Time-resolved PL (TRPL) decay profile of g-C_3N_4 fitted with a biexponential model.

Sample	α_1 (%)	τ_1 (ns)	α_2 (%)	τ_2 (ns)	τ_{av} (ns)
g-C_3N_4	70.2	0.86	29.8	4.25	3.15

Table S1. PL lifetime biexponential decay model fitting parameters and calculated average carrier lifetime (τ_{av}) for the g-C_3N_4 photocatalyst.

Optimization of the Reaction Conditions for the Photochemical Hydroxylation of Phenyl Boronic Acid

Irradiation Source Control Study

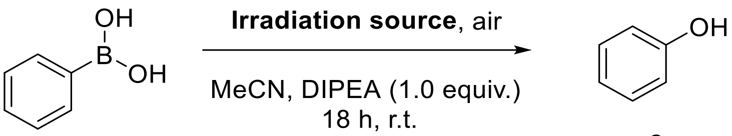

1a 2a

Entry	Irradiation Wavelength (nm)	Yield (%)[a]
1	370	100 (92)
2	390	83 (80)
3	427	60 (55)
4	440	61 (54)
5	456	60 (54)
6	467	58 (48)
7	525	0
8	Dark	0

[a] Yield was determined by ^{1}H-NMR, using internal standard. Yield of **2a** after purification by column chromatography is presented in parenthesis. The reaction was performed with phenyl boronic acid (**1a**) (60 mg, 0.50 mmol) and *N,N'*-diisopropylethylamine (DIPEA) (65 mg, 0.50 mmol, 1.0 equiv.) in acetonitrile (2 mL) under irradiation for 18 h.

Solvent Study

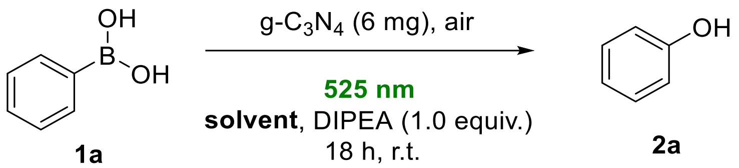

Entry	Solvent	Conversion (%)[a]	Yield (%)[b]
1	Acetonitrile	60	58
2	CH_2Cl_2	40	34
3	$CHCl_3$	10	-
4	Ethyl acetate	27	25
5	1,2-Dichloroethane	35	33
6	MeOH	13	10
7	iPrOH	5	-
8	THF	4	-
9	Dioxane	0	-
10	DMSO-d_6	10	-
11	Acetone	5	-
12	Petroleum Ether	33	30
13	Benzene	40	31
14	Toluene	33	31
15	H_2O	0	-

[a] Yield was determined by ^{1}H-NMR, using internal standard. [b] Yield of **2a** after purification by column chromatography. The reaction was performed with phenyl boronic acid (**1a**) (60 mg, 0.50 mmol), *N,N'*-diisopropylethylamine (DIPEA) (65 mg, 0.50 mmol, 1.0 equiv.) and g-C_3N_4 (6 mg) in solvent (2 mL) under LED lamps (Kessil PR 160L, 525 nm) irradiation for 18 h.

Additive Study

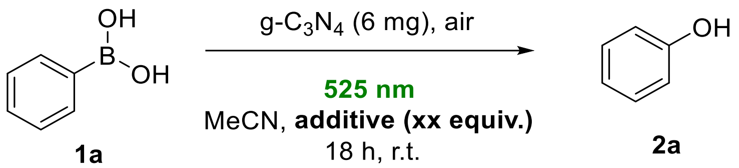

Entry	Additive (equiv.)	Conversion (%)[a]	Yield (%)[b]
1	TEA (1)	0	-
2	DIPEA (1)	60	58
3	DIPEA (0.75)	40	35
4	DIPEA (0.5)	25	22
5	Morpholine (1)	5	-
6	Pyrrolidine (1)	1	-
7	Piperidine (1)	5	-
8	TMEDA (1)	0	-
9	Butylamine (1)	0	-

[a] Yield was determined by ^{1}H-NMR, using internal standard. [b] Yield of **2a** after purification by column chromatography. The reaction was performed with phenyl boronic acid (**1a**) (60 mg, 0.50 mmol), additive (xx mmol, xx equiv.) and g-C_3N_4 (6 mg) in acetonitrile (2 mL) under LED lamps (Kessil PR 160L, 525 nm) irradiation for 18 h.

Reaction Concentration Study

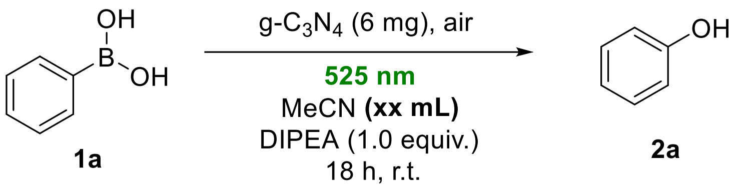

Entry	Solvent (mL)	Conversion (%)[a]	Yield (%)[b]
1	0.5	23	23
2	1.0	30	28
3	1.5	47	45
4	2.0	60	58

[a] Yield was determined by ^{1}H-NMR, using internal standard. [b] Yield of **2a** after purification by column chromatography. The reaction was performed with phenyl boronic acid (**1a**) (60 mg, 0.50 mmol), *N,N'*-diisopropylethylamine (DIPEA) (65 mg, 0.50 mmol, 1.0 equiv.) and g-C_3N_4 (6 mg) in acetonitrile (xx mL) under LED lamps (Kessil PR 160L, 525 nm) irradiation for 18 h.

Catalyst Loading Study

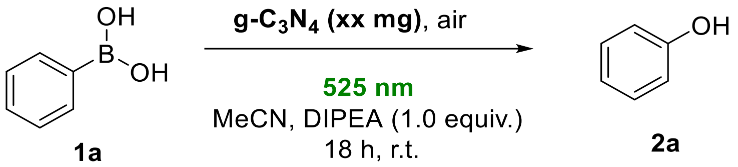

Entry	Catalyst Loading (mg)	Yield (%)[a]
1	1	0
2	2	5 (4)
3	4	15 (10)
4	6	60 (58)
5	8	65 (62)
**6**	**10**	**100 (92)**

[a] Yield was determined by ^{1}H-NMR, using internal standard. Yield of **2a** after purification by column chromatography is presented in parenthesis. The reaction was performed with phenyl boronic acid (**1a**) (60 mg, 0.50 mmol), *N,N'*-diisopropylethylamine (DIPEA) (65 mg, 0.50 mmol, 1.0 equiv.) and g-C_3N_4 (xx mg) in acetonitrile (2 mL) under LED lamps (Kessil PR 160L, 525 nm) irradiation for 18 h.

Control Experiments

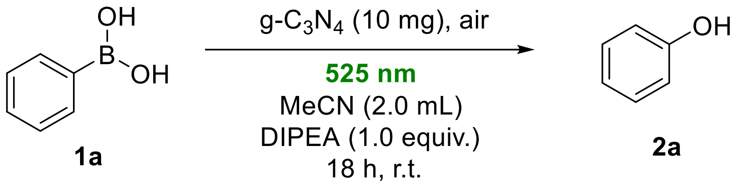

Entry	Deviation	Additive (equiv.)	Conversion (%)
1	525 nm	-	0
2	No catalyst	DIPEA (1)	0
3	No irradiation	DIPEA (1)	0
4	Heat at 60 °C	DIPEA (1)	0
5	Argon	DIPEA (1)	3
6	Foil	DIPEA (1)	0
7	No irradiation	H_2O_2 (5)	100

[a] Conversion was determined by ^{1}H-NMR.

General Procedure for the Photochemical Aerobic Hydroxylation of Boronic Acids

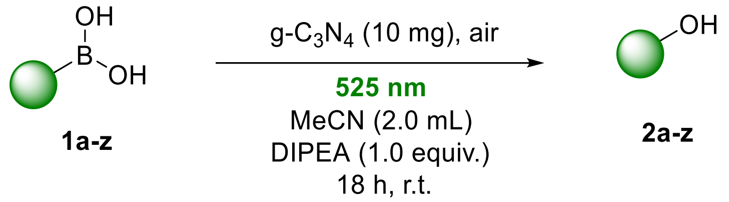

In a glass vial containing the corresponding boronic acid (0.50 mmol) and *N,N'*-diisopropylethylamine (65 mg, 0.50 mmol), g-C_3N_4 (10 mg) were added in acetonitrile (2.0 mL). The reaction mixture was left under vigorous stirring under LED lamps (Kessil PR 160L, 525 nm) irradiation for 18 h. Upon reaction completion, the solvent was removed *in vacuo*. The desired product was purified by column chromatography (Pet. Ether/AcOEt: 6:1).

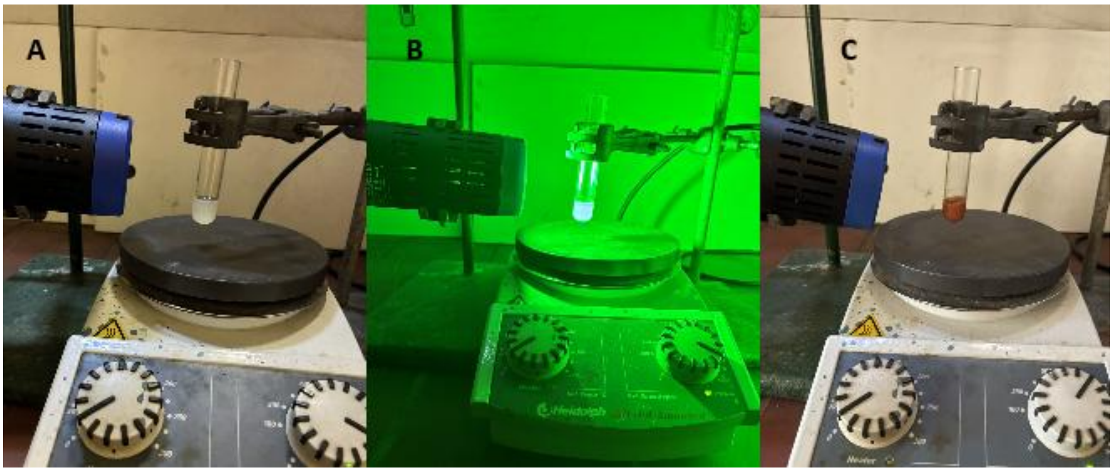

A. Reaction setup, the reaction mixture is placed 3 cm away from the irradiation source; **B.** Reaction mixture under LED lamps (Kessil PR 160L, 525 nm) irradiation; **C.** Reaction mixture upon reaction completion.

Phenol (2a)[1]

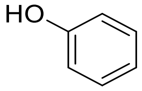

Yield: **92%**; White solid; m.p.: 41-42 °C; Eluent: Petroleum ether / Ethyl acetate 6:1.
NMR data in accordance with reported literature.[1] **^{1}H NMR** (300 MHz, $CDCl_3$) δ: 7.25 (2H, t, J = 7.5 Hz, ArH), 6.94 (1H, t, J = 7.5 Hz, ArH), 6.85 (2H, d, J = 7.5 Hz, ArH), 4.28 (1H, br s, OH); **^{13}C NMR** (75 MHz, $CDCl_3$) δ: 155.5, 129.7, 120.8, 115.3; **MS** (ESI) *m/z* 93 $[M-H]^-$.

_p_-Cresol (2b)[1]

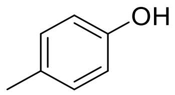

Yield: **70%**; White solid; m.p.: 33-35 °C; Eluent: Petroleum ether / Ethyl acetate 6:1. NMR data in accordance with reported literature.[1] **^{1}H NMR** (500 MHz, $CDCl_3$) *δ*: 7.06 (2H, d, *J* = 8.2 Hz, ArH), 6.78 (2H, d, *J* = 8.2 Hz, ArH), 5.44 (1H, br s, OH), 2.30 (1H, s, CH_3); **^{13}C NMR** (125 MHz, $CDCl_3$) *δ*: 153.6, 130.4, 130.3, 115.4, 20.8; **MS** (ESI) *m/z* 107 $[M-H]^-$.

4-_tert_-Butylphenol (2c)[2]

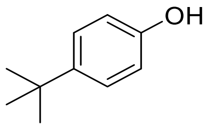

Yield: **78%**; White solid; m.p.: 90-92 °C; Eluent: Petroleum ether / Ethyl acetate 6:1. NMR data in accordance with reported literature.[2] **^{1}H NMR** (300 MHz, $CDCl_3$) *δ*: 7.28 (2H, d, *J* = 7.0 Hz, ArH), 6.80 (2H, d, *J* = 7.0 Hz, ArH), 4.95 (1H, br s, OH), 1.32 (9H, s, CH_3); **^{13}C NMR** (75 MHz, $CDCl_3$) *δ*: 153.0, 143.6, 126.5, 114.8, 34.1, 31.5; **MS** (ESI) *m/z* 150 $[M]^+$.

4-Nitrophenol (2d)[1]

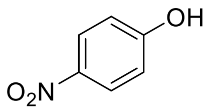

Yield: **76%**; Yellow solid; m.p.: 110-112 °C; Eluent: Petroleum ether / Ethyl acetate 6:1. NMR data in accordance with reported literature.[1] **^{1}H NMR** (300 MHz, $CDCl_3$) *δ*: 8.18 (2H, d, *J* = 9.1 Hz, ArH), 6.93 (2H, d, *J* = 9.1 Hz, ArH), 5.78 (1H, br s, OH); **^{13}C NMR** (75 MHz, $CDCl_3$) *δ*: 116.7, 142.0, 126.6, 116.0; **MS** (ESI) *m/z* 140 $[M+H]^+$.

4-(Trifluoromethyl)phenol (2e)[1]

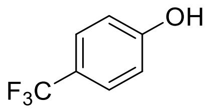

Yield: **60%**; White solid; m.p.: 40-42 °C; Eluent: Petroleum ether / Ethyl acetate 6:1. NMR data in accordance with reported literature.[3] **^{1}H NMR** (500 MHz, $CDCl_3$) *δ*: 7.51 (2H, d, *J* = 8.4 Hz, ArH), 6.90 (2H, d, *J* = 8.4 Hz, ArH), 5.38 (1H, br s, OH); **^{13}C NMR** (125 MHz, $CDCl_3$) *δ*: 158.6, 127.3 (q, *J* = 3.8 Hz), 124.5 (q, *J* = 271.0 Hz), 123.2 (q, *J* = 32.7 Hz), 115.8; **^{19}F NMR** (470 MHz, $CDCl_3$) δ: -61.5; **MS** (ESI) *m/z* 161 $[M-H]^-$.

4-Hydroxybenzonitrile (2f)[1]

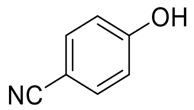

Yield: **89%**; Brown solid; m.p.: 98-100 °C; Eluent: Petroleum ether / Ethyl acetate 6:1.
NMR data in accordance with reported literature.[1] **^{1}H NMR** (500 MHz, $CDCl_3$) *δ*: 7.54 (2H, d, *J* = 8.9 Hz, ArH), 6.94 (2H, d, *J* = 8.9 Hz, ArH); **^{13}C NMR** (125 MHz, $CDCl_3$) *δ*: 160.7, 134.7, 119.6, 116.8, 103.3; **MS** (ESI) *m/z* 120 $[M+H]^+$.

4-Bromophenol (2g)[3]

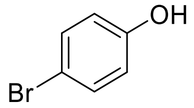

Yield: **85%**; Red solid; m.p.: 66-68°C; Eluent: Petroleum ether / Ethyl acetate 6:1.
NMR data in accordance with reported literature.[3] **^{1}H NMR** (300 MHz, $CDCl_3$) *δ*: 7.34 (2H, d, *J* = 8.9 Hz, ArH), 6.73 (2H, d, *J* = 8.9 Hz, ArH); **^{13}C NMR** (75 MHz, $CDCl_3$) *δ*: 154.9, 132.9, 117.6, 113.4; **MS** (ESI) *m/z* 172/174 $[M]^+$.

4-Fluorophenol (2h)[1]

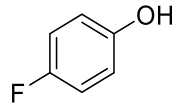

Yield: **77%**; Red solid; m.p.: 41-43 °C; Eluent: Petroleum ether / Ethyl acetate 6:1.
NMR data in accordance with reported literature.[1] **^{1}H NMR** (500 MHz, $CDCl_3$) *δ*: 6.91 (2H, t, *J* = 8.5 Hz, ArH), 6.78 (2H, dd, *J* = 8.5 and 4.2 Hz, ArH), 4.14 (1H, br s, OH); **^{13}C NMR** (125 MHz, $CDCl_3$) *δ*: 157.3 (d, *J* = 237.9 Hz), 151.4 (d, *J* = 2.1 Hz), 116.3 (d, *J* = 8.0 Hz), 116.0 (d, *J* = 23.2 Hz); **^{19}F NMR** (470 MHz, $CDCl_3$) δ: -124.2; **MS** (ESI) *m/z* 111 $[M-H]^-$.

4-Methoxyphenol (2i)[1]

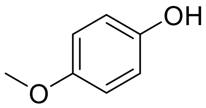

Yield: **80%**; White solid; m.p.: 55-58 °C; Eluent: Petroleum ether / Ethyl acetate 6:1.
NMR data in accordance with reported literature.[1] **^{1}H NMR** (300 MHz, $CDCl_3$) *δ*: 6.84 (4H, m, ArH), 3.76 (3H, s, OCH_3); **^{13}C NMR** (75 MHz, $CDCl_3$) *δ*: 153.7, 148.4, 116.2, 115.0, 56.0; **MS** (ESI) *m/z* 123 $[M-H]^-$.

_m_-Cresol (2j)[1]

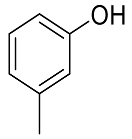

Yield: **76%**; Colorless oil; Eluent: Petroleum ether / Ethyl acetate 6:1.

NMR data in accordance with reported literature.[1] **^{1}H NMR** (500 MHz, $CDCl_3$) *δ*: 7.14 (1H, t, *J* = 7.7 Hz, ArH), 6.78 (1H, d, *J* = 7.7 Hz, ArH), 6.70-6.64 (2H, d, *J* = 7.7 Hz ArH), 5.31 (1H, br s, OH), 2.32 (3H, s, CH_3); **^{13}C NMR** (125 MHz, $CDCl_3$) *δ*: 155.7, 140.2, 129.8, 122.0, 116.4, 112.7, 21.6; **MS** (ESI) *m/z* 107 $[M-H]^-$.

_o_-Cresol (2k)[2]

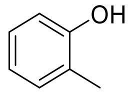

Yield: **78%**; Colorless oil; Eluent: Petroleum ether / Ethyl acetate 6:1.

NMR data in accordance with reported literature.[2] **^{1}H NMR** (500 MHz, $CDCl_3$) *δ*: 7.13 (1H, d, *J* = 7.5 Hz, ArH), 7.09 (1H, d, *J* = 7.5 Hz, ArH), 6.85 (1H, t, *J* = 7.5 Hz, ArH), 6.78 (1H, d, *J* = 7.5 Hz, ArH), 4.70 (1H, br s, OH), 2.26 (3H, s, CH_3); **^{13}C NMR** (125 MHz, $CDCl_3$) *δ*: 153.8, 131.1, 127.2, 123.7, 120.8, 114.9, 15.7; **MS** (ESI) *m/z* 107 $[M-H]^-$.

3-Bromophenol (2l)[1]

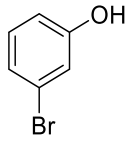

Yield: **63%**; Colorless oil; Eluent: Petroleum ether / Ethyl acetate 6:1.

NMR data in accordance with reported literature.[1] **^{1}H NMR** (500 MHz, $CDCl_3$) *δ*: 7.12-7.06 (2H, m, ArH), 7.03-7.02 (1H, m, ArH), 6.79-6.76 (1H, m, ArH), 4.69 (1H, br, OH); **^{13}C NMR** (125 MHz, $CDCl_3$) *δ*: 156.6, 131.2, 124.4, 123.1, 119.2, 114.6; **MS** (ESI) *m/z* 172/174 $[M]^+$.

2-Bromophenol (2m)[1]

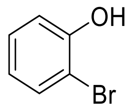

Yield: **70%**; Yellow oil; Eluent: Petroleum ether / Ethyl acetate 6:1.

NMR data in accordance with reported literature.[1] **^{1}H NMR** (500 MHz, $CDCl_3$) *δ*: 7.47 (1H, d, *J* = 8.1 Hz, ArH), 7.22 (1H, t, *J* = 8.1 Hz, ArH), 7.03 (1H, d, *J* = 8.1 Hz, ArH), 6.81 (1H, t, *J* = 8.1 Hz, ArH), 5.46 (1H, br s, OH); **^{13}C NMR** (125 MHz, $CDCl_3$) *δ*: 152.6, 132.4, 129.5, 122.2, 116.5, 110.6; **MS** (ESI) *m/z* 172/174 $[M]^+$.

3-Chlorophenol (2n)[1]

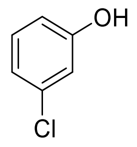

Yield: **71%**; White solid; m.p.: 30-33 °C; Eluent: Petroleum ether / Ethyl acetate 6:1.
NMR data in accordance with reported literature.[1] **^{1}H NMR** (500 MHz, $CDCl_3$) *δ*: 7.16 (1H, t, *J* = 8.1 Hz, ArH), 6.92 (1H, d, *J* = 8.1 Hz, ArH), 6.86 (1H, s, ArH), 6.72 (1H, d, *J* = 8.1, ArH), 5.07 (1H, br s, OH); **^{13}C NMR** (125 MHz, $CDCl_3$) *δ*: 156.6, 135.3, 130.8, 121.5, 116.3, 114.1; **MS** (ESI) *m/z* 127/129 $[M-H]^-$.

2-Chlorophenol (2n)[1]

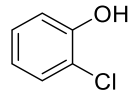

Yield: **58%**; Colorless oil; Eluent: Petroleum ether / Ethyl acetate 6:1.
NMR data in accordance with reported literature.[1] **^{1}H NMR** (500 MHz, $CDCl_3$) *δ*: 7.29 (1H, d, *J* = 8.2 Hz, ArH), 7.20-7.16 (1H, m, ArH), 7.00 (1H, d, *J* = 8.2 Hz, ArH), 6.85 (1H, t, *J* = 8.2 Hz, ArH), 5.55 (1H, br s, OH); **^{13}C NMR** (125 MHz, $CDCl_3$) *δ*: 151.6, 129.3, 128.7, 121.6, 120.2, 116.5; **MS** (ESI) *m/z* 127/129 $[M-H]^-$.

2-Iodophenol (2p)[1]

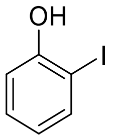

Yield: **69%**; Yellow oil; Eluent: Petroleum ether / Ethyl acetate 6:1.
NMR data in accordance with reported literature.[1] **^{1}H NMR** (500 MHz, $CDCl_3$) *δ*: 7.65 (1H, dd, *J* = 7.9 and 1.5 Hz, ArH), 7.26 -7.22 (1H, m, ArH), 7.00 (1H, dd, *J* = 7.9 and 1.5 Hz, ArH), 6.69-6.65 (1H, m, ArH), 5.28 (1H, br s, OH); **^{13}C NMR** (125 MHz, $CDCl_3$) *δ*: 155.2, 138.6, 130.6, 122.8, 115.5, 86.1; **MS** (ESI) *m/z* 221 $[M+H]^+$.

3-Fluorophenol (2q)[4]

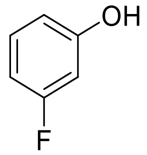

Yield: **68%**; Orange solid; m.p.: 150-152 °C; Eluent: Petroleum ether / Ethyl acetate 6:1.
NMR data in accordance with reported literature.[4] **^{1}H NMR** (500 MHz, $CDCl_3$) *δ*: 7.21-7.15 (1H, m, ArH), 6.66-6.56 (3H, m, ArH), 4.87 (1H, br s, OH); **^{13}C NMR** (125 MHz, $CDCl_3$) *δ*: 163.8 (d, *J* = 245.5 Hz), 159.6 (d, *J* = 11.3 Hz), 130.6 (d, *J* = 10.1 Hz), 111.2 (d, *J* = 2.9 Hz), 107.9 (d, *J* = 21.2 Hz), 103.4 (d, *J* = 24.5 Hz); **^{19}F NMR** (470 MHz, $CDCl_3$) δ: -111.8; **MS** (ESI) *m/z* 111 $[M-H]^-$.

2-Fluorophenol (2r)[1]

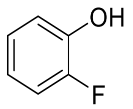

Yield: **89%**; Colorless oil; Eluent: Petroleum ether / Ethyl acetate 6:1.
NMR data in accordance with reported literature.[1] **^{1}H NMR** (500 MHz, $CDCl_3$) *δ*: 7.10-6.98 (3H, m, ArH), 6.88-6.82 (1H, m, ArH), 5.10 (1H, br s, OH); **^{13}C NMR** (125 MHz, $CDCl_3$) *δ*: 151.2 (d, *J* = 237.3 Hz), 143.9 (d, *J* = 14.2 Hz), 125.1 (d, *J* = 3.8 Hz), 121.1 (d, *J* = 6.5 Hz), 117.6, 115.8 (d, *J* = 18.0 Hz); **^{19}F NMR** (470 MHz, $CDCl_3$) δ: -141.4; **MS** (ESI) *m/z* 111 $[M-H]^-$.

2-Hydroxybenzonitrile (2s)[1]

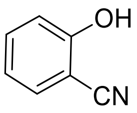

Yield: **71%**; Yellow solid; m.p.: 88-90 °C; Eluent: Petroleum ether / Ethyl acetate 6:1.

NMR data in accordance with reported literature.[1] **^{1}H NMR** (500 MHz, $CDCl_3$) *δ*: 7.51-7.44 (2H, m, ArH), 7.02 (2H, d, *J* = 8.4 Hz, ArH), 6.82 (1H, br s, OH); **^{13}C NMR** (125 MHz, $CDCl_3$) *δ*: 159.0, 135.1, 133.3, 121.3, 117.0, 116.8, 99.8; **MS** (ESI) *m/z* 120 $[M+H]^+$.

4-Formylphenol (2t)[5]

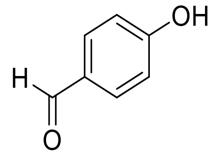

Yield: **71%**; Brown solid; m.p.: 114-116 °C; Eluent: Petroleum ether / Ethyl acetate 6:1. NMR data in accordance with reported literature.[5] **^{1}H NMR** (300 MHz, $CDCl_3$) δ: 9.85 (1H, s, CHO), 7.82 (2H, d, J = 8.5 Hz, ArH), 6.98 (2H, d, J = 8.5 Hz, ArH), 6.56 (1H, br s, OH); **^{13}C NMR** (75 MHz, $CDCl_3$) δ: 191.3, 161.6, 132.6, 129.9, 116.0; **MS** (ESI) *m/z* 121 [M-H]$^-$.

Naphthalen-1-ol (2u)[1]

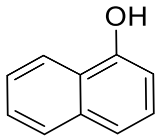

Yield: **69%**; White solid; m.p.: 118-120 °C; Eluent: Petroleum ether / Ethyl acetate 6:1.

NMR data in accordance with reported literature.[1] **^{1}H NMR** (500 MHz, $CDCl_3$) δ: 8.20-8.18 (1H, m, ArH), 7.84-7.81 (1H, m, ArH), 7.51-7.49 (2H, m, ArH), 7.45 (1H, d, J = 7.8 Hz, ArH), 7.31 (1H, t, J = 7.8 Hz, ArH), 6.82 (1H, d, J = 7.8 Hz, ArH), 5.30 (1H, br s, OH); **^{13}C NMR** (125 MHz, $CDCl_3$) δ: 151.7, 135.1, 128.0, 126.8, 126.2, 125.6, 124.7, 121.9, 121.1, 109.0; **MS** (ESI) *m/z* 143 [M-H]$^-$.

Naphthalen-2-ol (2v)[1]

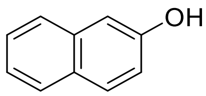

Yield: **76%**; White solid; m.p.: 90-92 °C; Eluent: Petroleum ether / Ethyl acetate 6:1.

NMR data in accordance with reported literature.[1] **^{1}H NMR** (500 MHz, $CDCl_3$) δ: 7.77 (2H, t, J = 8.5 Hz, ArH), 7.68 (1H, d, J = 8.5 Hz, ArH), 7.44 (1H, t, J = 8.5 Hz, ArH), 7.34 (1H, t, J = 8.5 Hz, ArH), 7.13 (2H, t, J = 8.5 Hz, ArH), 4.49 (1H, br s, OH); **^{13}C NMR** (125 MHz, $CDCl_3$) δ: 153.7, 134.9, 130.2, 129.3, 128.1, 126.7, 126.7, 124.0, 118.1, 109.9; **MS** (ESI) *m/z* 143 [M-H]$^-$.

Pyren-1-ol (2w)[1]

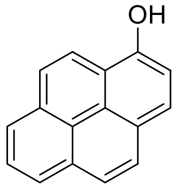

Yield: **48%**; Brown solid; m.p.: 168-170 °C; Eluent: Petroleum ether / Ethyl acetate 6:1.

NMR data in accordance with reported literature.[1] **^{1}H NMR** (500 MHz, $CDCl_3$) *δ*: 8.34 (1H, d, *J* = 8.9 Hz, ArH), 8.11 (2H, d, *J* = 7.3 Hz, ArH), 8.04 (2H, t, *J* = 8.3 Hz, ArH), 7.97 (2H, dd, *J* = 8.3 and 7.3 Hz, ArH), 7.90 (1H, d, *J* = 8.9 Hz, ArH), 7.47 (1H, d, *J* = 8.3 Hz, ArH), 5.57 (1H, br s, OH); **^{13}C NMR** (125 MHz, $CDCl_3$) *δ*: 149.7, 131.8, 131.7, 127.3, 126.6, 126.2, 126.1, 125.7, 125.5, 125.0, 124.5, 124.3, 120.5, 118.7, 113.1; **MS** (ESI) *m/z* 219 $[M+H]^+$.

Pyridin-3-ol (2x)[1]

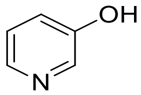

Yield: **67%**; White solid; m.p.: 119-121 °C; Eluent: Dichloromethane / Methanol 9:1.

NMR data in accordance with reported literature.[1] **^{1}H NMR** (500 MHz, $CDCl_3$) *δ*: 8.32 (1H, d, *J* = 2.8 Hz, ArH), 8.09 (1H, dd, *J* = 4.7 and 1.3 Hz, ArH), 7.36 (1H, ddd, *J* = 8.4, 2.8 and 1.4 Hz, ArH), 7.29 (1H, dd, *J* = 8.4 and 4.7 Hz, ArH), 5.76 (1H, br s, OH); **^{13}C NMR** (125 MHz, $CDCl_3$) *δ*: 155.3, 138.6, 136.0, 125.6, 125.3; **MS** (ESI) *m/z* 96 $[M+H]^+$.

Cyclohexanol (2y)[1]

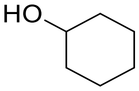

Yield: **85%**; Colorless oil; Eluent: Petroleum ether / Ethyl acetate 6:1.

NMR data in accordance with reported literature.[1] **^{1}H NMR** (300 MHz, $CDCl_3$) *δ*: 3.61-3.58 (1H, m, C*H*OH), 1.91-1.86 (2H, m, 2 x C*H*H), 1.76-1.70 (2H, m, C*H*H and OH), 1.56-1.51 (1H, m, C*H*H), 1.30-1.23 (6H, m, 6 x C*H*H); **^{13}C NMR** (75 MHz, $CDCl_3$) *δ*: 69.9, 35.3, 25.3, 24.0; **MS** (ESI) *m/z* 101 $[M+H]^+$.

1-Dodecanol (2z)[1]

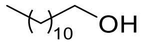

Yield: **59%**; Colorless oil; Eluent: Petroleum ether / Ethyl acetate 4:1.

NMR data in accordance with reported literature.[1] **^{1}H NMR** (300 MHz, $CDCl_3$) δ: 3.63 (2H, t, *J* = 6.4 Hz, CH_2), 1.58-1.56 (2H, m, CH_2), 1.32-1.21 (18H, m, 9 x CH_2), 0.88 (3H, t, *J* = 6.4 Hz, CH_3); **^{13}C NMR** (75 MHz, $CDCl_3$) δ: 63.1, 32.8, 31.9, 29.6, 29.6, 29.6, 29.6, 29.4, 29.3, 25.7, 22.7, 14.1; **MS** (ESI) *m/z* 186 $[M]^+$.

Gram-scale reaction for the Photochemical Aerobic Hydroxylation of Phenyl Boronic Acid

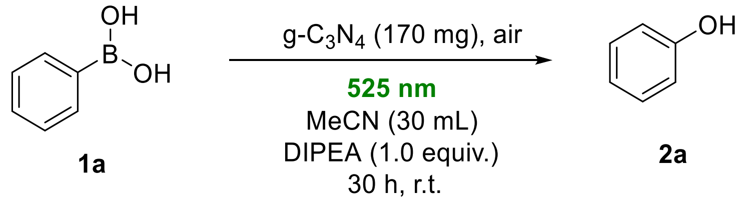

In a glass vial containing phenyl boronic acid (**1a**) (1.00 g, 8.20 mmol), *N,N'*-diisopropylethylamine (1.09 g, 8.20 mmol), g-C_3N_4 (170 mg) were added in acetonitrile (30 mL). The reaction mixture was left under vigorous stirring under LED lamps (Kessil PR 160L, 525 nm) irradiation for 30 h. Upon reaction completion, the solvent was removed in vacuo. The crude residue was diluted with EtOAc (10 mL) and washed with aq. HCl (10%v/v) (10 mL) and water (10 mL). The organic layer was dried (Na_2SO_4), filtered and the solvent was removed in vacuo to afford **700 mg** of **2a**. **Yield: 91%.**

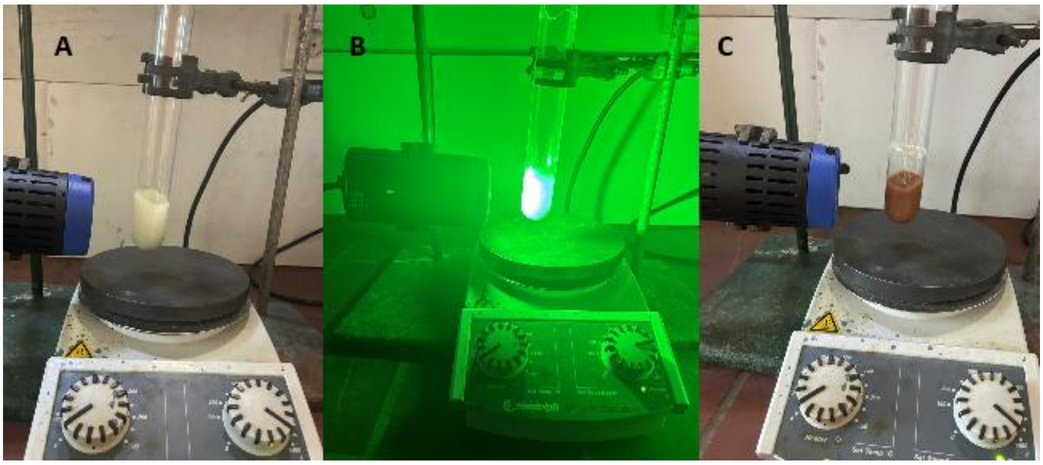

A. Reaction setup, the reaction mixture is placed 3 cm away from the irradiation source; **B.** Reaction mixture under LED lamps (Kessil PR 160L, 525 nm) irradiation; **C.** Reaction mixture upon reaction completion.

^{1}H-NMR of Phenol (2a) purified through extraction procedure.

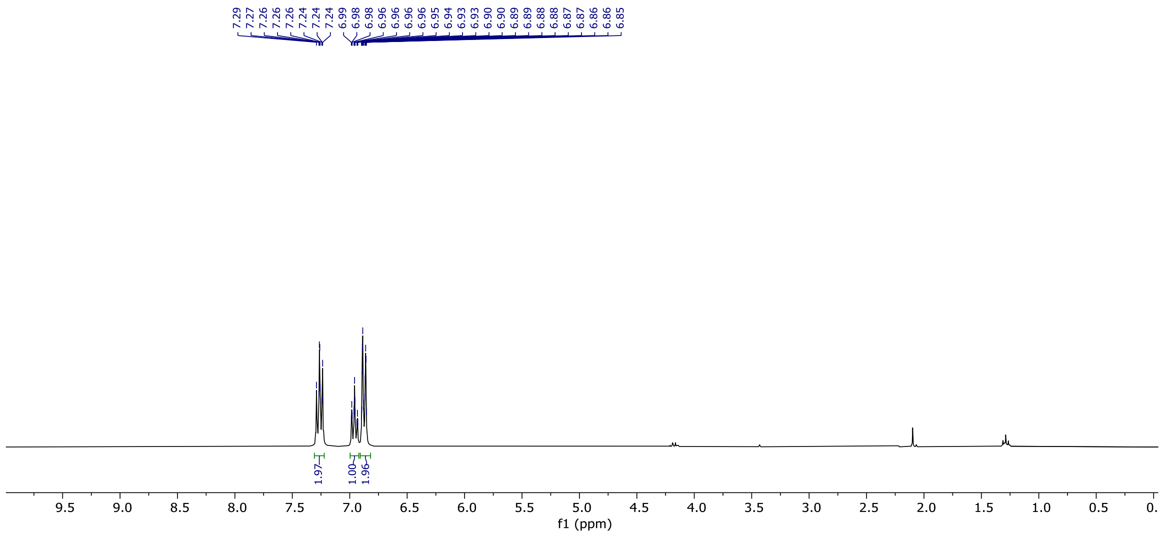

Procedure for g-C_3N_4 Recovery and Recycling

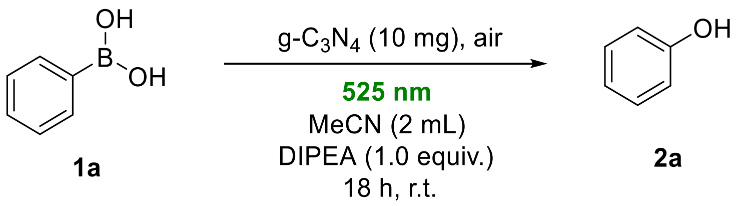

In a glass vial containing phenyl boronic acid (**1a**) (60 mg, 0.50 mmol), *N,N'*-diisopropylethylamine (65 mg, 0.50 mmol), g-C_3N_4 (10 mg) were added in acetonitrile (2.0 mL). The reaction mixture was left stirring under LED lamps (Kessil PR 160L, 525 nm) irradiation for 18 h. Upon reaction completion, the reaction mixture was filtered to recover the amount of g-C_3N_4. The solvent from the filtrate was removed *in vacuo* and the reaction yield was determined by column chromatography (Pet. Ether/AcOEt: 6:1).

The recovered g-C_3N_4 was charged in a glass vial containing phenyl boronic acid (**1a**) (60 mg, 0.50 mmol), *N,N'*-diisopropylethylamine (65 mg, 0.50 mmol) and acetonitrile (2.0 mL). The reaction mixture was left stirring under LED lamps (Kessil PR 160L, 525 nm) irradiation for 18 h. This procedure was repeated up to four consecutive reaction runs (in analogy), without appreciable loss of reactivity.

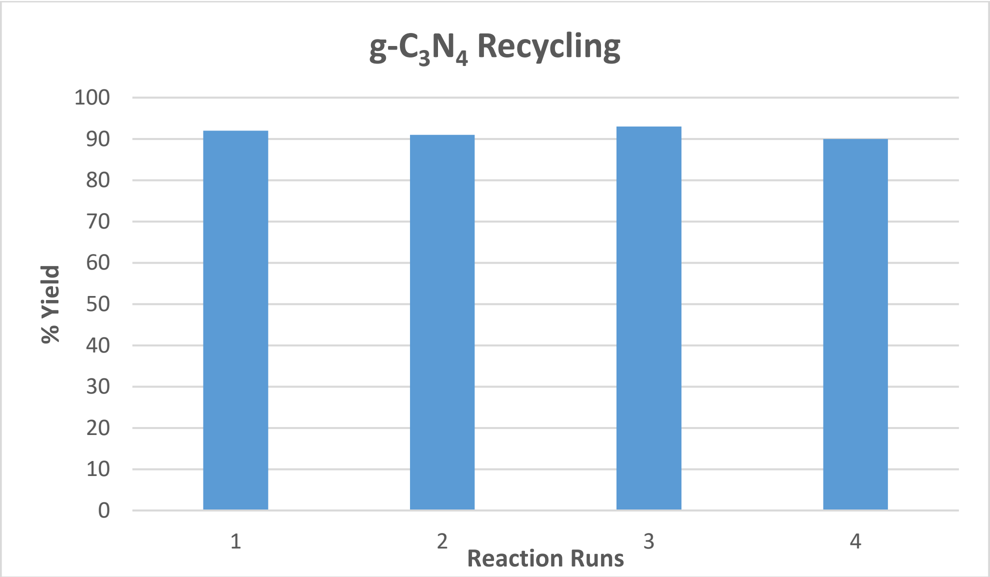

Quenching Studies on the Phenyl Boronic Acid Oxidation

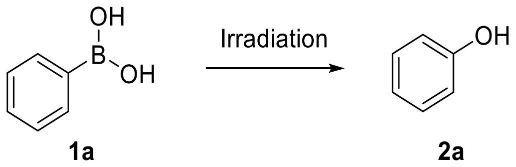

Quenchers / Source of Irradiation	Benzoquinone	NaN_3	TEMPO	BHT
525 nm[a]	0 %	0 %	0 %	0 %

[a] In a glass vial containing phenyl boronic acid (60 mg, 0.50 mmol), *N,N'*-diisopropylethylamine (65 mg, 0.50 mmol), quencher (1.0 equiv.) and g-C_3N_4 (10 mg) were added in acetonitrile (2 mL). The reaction mixture was left stirring under LED lamps (Kessil PR 160L, 525 nm) for 18 h. Conversions were determined by ^{1}H-NMR. All reactions were performed in triplicates, the average yield is provided.

Test for the detection of H_2O_2 production

A) Test for the detection of H_2O_2 from g-C_3N_4

In a glass vial g-C_3N_4 (10 mg) in MeCN (2.0 mL) was added. The reaction mixture was left stirring under LED lamps (Kessil PR 160L, 525 nm) irradiation for 18 h.

KI-glacial acetic acid test solution preparation: 20 mg of potassium iodide was dissolved in 2 mL of glacial acetic acid.

H_2O_2 generation test: The irradiated reaction mixture was added to the KI-glacial acetic acid test solution where immediate dark color appearance indicated the generation of H_2O_2 during the reaction.

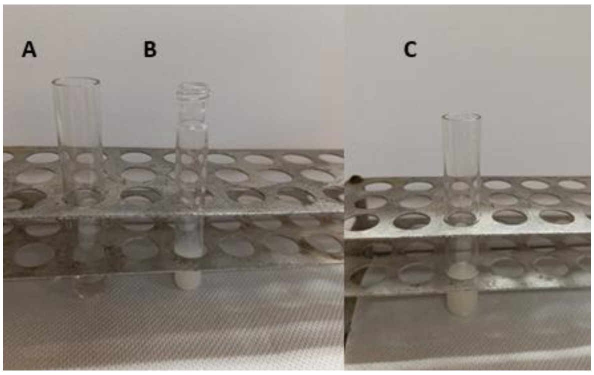

A) Reaction mixture after LED lamps (Kessil PR 160L, 525 nm) (18 h); **B)** KI-glacial acetic acid test solution; **C)** Solution resulting upon the addition of the irradiated reaction mixture to the KI-glacial acetic acid teat solution (***negative result***).

B) Test for the detection of H_2O_2 from the mixture of g-C_3N_4 with DIPEA

In a glass vial *N,N*'-diisopropylethylamine (65 mg, 0.50 mmol) and g-C_3N_4 (10 mg) in MeCN (2.0 mL) were added. The reaction mixture was left stirring under LED lamps (Kessil PR 160L, 525 nm) irradiation for 18 h.

KI-glacial acetic acid test solution preparation: 20 mg of potassium iodide was dissolved in 2 mL of glacial acetic acid.

H_2O_2 generation test: The irradiated reaction mixture was added to the KI-glacial acetic acid test solution where immediate dark color appearance indicated the generation of H_2O_2 during the reaction.

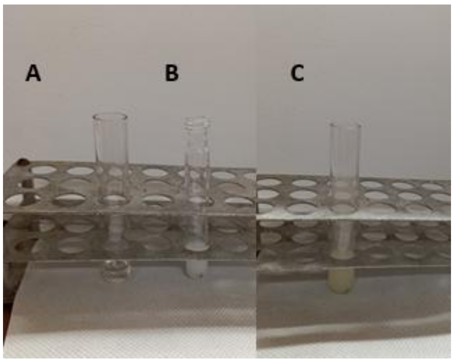

A) Reaction mixture after LED lamps (Kessil PR 160L, 525 nm) (18 h); **B)** KI-glacial acetic acid test solution; **C)** Solution resulting upon the addition of the irradiated reaction mixture to the KI-glacial acetic acid teat solution (***negative result***).

C) Test for the detection of H_2O_2 from the mixture of g-C_3N_4 with phenyl boronic acid

In a glass vial phenyl boronic acid (60 mg, 0.50 mmol) and g-C_3N_4 (10 mg) in MeCN (2.0 mL) were added. The reaction mixture was left stirring under LED lamps (Kessil PR 160L, 525 nm) irradiation for 18 h.

KI-glacial acetic acid test solution preparation: 20 mg of potassium iodide was dissolved in 2 mL of glacial acetic acid.

H_2O_2 generation test: The irradiated reaction mixture was added to the KI-glacial acetic acid test solution where immediate dark color appearance indicated the generation of H_2O_2 during the reaction.

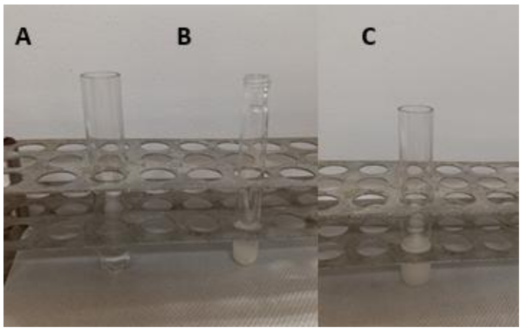

A) Reaction mixture after LED lamps (Kessil PR 160L, 525 nm) (18 h); **B)** KI-glacial acetic acid test solution; **C)** Solution resulting upon the addition of the irradiated reaction mixture to the KI-glacial acetic acid teat solution (***negative result***).

D) Test for the detection of H_2O_2 from the mixture of g-C_3N_4, DIPEA with phenyl boronic acid.

In a glass vial phenyl boronic acid (60 mg, 0.50 mmol), *N,N'* diisopropylethylamine (65 mg, 0.50 mmol) and g-C_3N_4 (10 mg) in MeCN (2.0 mL) were added. The reaction mixture was left stirring under LED lamps (Kessil PR 160L, 525 nm) irradiation for 18 h.

KI-glacial acetic acid test solution preparation: 20 mg of potassium iodide was dissolved in 2 mL of glacial acetic acid.

H_2O_2 generation test: The irradiated reaction mixture was added to the KI-glacial acetic acid test solution where immediate dark color appearance indicated the generation of H_2O_2 during the reaction.

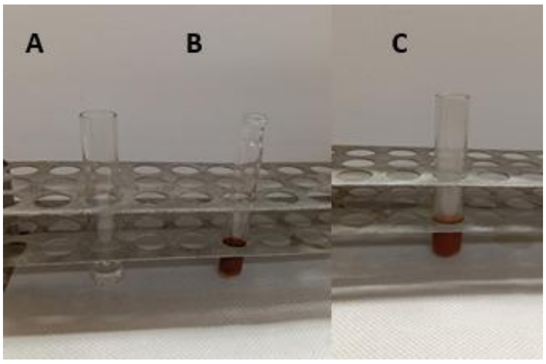

A) Reaction mixture after LED lamps (Kessil PR 160L, 525 nm) (18 h); **B)** KI-glacial acetic acid test solution; **C)** Solution resulting upon the addition of the irradiated reaction mixture to the KI-glacial acetic acid teat solution (***negative result***).

Determination of the Quantum Yield

Determination of the photon flux

A ferrioxalate actinometer solution was prepared following the Hammond variation of the Hatchard and Parker procedure[9] outlined in the *Handbook of Photochemistry*.[6] Ferrioxalate actinometer solution measures the decomposition of ferric ions to ferrous ions, which are complexed by 1,10-phenanthroline and monitored by UV/Vis absorbance at 510 nm. The moles of iron-phenanthroline complex formed are related to moles of photons absorbed. The values of the quantum yield of potassium ferrioxalate are related to the concentration and wavelength.

The solutions were prepared and stored under dark:

1. **Potassium ferrioxalate solution 0.012M**: 147.4 mg of potassium ferrioxalate and 69.5 μL of sulfuric acid (96%) were added to a 25 mL volumetric flask and filled to the mark with water (HPLC grade).

2. **Phenanthroline solution**: 0.2% by weight of 1,10-phenanthroline in water (100 mg in 50 mL volumetric flask or 50 mg in 25 mL).

3. **Buffer solution**: to a 100 mL volumetric flask 4.94 g of NaOAc and 1.0 mL of sulfuric acid (96%) were added and filled to the mark with water (HPLC grade).

Determination of the quantum yield for the photochemical reaction of phenylboronic acid **1a** at LED lamps (Kessil PR 160L, 525 nm).

In the case where LED lamps (Kessil PR 160L, 525 nm) were used as irradiation source, the photon flux was calculated as above and found to be 4.02 x 10^{-8} einstein s^{-1}.

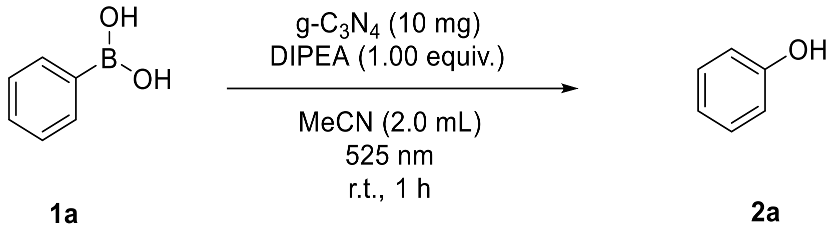

In a glass vial containing phenylboronic acid (**1a**) (60 mg, 0.50 mmol), *N,N'*-diisopropylethylamine (65 mg, 0.50 mmol) and and g-C_3N_4 (10 mg) in MeCN (2.0 mL) were added. The reaction mixture was left stirring under LED lamps (Kessil PR 160L, 525 nm) irradiation for 3600 s (1 h). After irradiation, the solvent was removed and the yield of the

product was determined by ^{1}H NMR (2%). The quantum yield was determined with the following equation:

$$\Phi = \frac{\text{mol product}}{\text{flux} \times \text{t} \times \text{f}} = \frac{0.005 \times 10^{-3}\ \text{mol}}{4.02 \times 10^{-8}\ \text{einstein s}^{-1} \times 3600\ \text{s} \times 0.3885} = 0.08$$

Mechanistic Investigation with UV-Vis

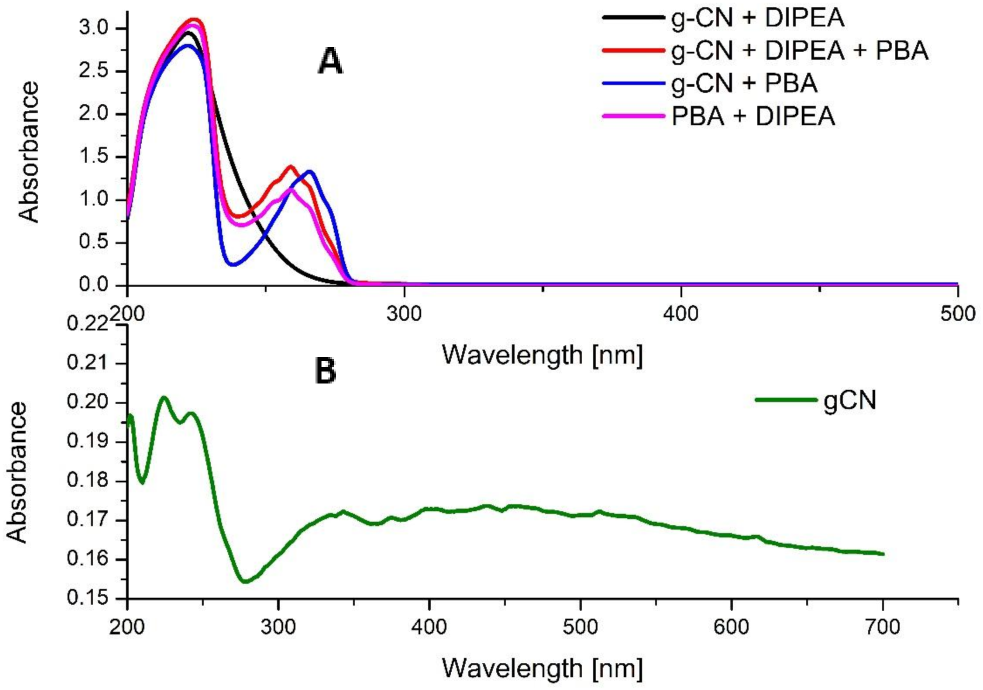

Figure S5 A. UV-Vis absorbance spectra of mixtures comprising g-C_3N_4 (1 mg), *N,N'*-diisopropylethylamine (10^{-3} M), phenyl boronic acid (**1a**) (10^{-3} M) in MeOH; **B.** UV-Vis absorbance spectra of g-C_3N_4 (1 mg) in MeOH.

References

1. Gkizis, P. L.; Constantinou, C. T.; Kokotos, C. G. *Eur. J. Org. Chem*., **2023**, *26*, e202300898.
2. K. Gennaiou, M. Petsi, B. Kakarikas, N. Iordanidis, A. L. Zografos, *Adv. Synth. Catal*. **2022**, *364*, 3059-3065.
3. H. Zhang, C. Zhou, Y. Zheng, X. Zhang, *Green Chem*. **2021**, *21*, 8878-8885.
4. S. K. Serviou, P. L. Gkizis, D. Puchan Sanchez, A. Soussana, A. H. G. David, C. Cabanetos, C. G. Kokotos, *Eur. J. Org. Chem.,* **2025**, *28*, e202500782.
5. H. Zhang, M. M. Conte, X.-C. Huang, Z. Khalil, R. J. Capon, *Org. Biomol. Chem*. **2012**, *10*, 2656-2663.
6. M. Montalti, A. Credi, L. Prodi, M. T. Gandolfi, *Handbook of Photochemistry*, Taylor Francis, **2006**.

NMR Spectra

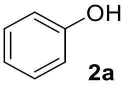

2a

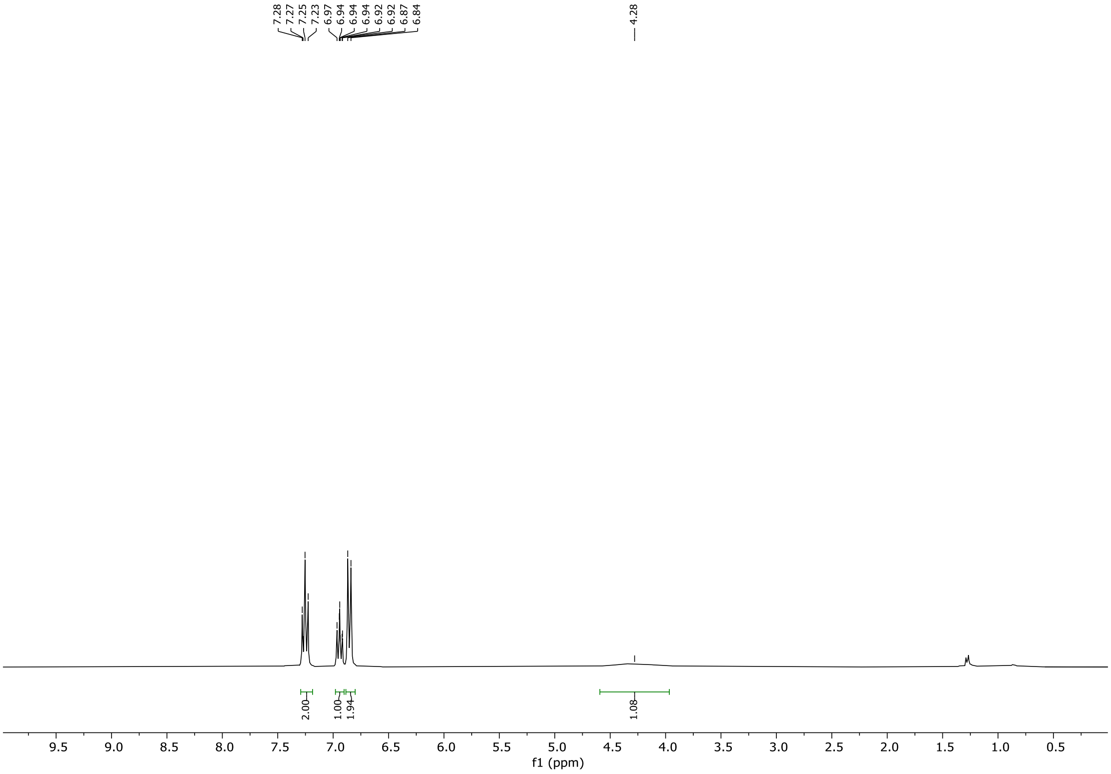

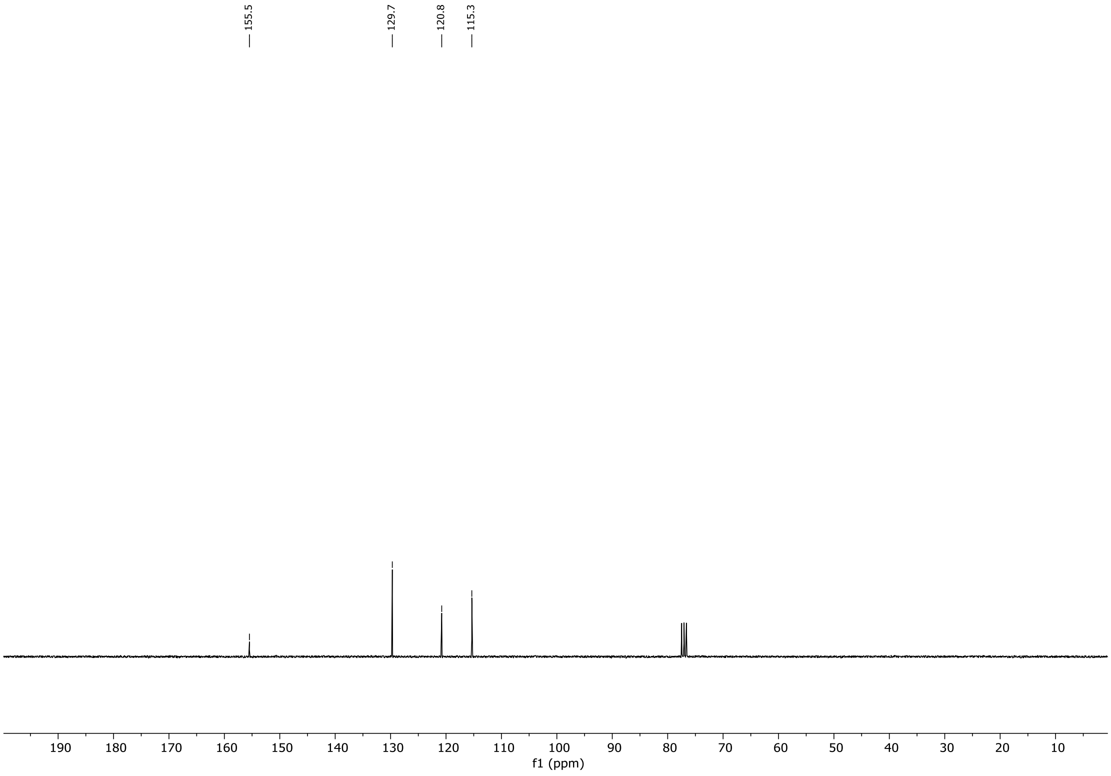

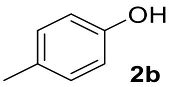
OH

2b

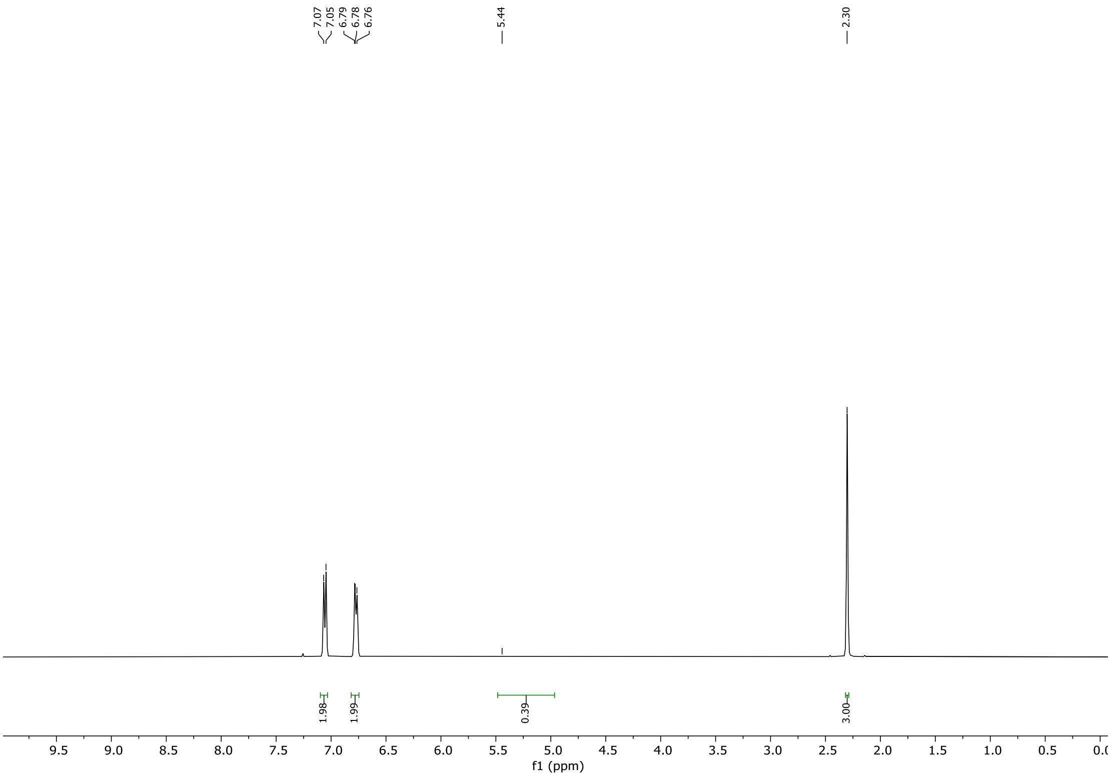
7.07
7.05
6.79
6.78
6.76
5.44
2.30
1.98
1.99
0.39
3.00
9.5 9.0 8.5 8.0 7.5 7.0 6.5 6.0 5.5 5.0 4.5 4.0 3.5 3.0 2.5 2.0 1.5 1.0 0.5
f1 (ppm)

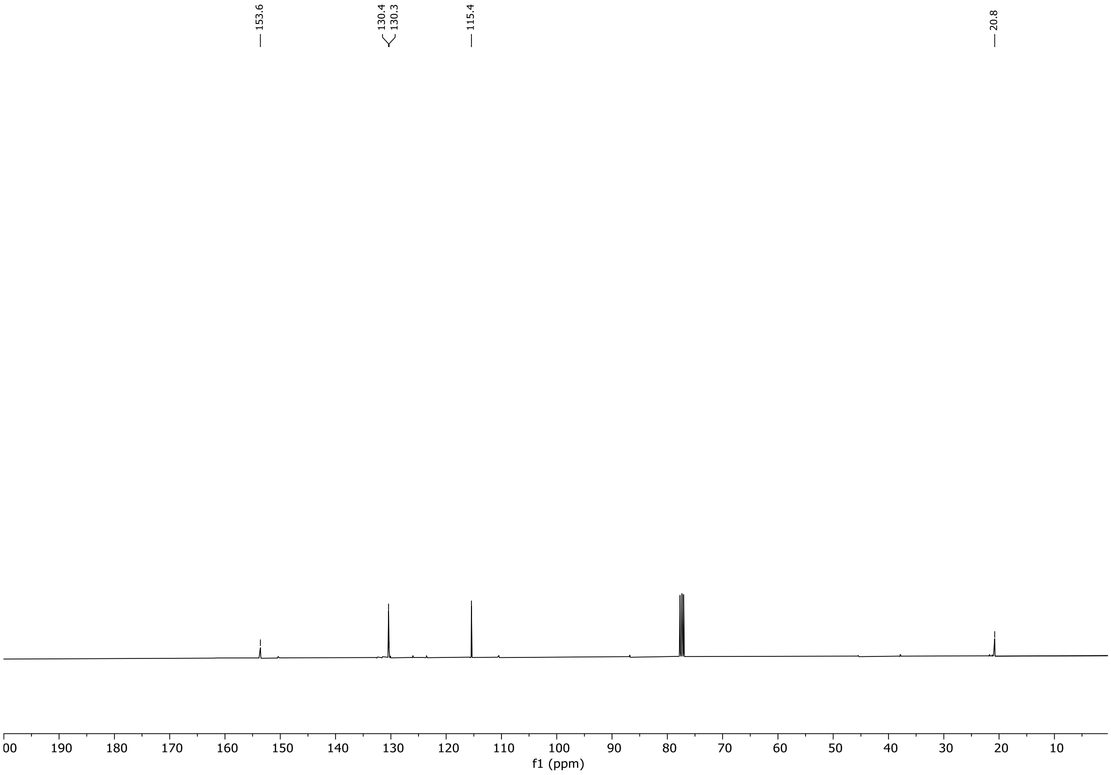
153.6
130.4
130.3
115.4
20.8
190 180 170 160 150 140 130 120 110 100 90 80 70 60 50 40 30 20 10
f1 (ppm)

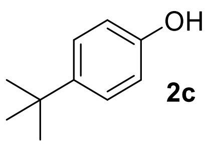
OH

2c

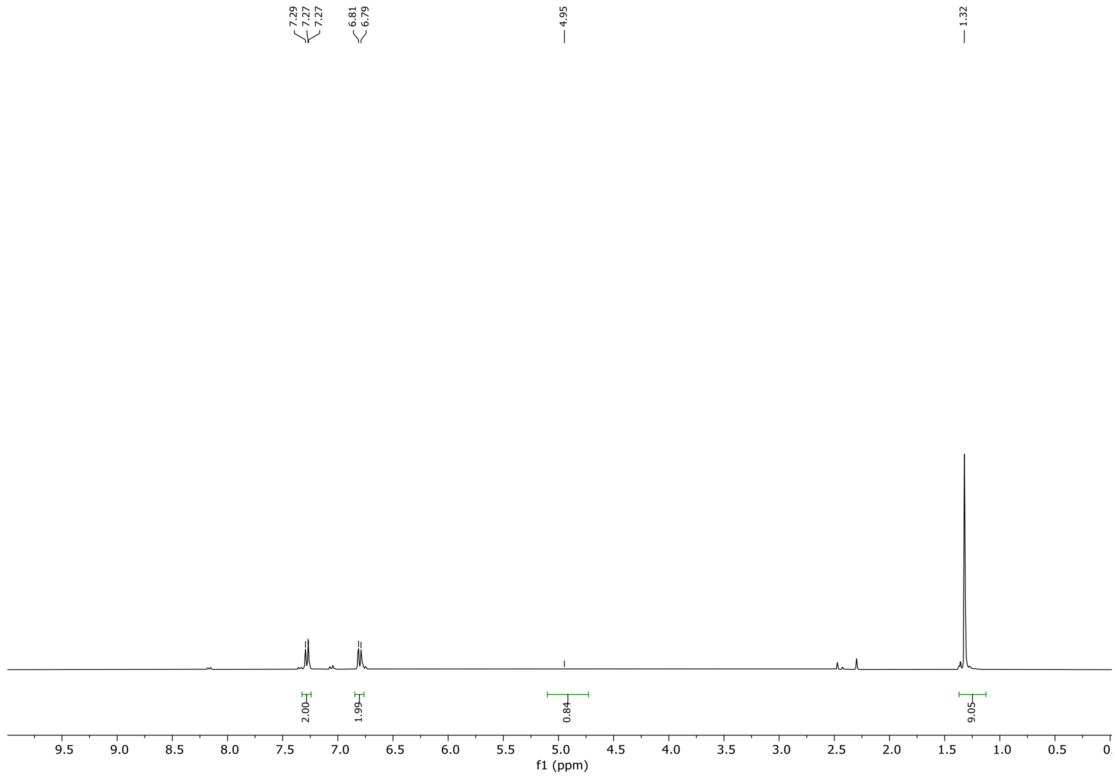
7.29
7.27
7.27
6.81
6.79
4.95
1.32
2.00
1.99
0.84
9.05
9.5 9.0 8.5 8.0 7.5 7.0 6.5 6.0 5.5 5.0 4.5 4.0 3.5 3.0 2.5 2.0 1.5 1.0 0.5
f1 (ppm)

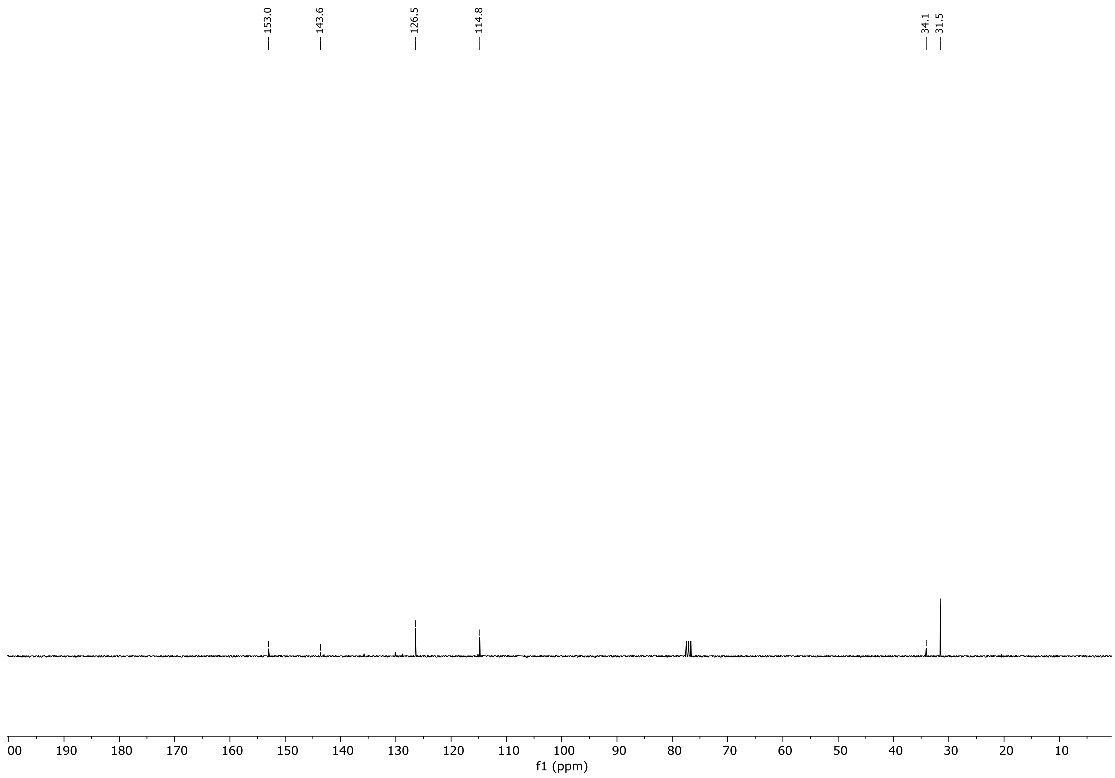
153.0
143.6
126.5
114.8
34.1
31.5
190 180 170 160 150 140 130 120 110 100 90 80 70 60 50 40 30 20 10
f1 (ppm)

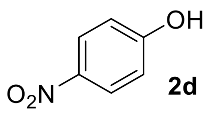
OH
O2N

2d

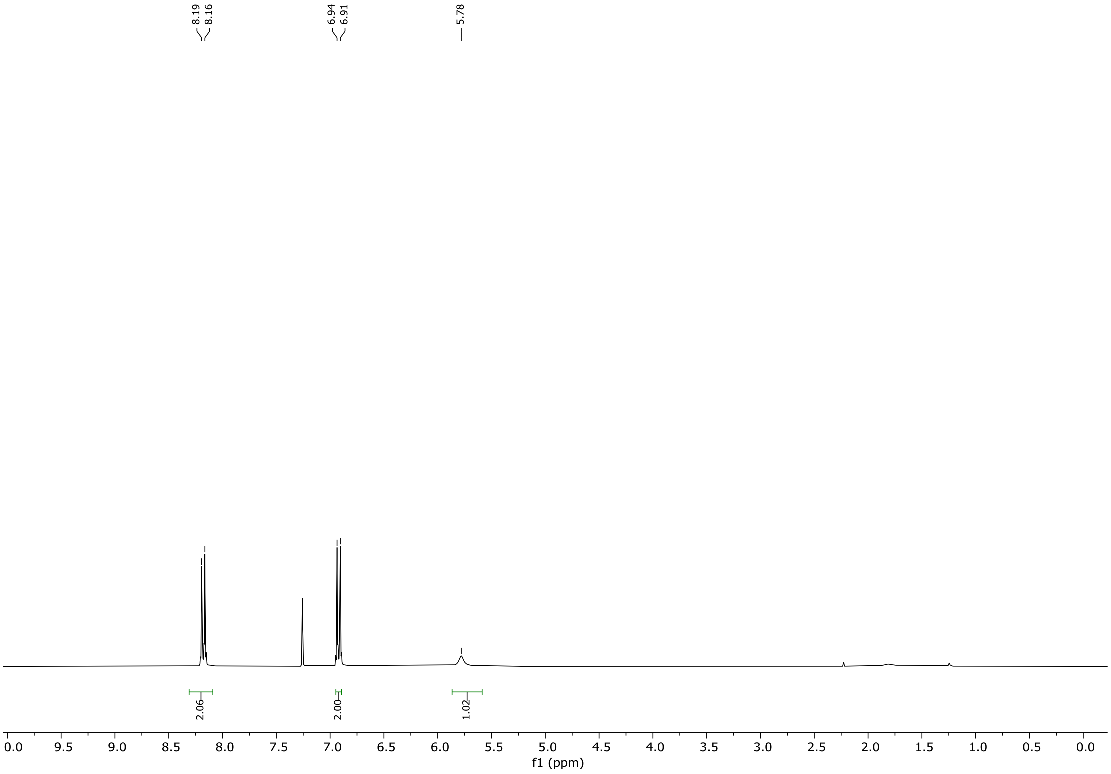
8.19
8.16
6.94
6.91
5.78
2.06
2.00
1.02
f1 (ppm)

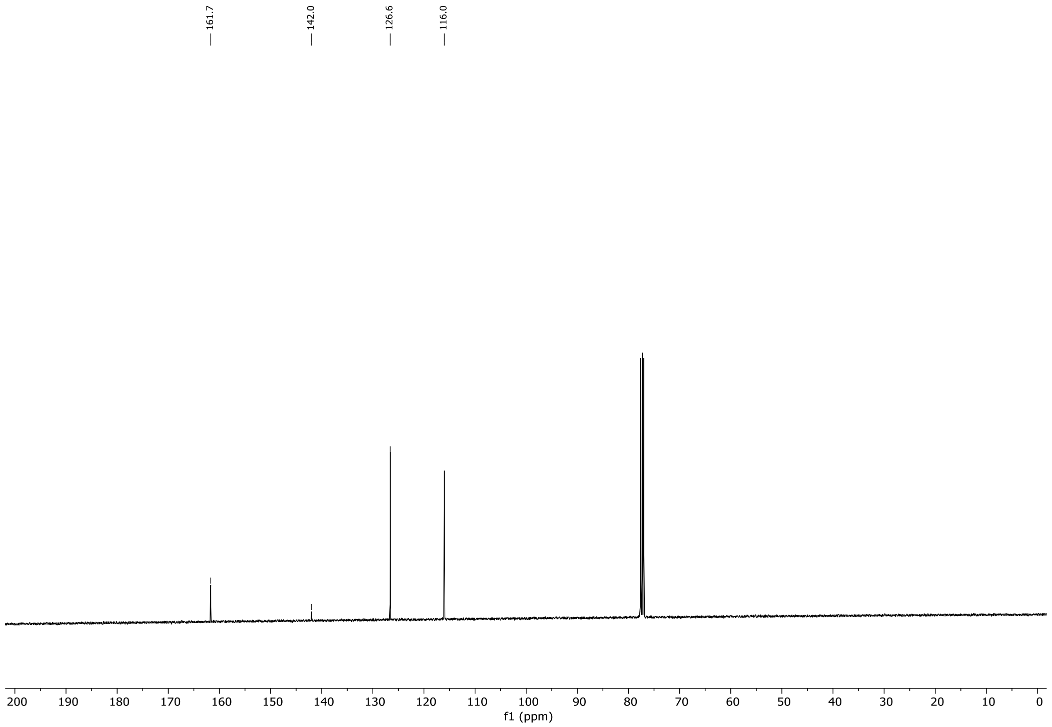
161.7
142.0
126.6
116.0
f1 (ppm)

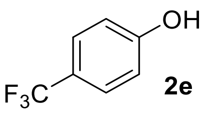
OH
F3C

2e

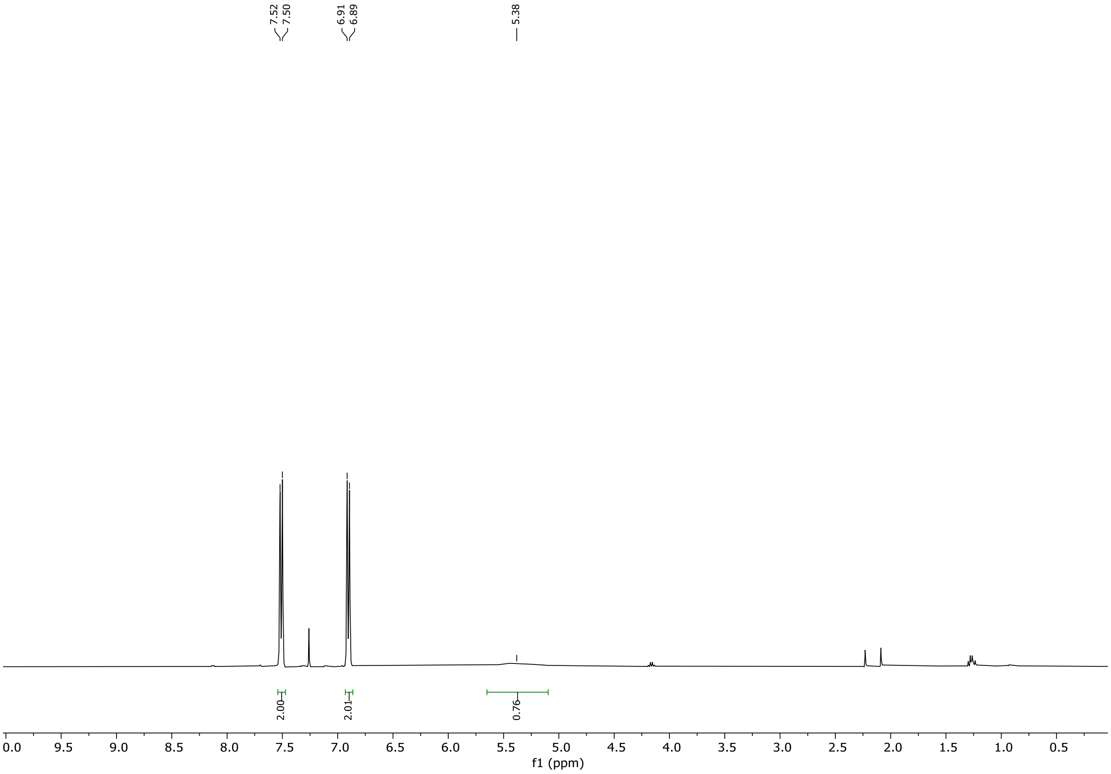
7.52
7.50
6.91
6.89
5.38
2.00
2.01
0.76
f1 (ppm)

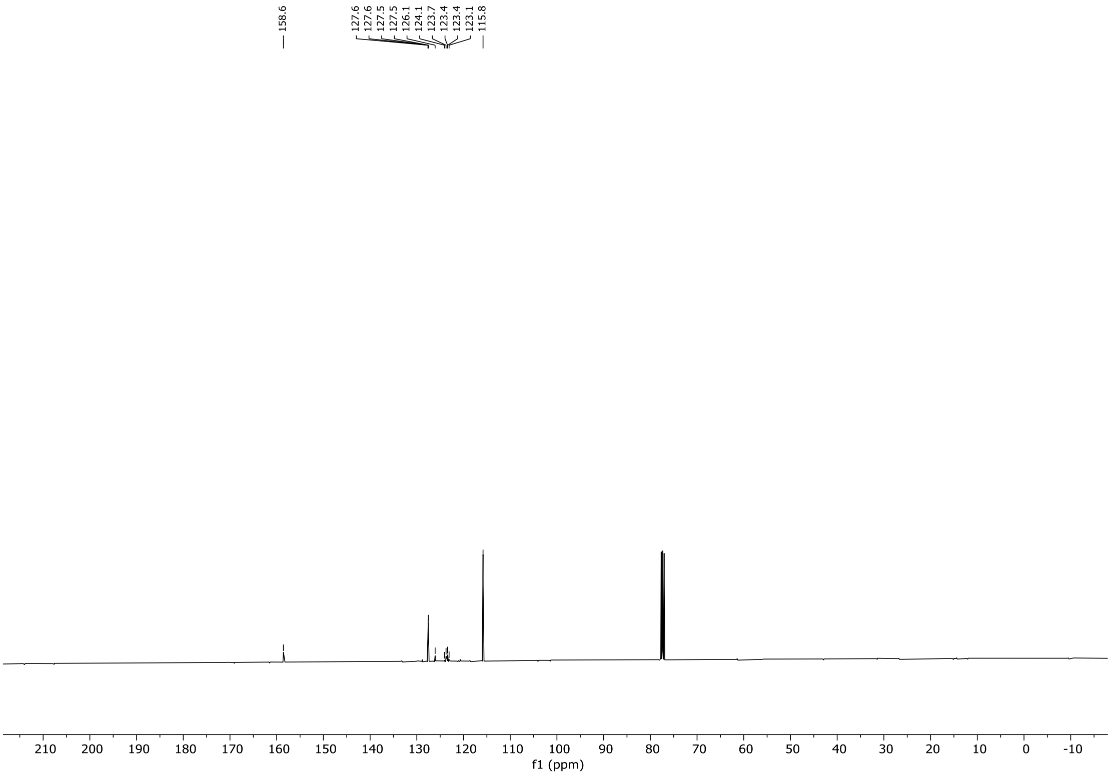
158.6
127.6
127.6
127.5
127.5
126.1
124.1
123.7
123.4
123.4
123.1
115.8
f1 (ppm)

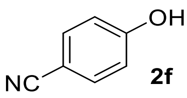
OH
NC

2f

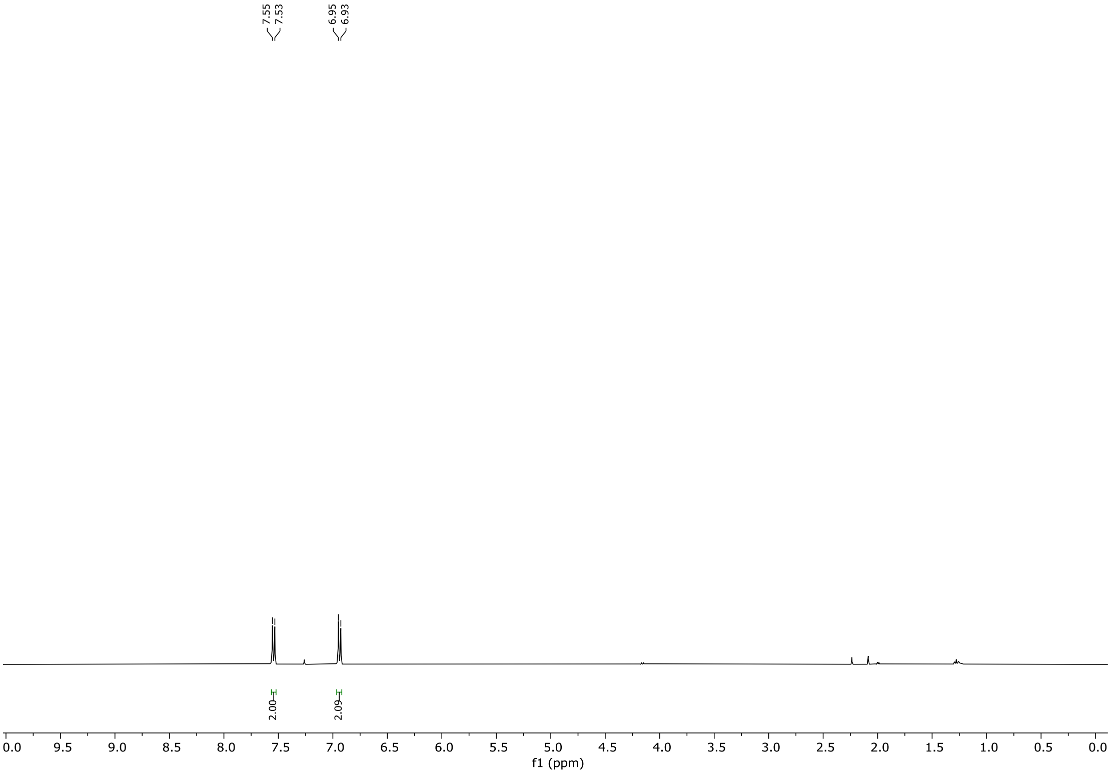
7.55
7.53
6.95
6.93
2.00
2.09
f1 (ppm)

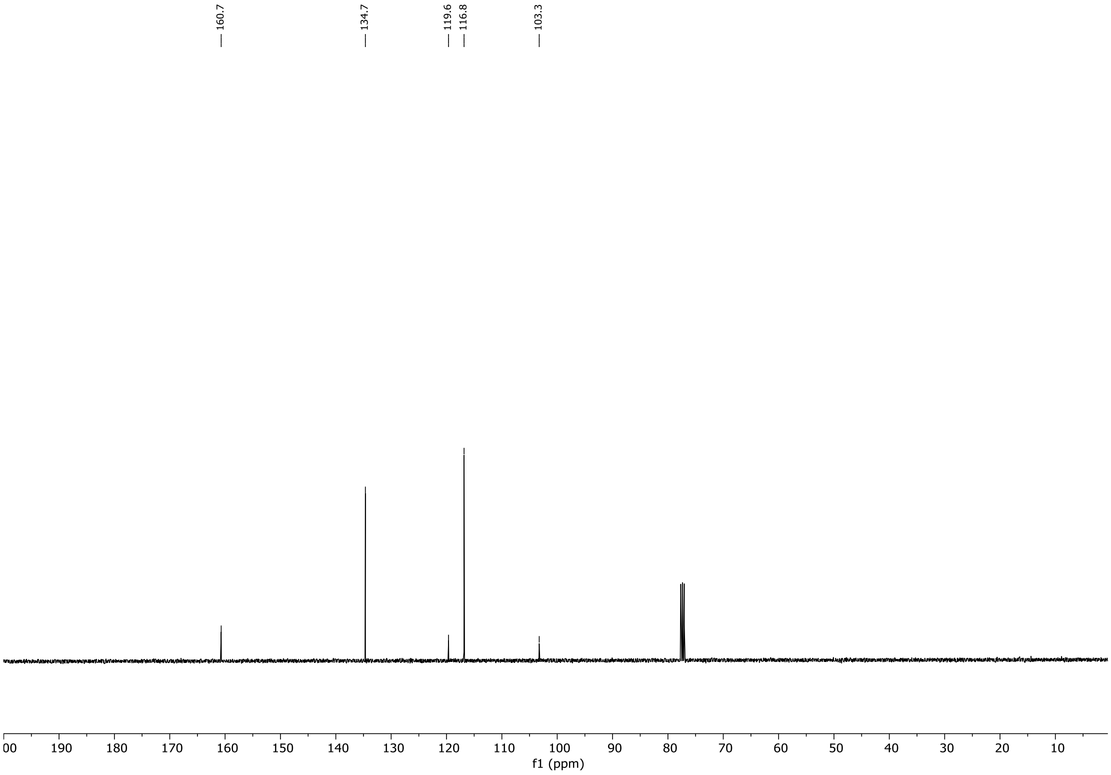
160.7
134.7
119.6
116.8
103.3
f1 (ppm)

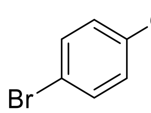
OH
Br

2g

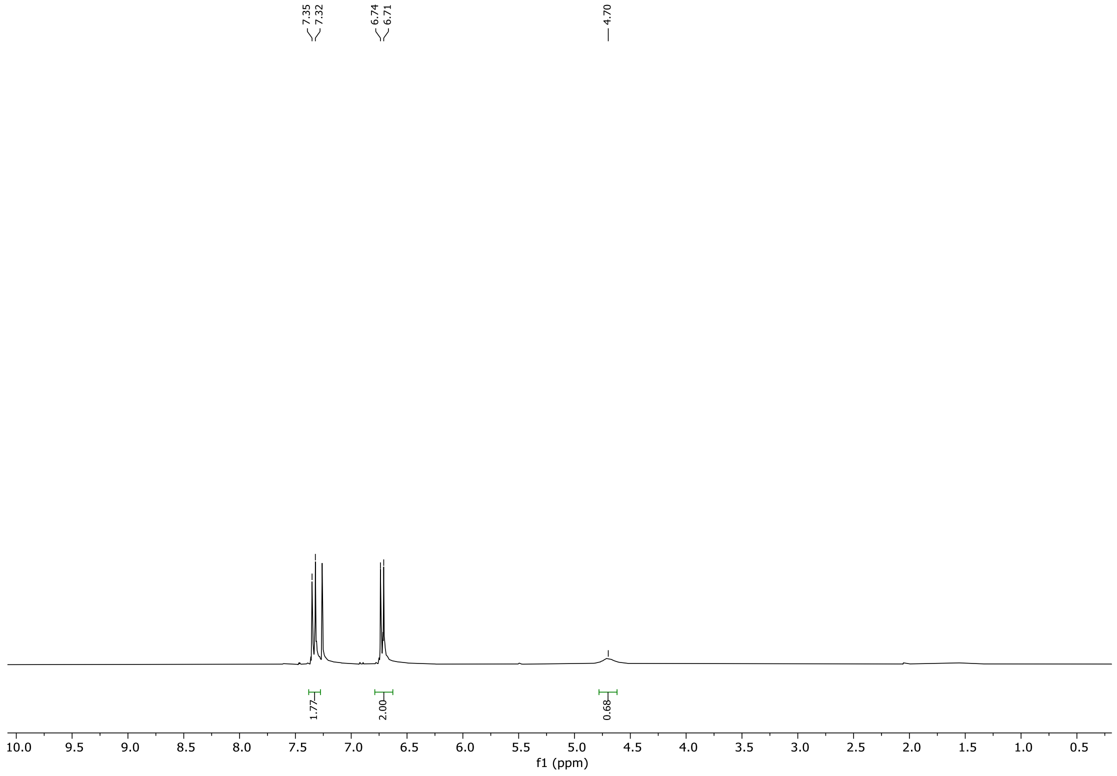
7.35
7.32
6.74
6.71
4.70
1.77
2.00
0.68
10.0 9.5 9.0 8.5 8.0 7.5 7.0 6.5 6.0 5.5 5.0 4.5 4.0 3.5 3.0 2.5 2.0 1.5 1.0 0.5
f1 (ppm)

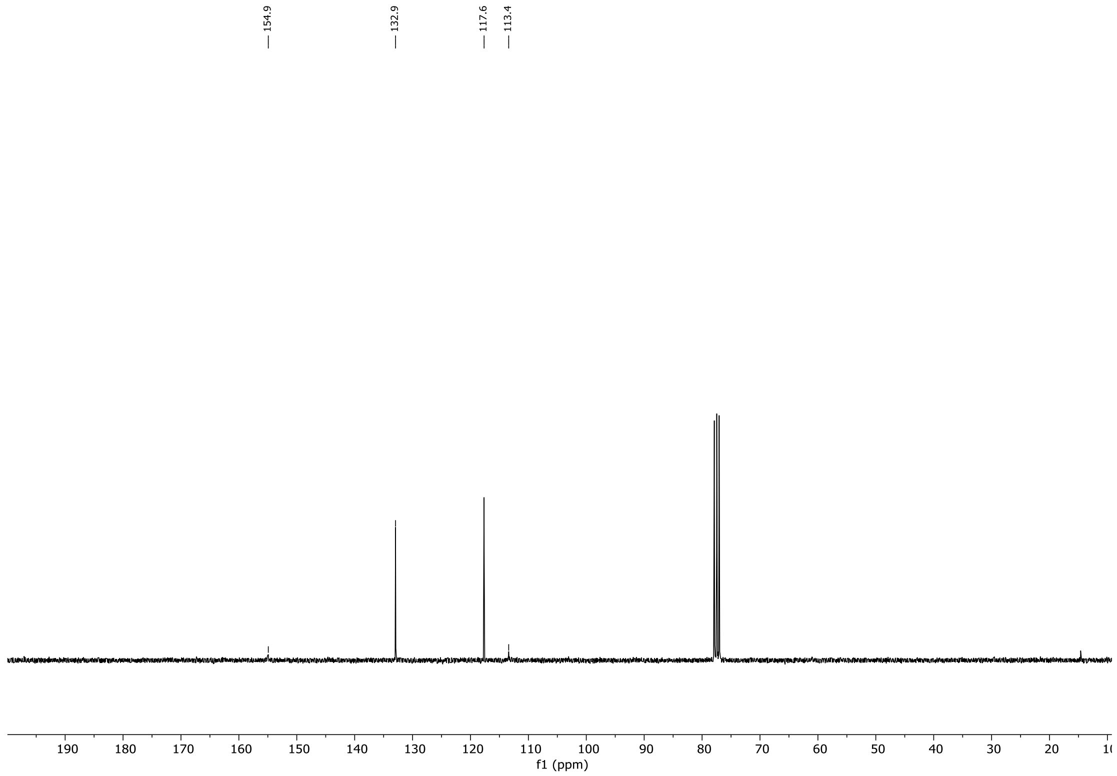
154.9
132.9
117.6
113.4
190 180 170 160 150 140 130 120 110 100 90 80 70 60 50 40 30 20
f1 (ppm)

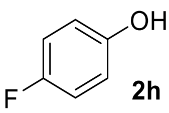
OH
F

2h

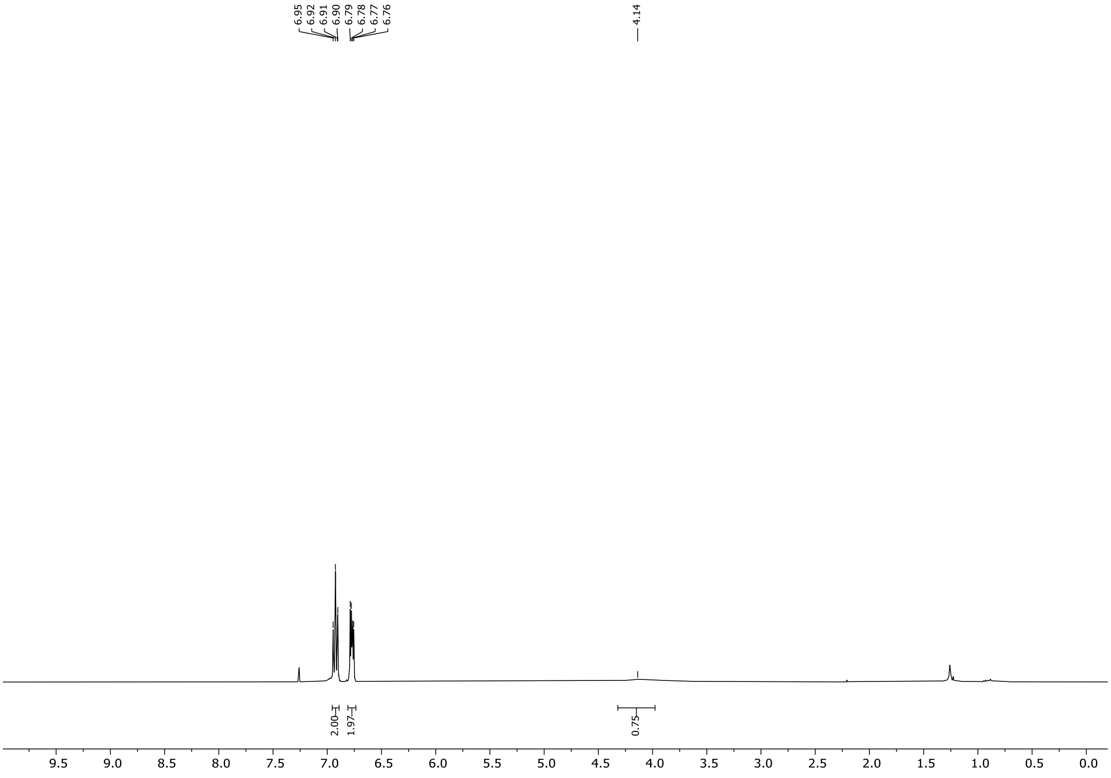
6.95
6.92
6.91
6.90
6.79
6.78
6.77
6.76
4.14
2.00
1.97
0.75
9.5 9.0 8.5 8.0 7.5 7.0 6.5 6.0 5.5 5.0 4.5 4.0 3.5 3.0 2.5 2.0 1.5 1.0 0.5 0.0

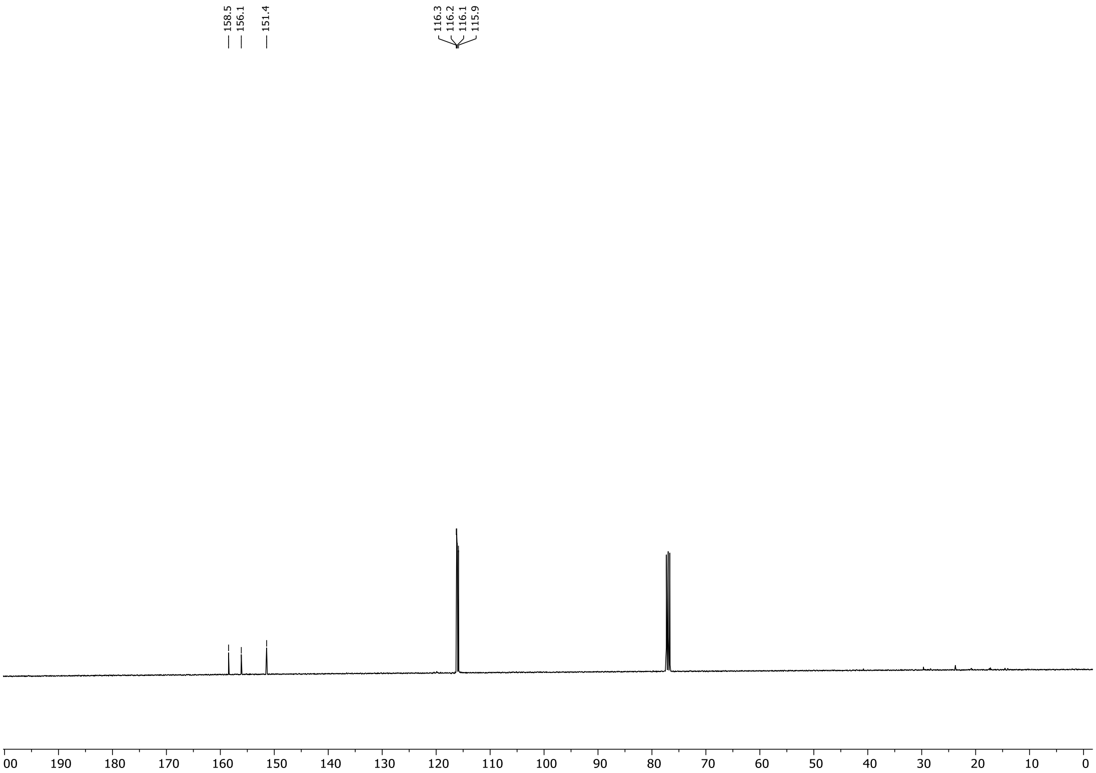
158.5
156.1
151.4
116.3
116.2
116.1
115.9
00 190 180 170 160 150 140 130 120 110 100 90 80 70 60 50 40 30 20 10 0

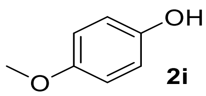

2i

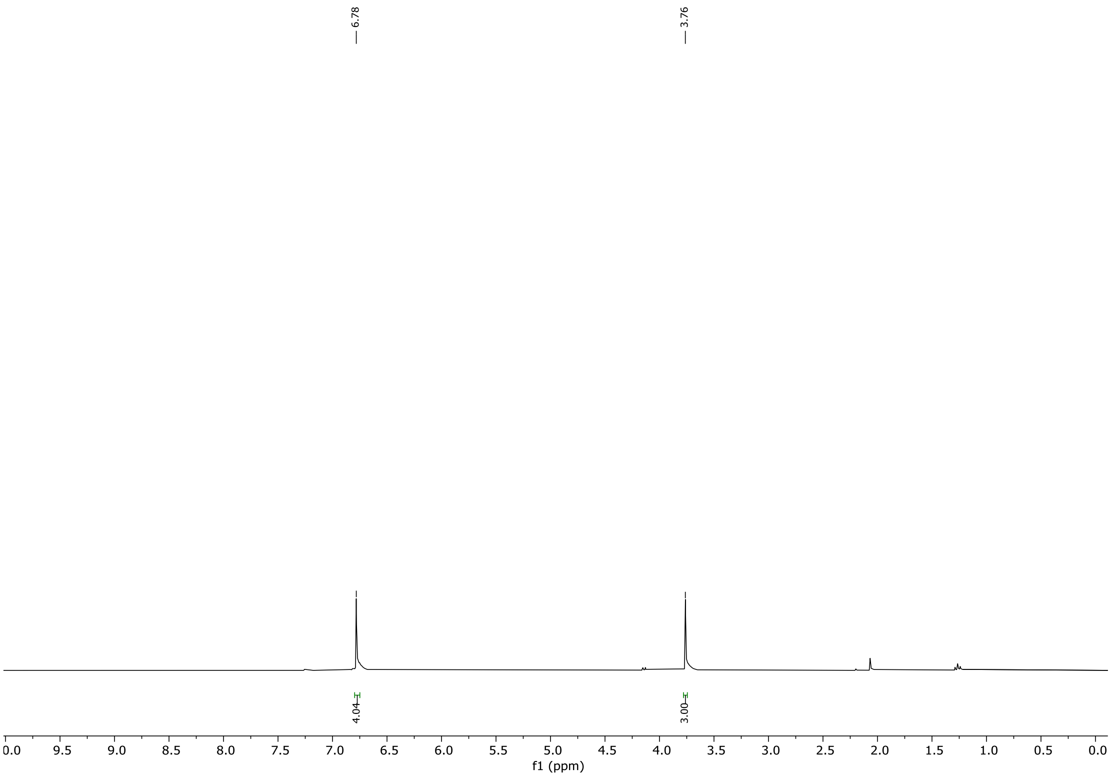

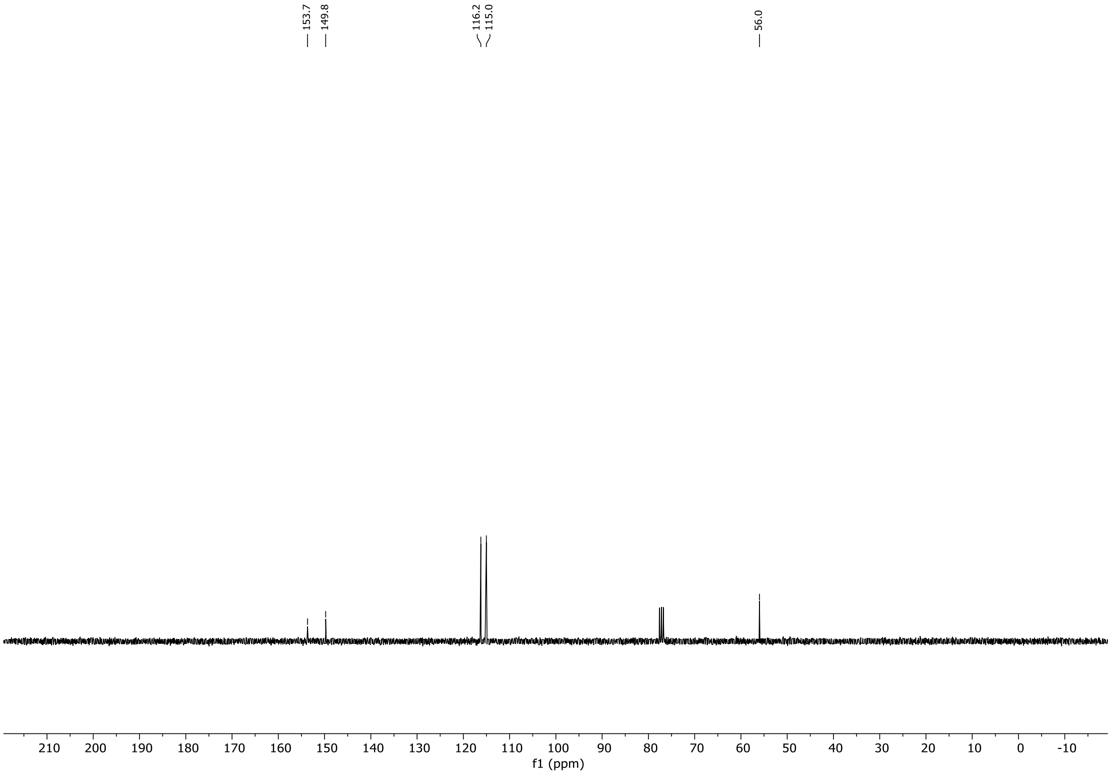

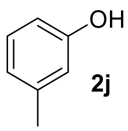
OH

2j

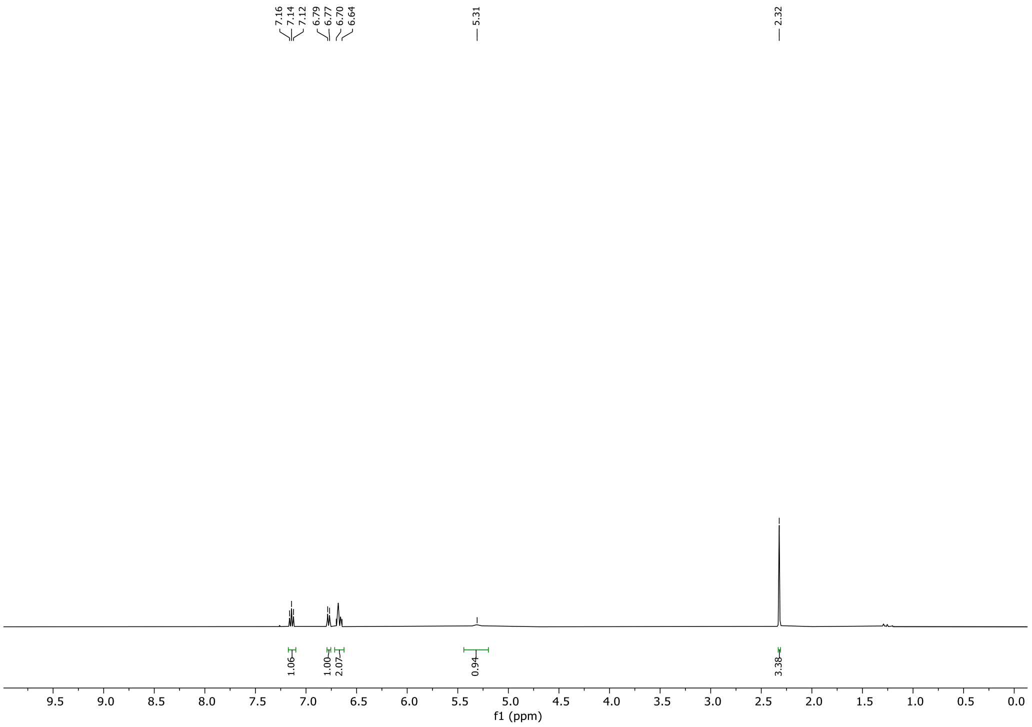
7.16
7.14
7.12
6.79
6.77
6.70
6.64
5.31
2.32
1.06
1.00
2.07
0.94
3.38
9.5 9.0 8.5 8.0 7.5 7.0 6.5 6.0 5.5 5.0 4.5 4.0 3.5 3.0 2.5 2.0 1.5 1.0 0.5 0.0
f1 (ppm)

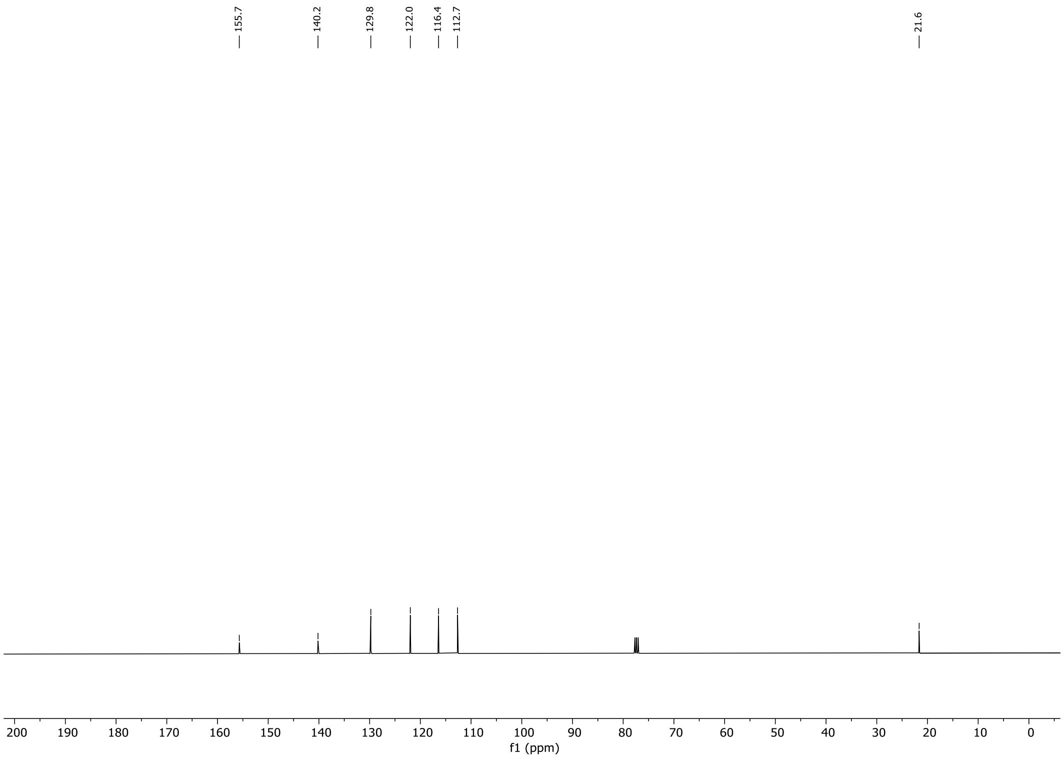
155.7
140.2
129.8
122.0
116.4
112.7
21.6
200 190 180 170 160 150 140 130 120 110 100 90 80 70 60 50 40 30 20 10 0
f1 (ppm)

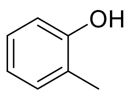
OH

2k

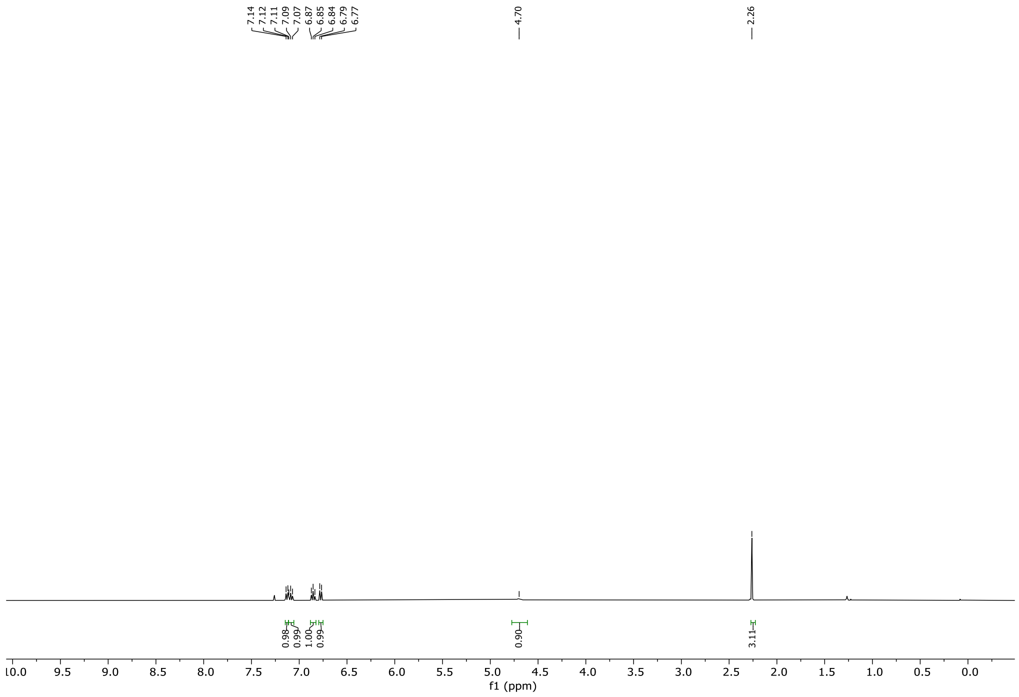
7.14
7.12
7.11
7.09
7.07
6.87
6.85
6.84
6.79
6.77
4.70
2.26
0.98
0.99
1.00
0.99
0.90
3.11
f1 (ppm)

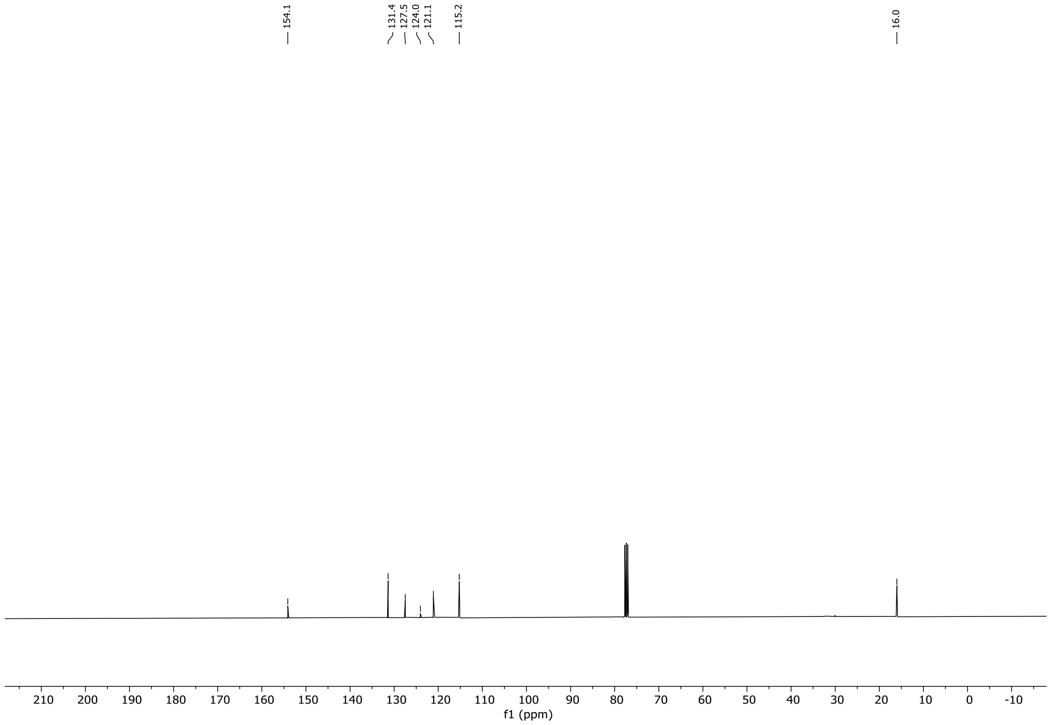
154.1
131.4
127.5
124.0
121.1
115.2
16.0
f1 (ppm)

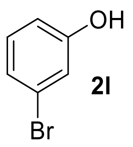
OH
Br

2l

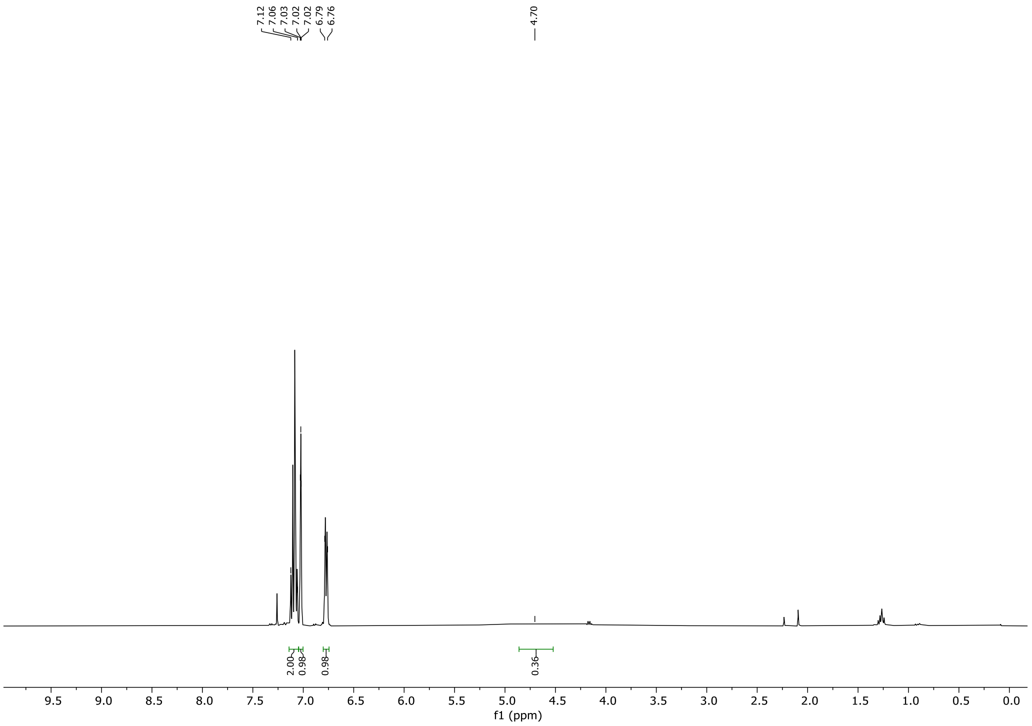
7.12
7.06
7.03
7.02
7.02
6.79
6.76
4.70
2.00
0.98
0.98
0.36
9.5 9.0 8.5 8.0 7.5 7.0 6.5 6.0 5.5 5.0 4.5 4.0 3.5 3.0 2.5 2.0 1.5 1.0 0.5 0.0
f1 (ppm)

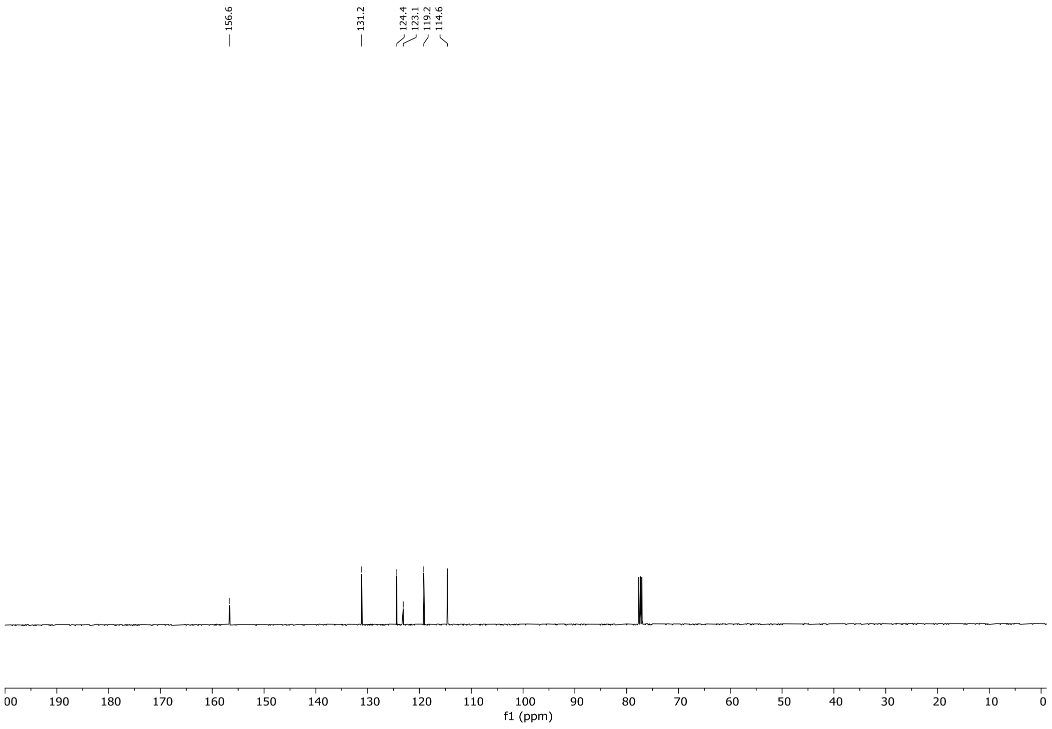
156.6
131.2
124.4
123.1
119.2
114.6
00 190 180 170 160 150 140 130 120 110 100 90 80 70 60 50 40 30 20 10 0
f1 (ppm)

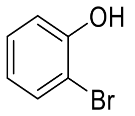
OH
Br

2m

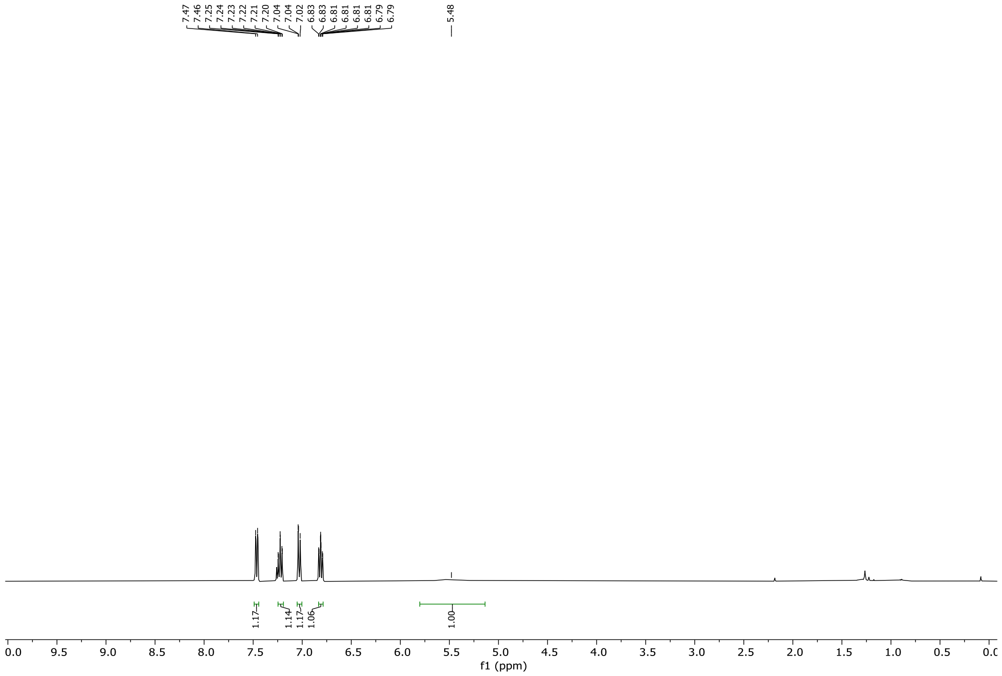
7.47
7.46
7.25
7.24
7.23
7.22
7.21
7.20
7.04
7.04
7.02
6.83
6.83
6.81
6.81
6.81
6.81
6.79
6.79
5.48
1.17
1.14
1.17
1.06
1.00
f1 (ppm)

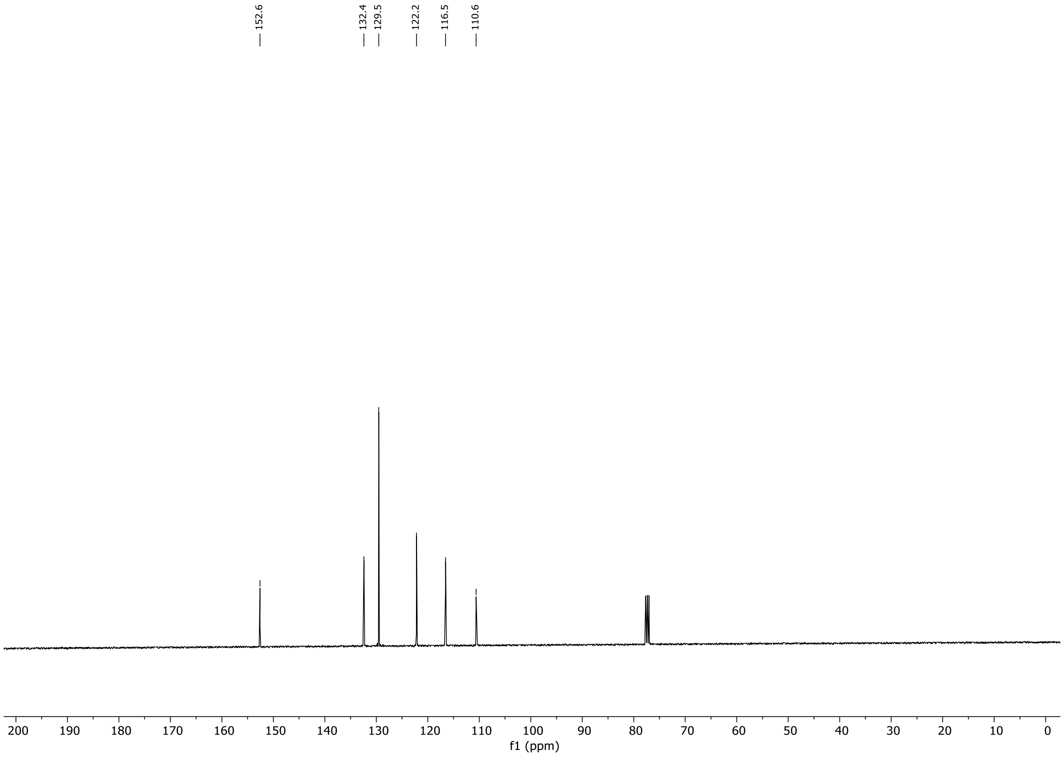
152.6
132.4
129.5
122.2
116.5
110.6
f1 (ppm)

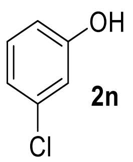
OH
Cl

2n

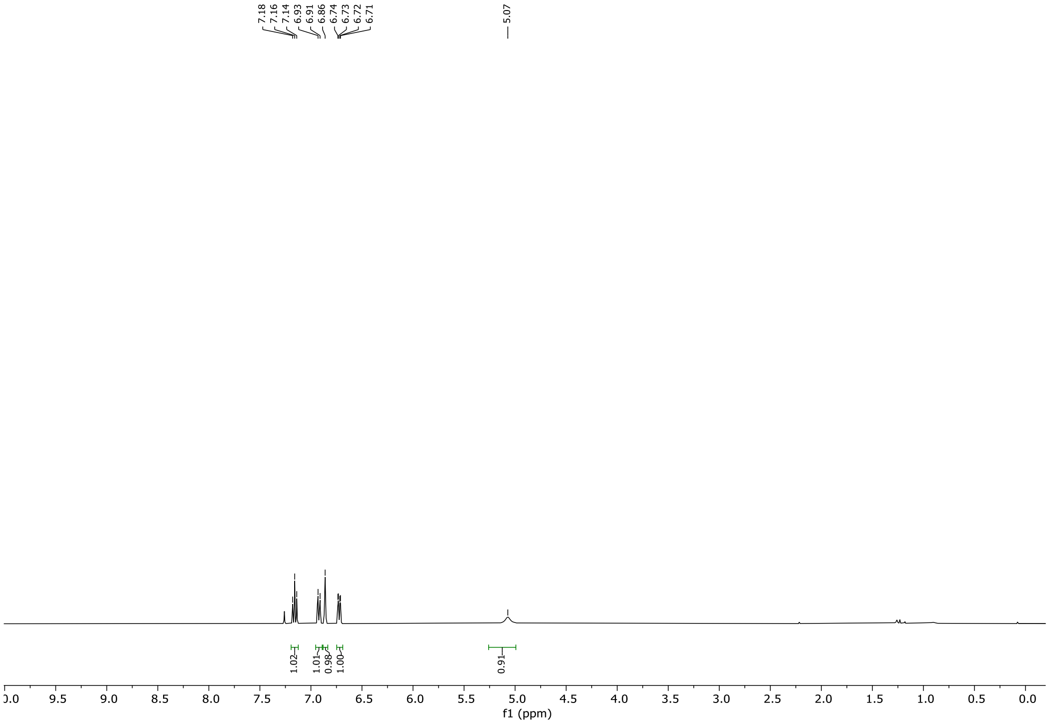
7.18
7.16
7.14
6.93
6.91
6.86
6.74
6.73
6.72
6.71
5.07
1.02
1.01
0.98
1.00
0.91
f1 (ppm)

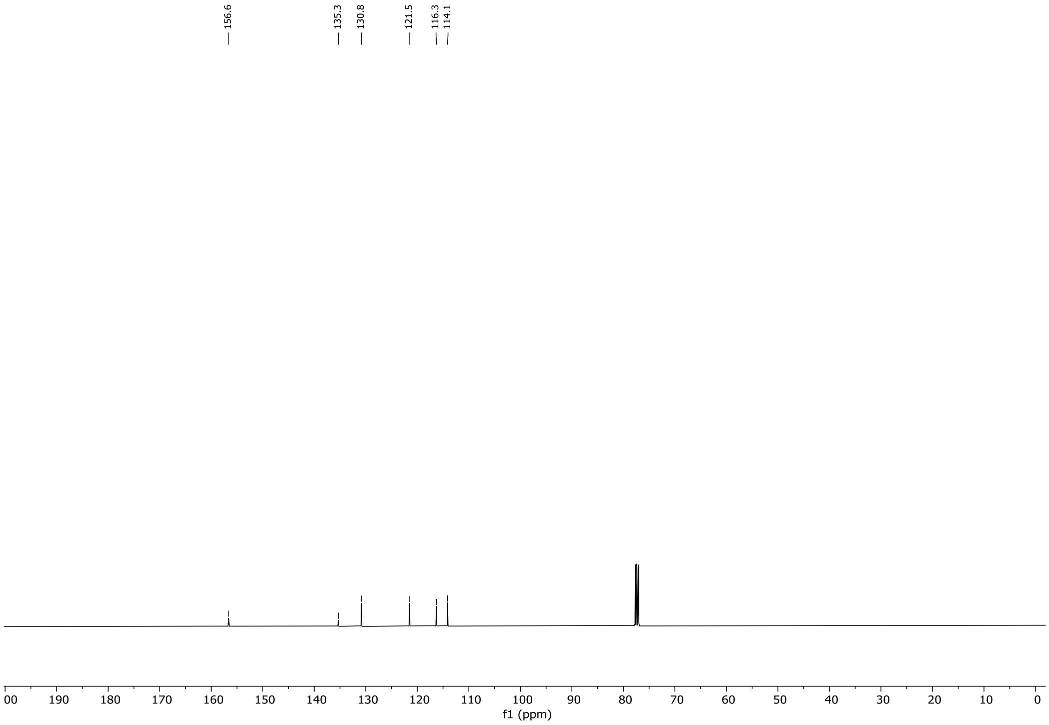
156.6
135.3
130.8
121.5
116.3
114.1
f1 (ppm)

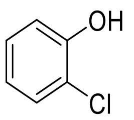

2o

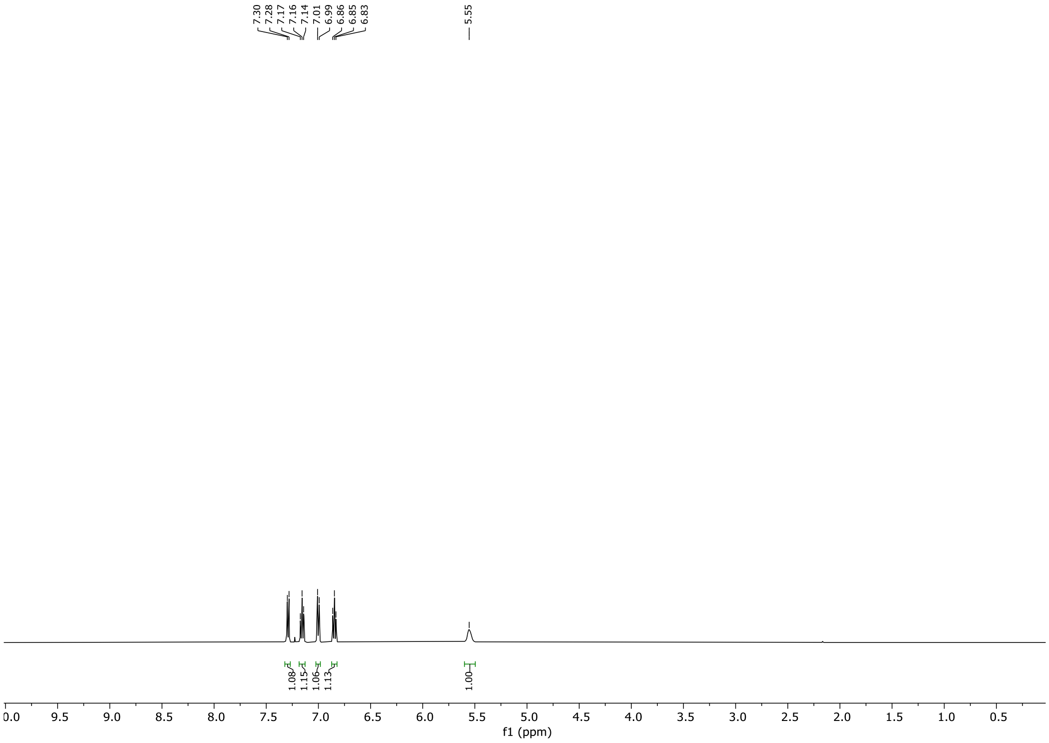
7.30
7.28
7.17
7.16
7.14
7.01
6.99
6.86
6.85
6.83
5.55
1.08
1.15
1.06
1.13
1.00
10.0 9.5 9.0 8.5 8.0 7.5 7.0 6.5 6.0 5.5 5.0 4.5 4.0 3.5 3.0 2.5 2.0 1.5 1.0 0.5
f1 (ppm)

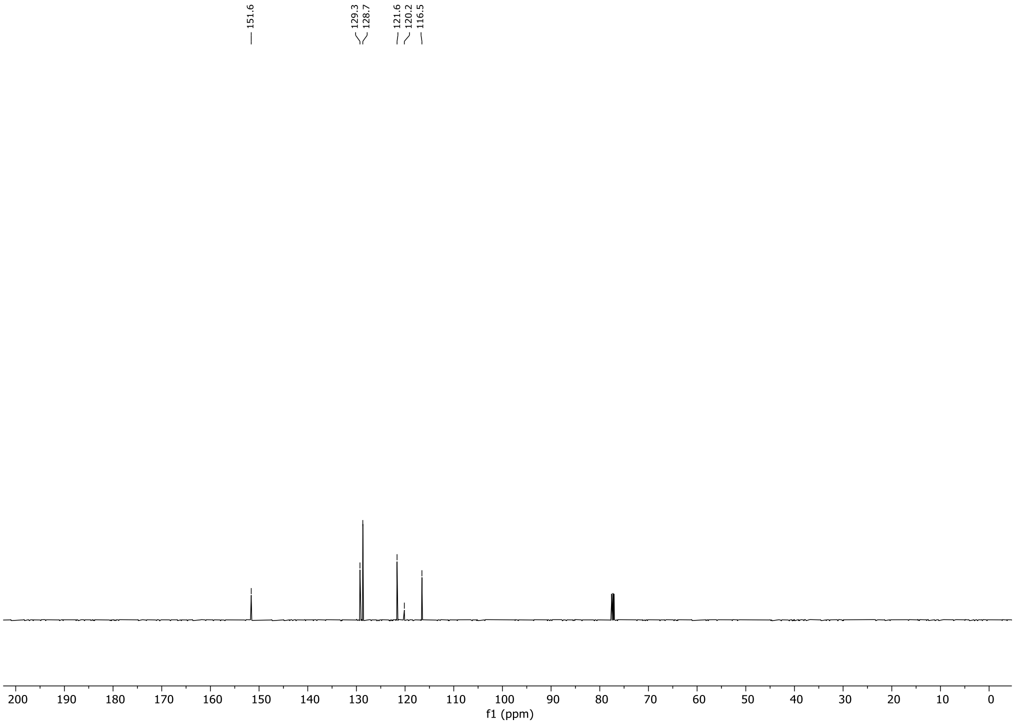
151.6
129.3
128.7
121.6
120.2
116.5
200 190 180 170 160 150 140 130 120 110 100 90 80 70 60 50 40 30 20 10 0
f1 (ppm)

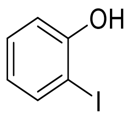
OH
I

2p

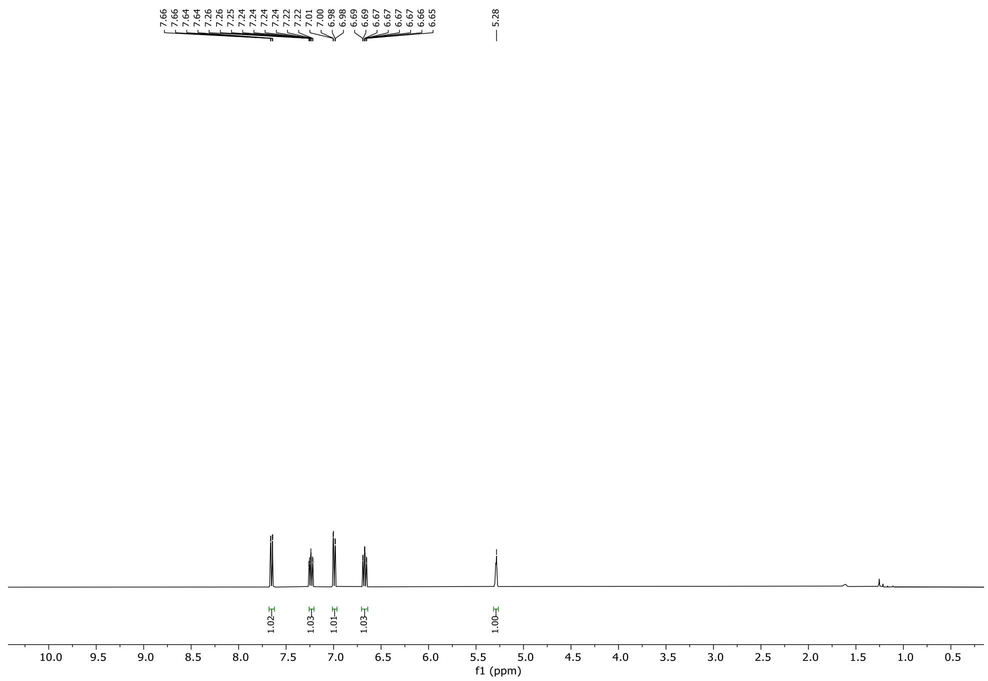
7.66
7.66
7.64
7.64
7.26
7.26
7.25
7.24
7.24
7.24
7.24
7.22
7.22
7.01
7.00
6.98
6.98
6.69
6.69
6.67
6.67
6.67
6.66
6.65
5.28
1.02
1.03
1.01
1.03
1.00
10.0 9.5 9.0 8.5 8.0 7.5 7.0 6.5 6.0 5.5 5.0 4.5 4.0 3.5 3.0 2.5 2.0 1.5 1.0 0.5
f1 (ppm)

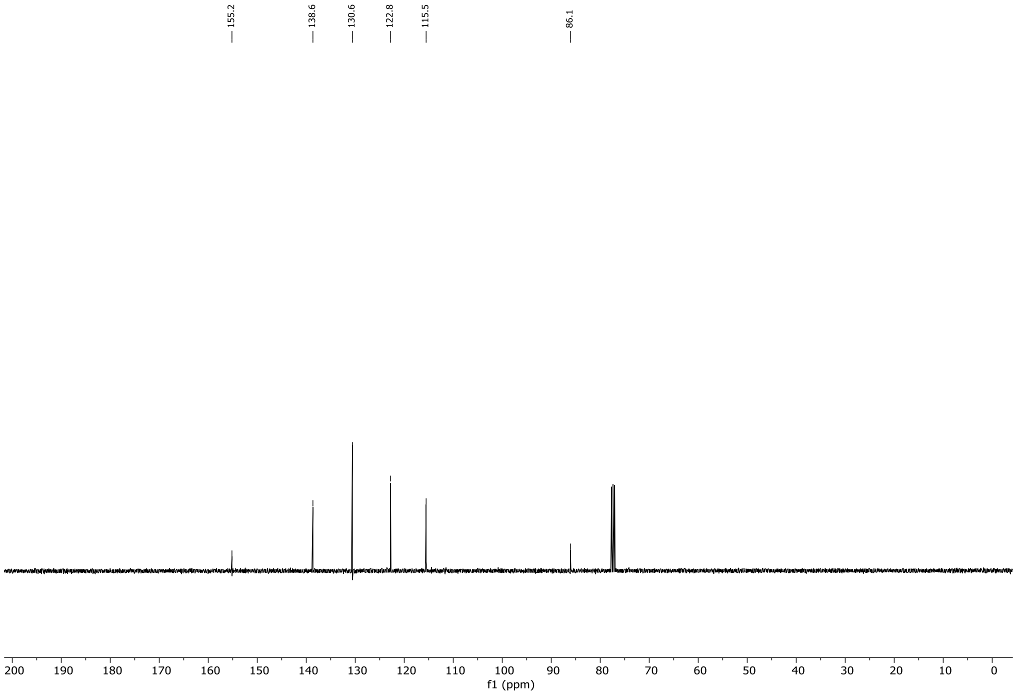
155.2
138.6
130.6
122.8
115.5
86.1
200 190 180 170 160 150 140 130 120 110 100 90 80 70 60 50 40 30 20 10 0
f1 (ppm)

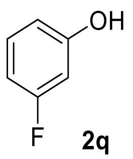
OH
F

2q

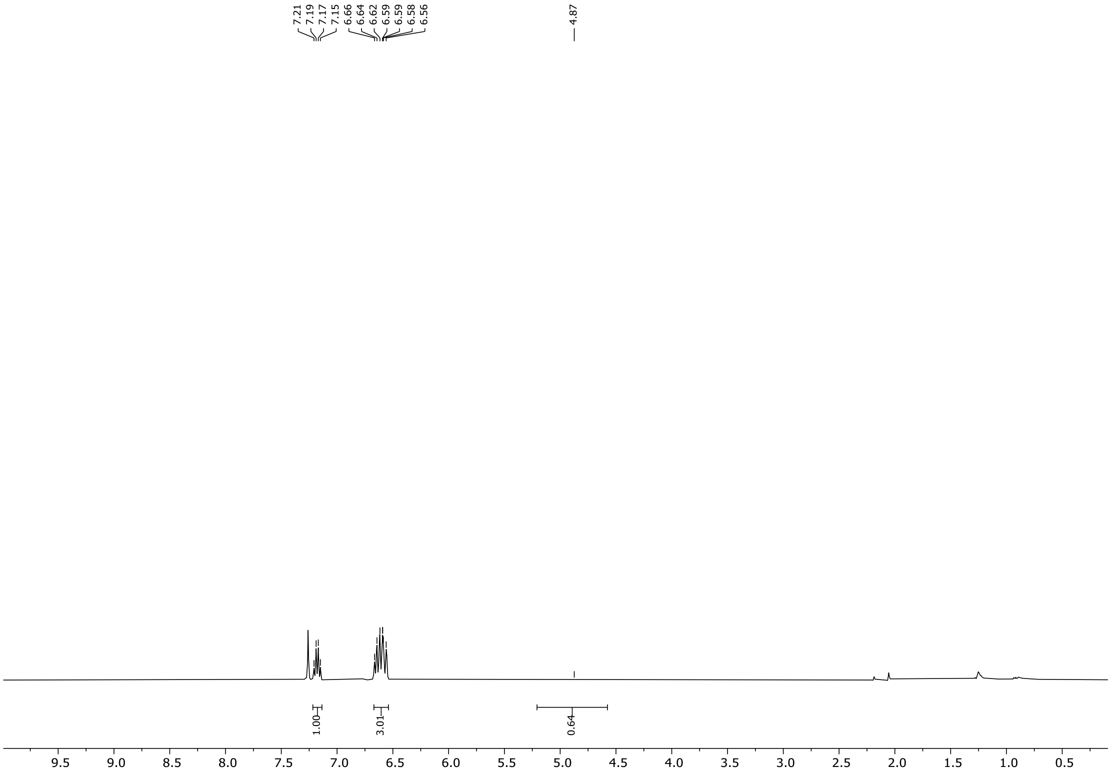
7.21
7.19
7.17
7.15
6.66
6.64
6.62
6.59
6.59
6.58
6.56
4.87
1.00
3.01
0.64
9.5 9.0 8.5 8.0 7.5 7.0 6.5 6.0 5.5 5.0 4.5 4.0 3.5 3.0 2.5 2.0 1.5 1.0 0.5

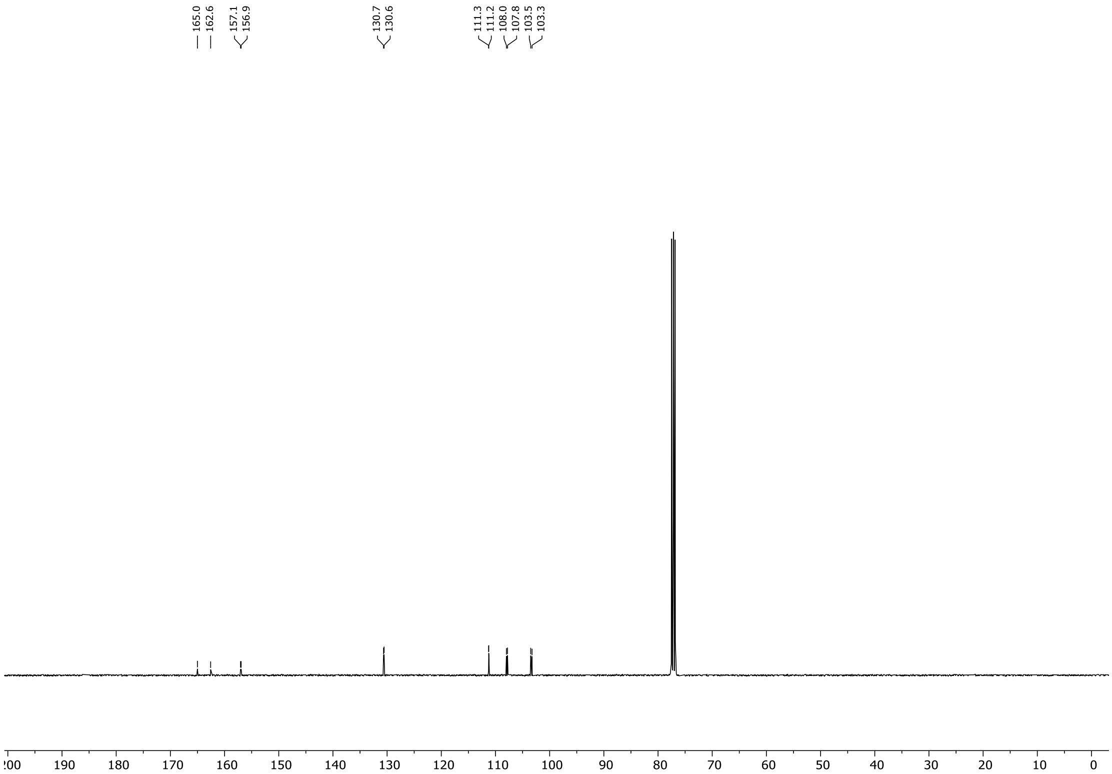
165.0
162.6
157.1
156.9
130.7
130.6
111.3
111.2
108.0
107.8
103.5
103.3
190 180 170 160 150 140 130 120 110 100 90 80 70 60 50 40 30 20 10 0

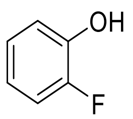
OH
F

2r

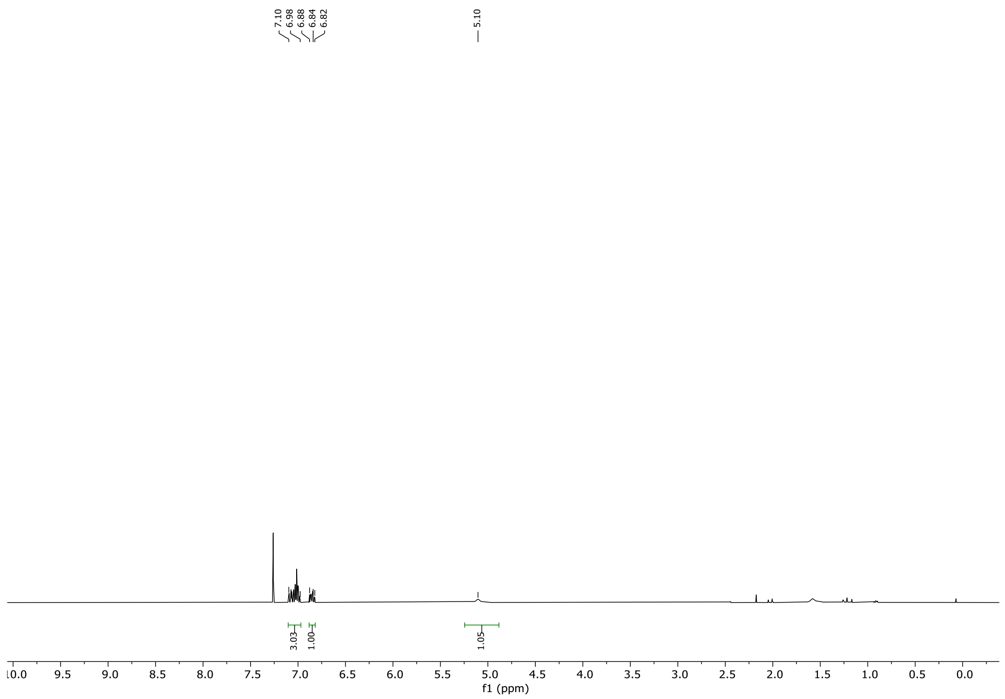
7.10
6.98
6.88
6.84
6.82
5.10
3.03
1.00
1.05
f1 (ppm)

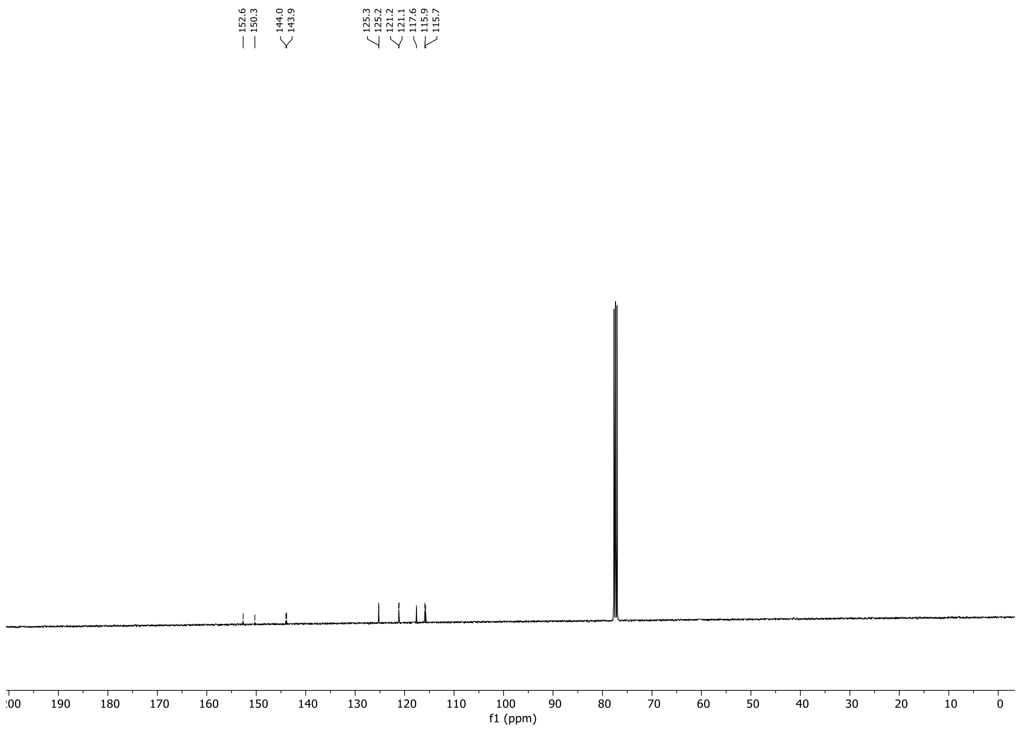
152.6
150.3
144.0
143.9
125.3
125.2
121.2
121.1
117.6
115.9
115.7
f1 (ppm)

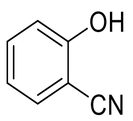
OH
CN

2s

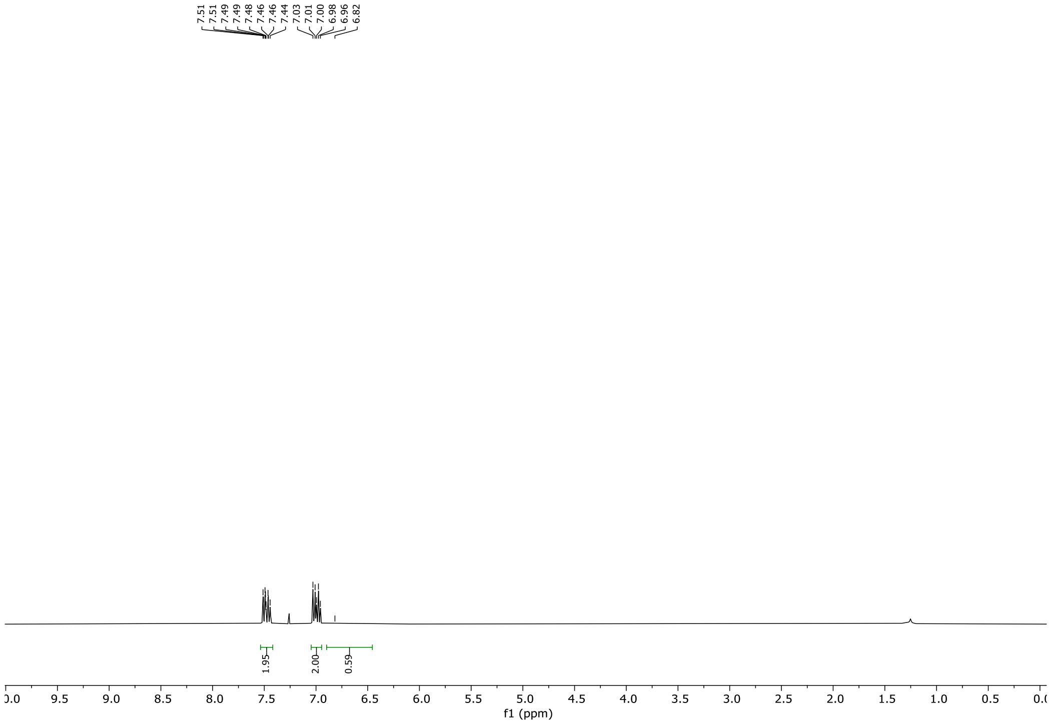
7.51
7.51
7.49
7.49
7.48
7.46
7.46
7.44
7.03
7.01
7.00
6.98
6.96
6.82
1.95
2.00
0.59
f1 (ppm)

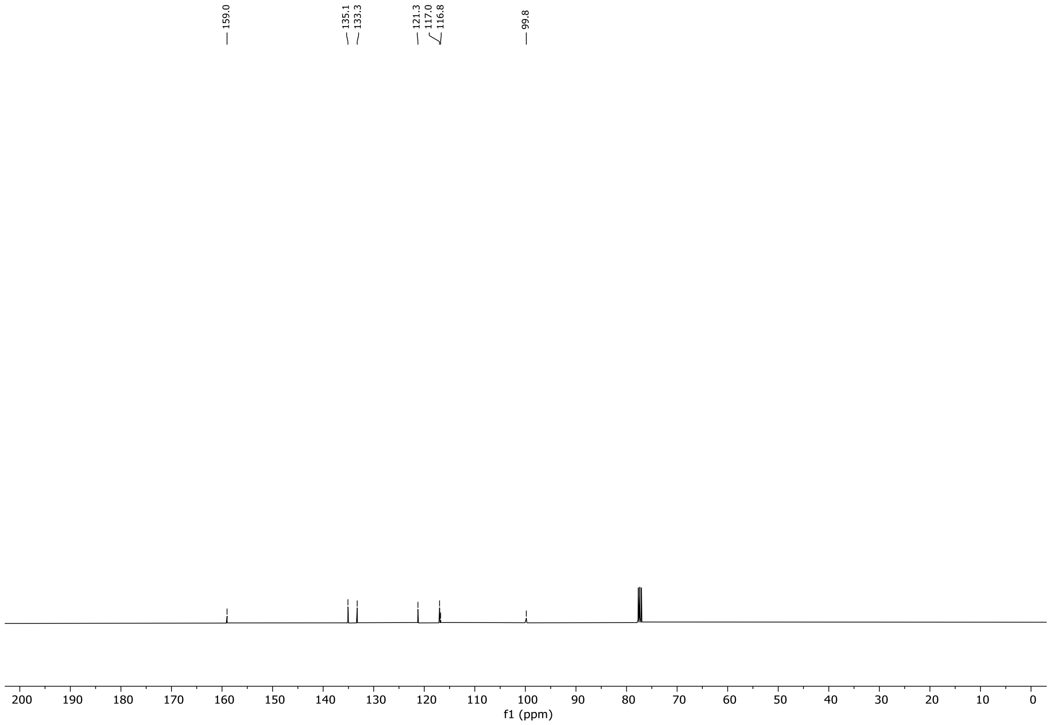
159.0
135.1
133.3
121.3
117.0
116.8
99.8
f1 (ppm)

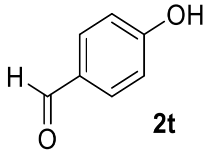
OH
H
O

2t

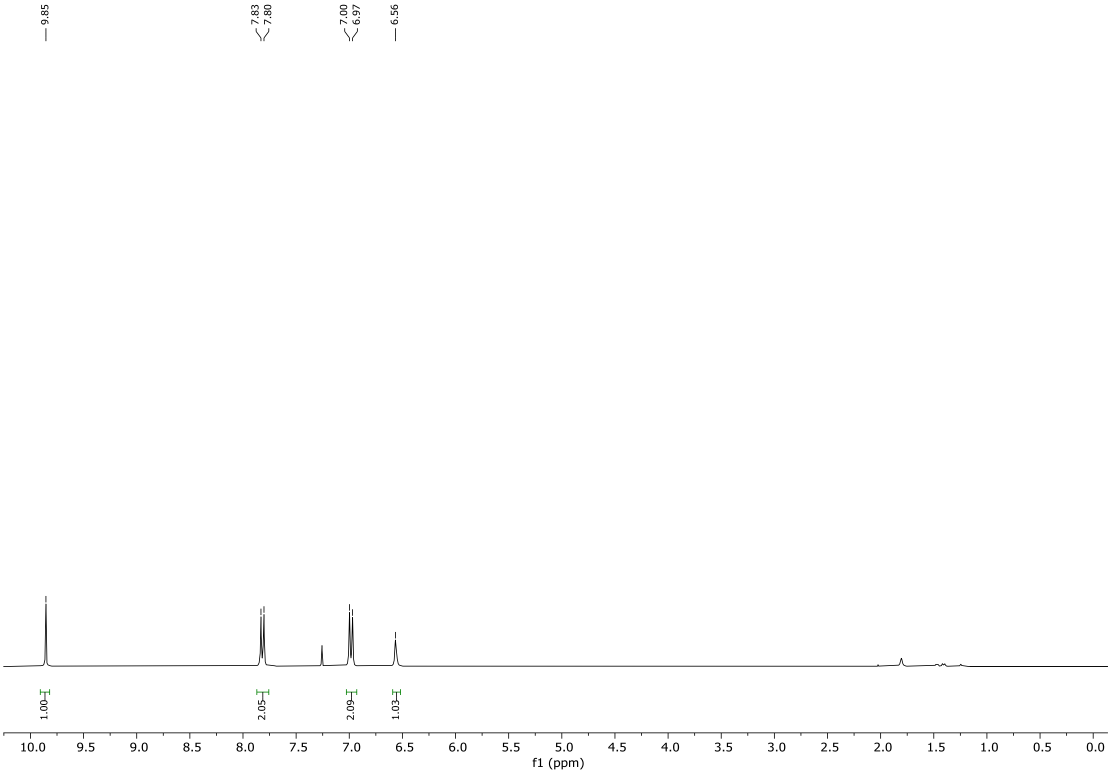
9.85
7.83
7.80
7.00
6.97
6.56
1.00
2.05
2.09
1.03
10.0 9.5 9.0 8.5 8.0 7.5 7.0 6.5 6.0 5.5 5.0 4.5 4.0 3.5 3.0 2.5 2.0 1.5 1.0 0.5 0.0
f1 (ppm)

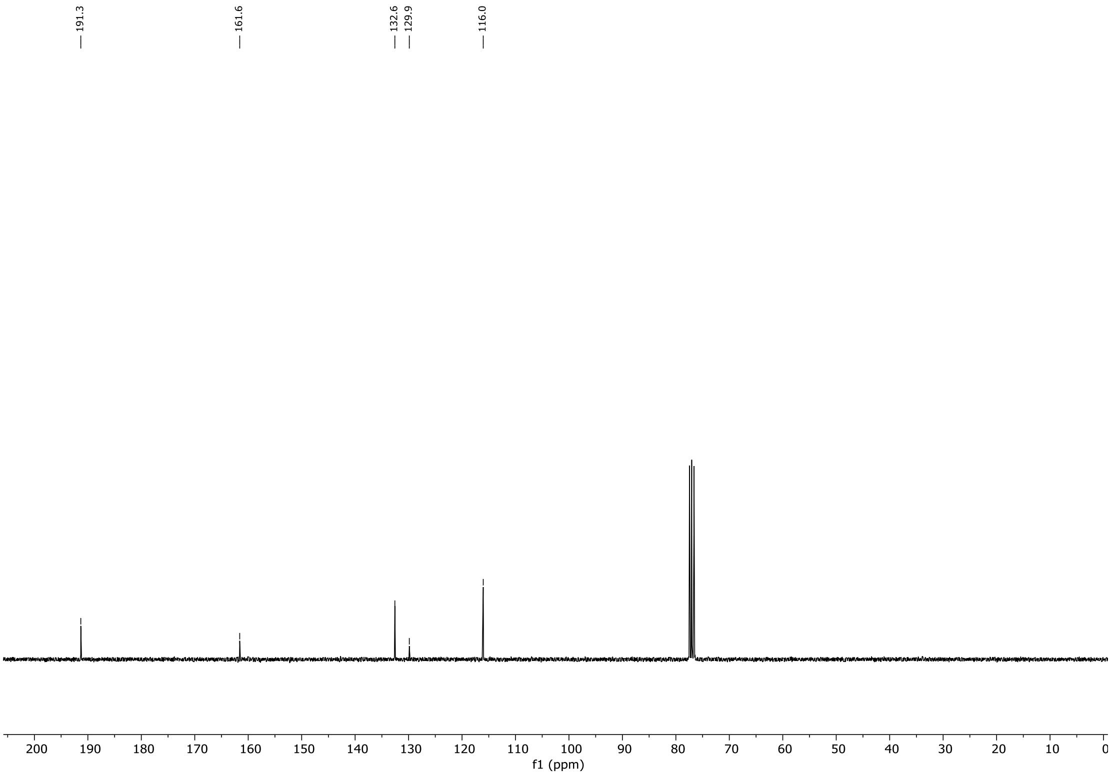
191.3
161.6
132.6
129.9
116.0
200 190 180 170 160 150 140 130 120 110 100 90 80 70 60 50 40 30 20 10 0
f1 (ppm)

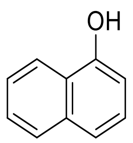

2u

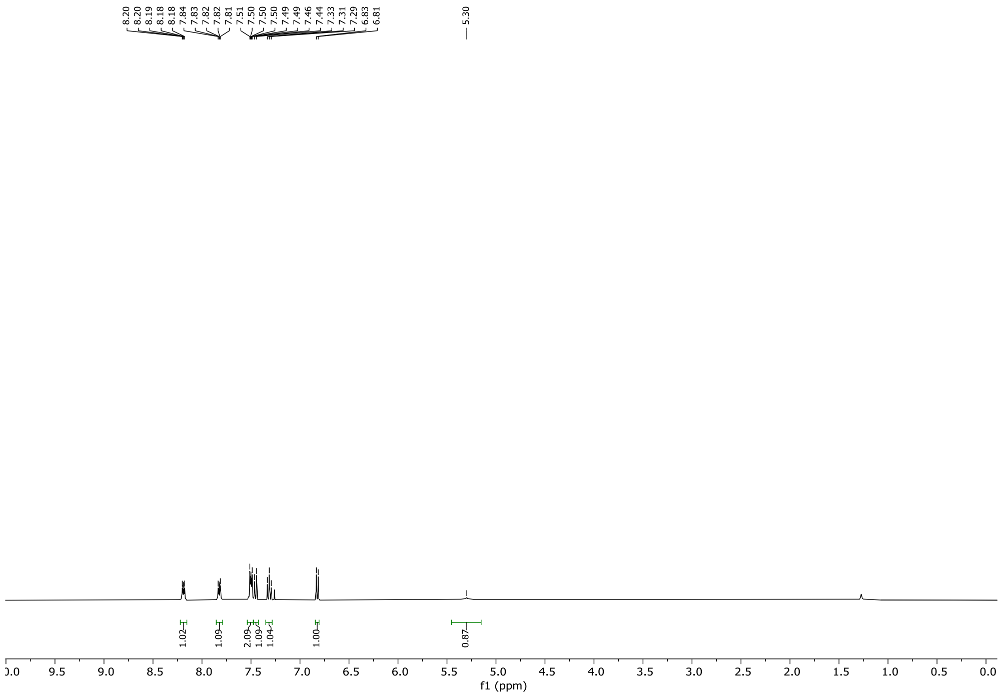

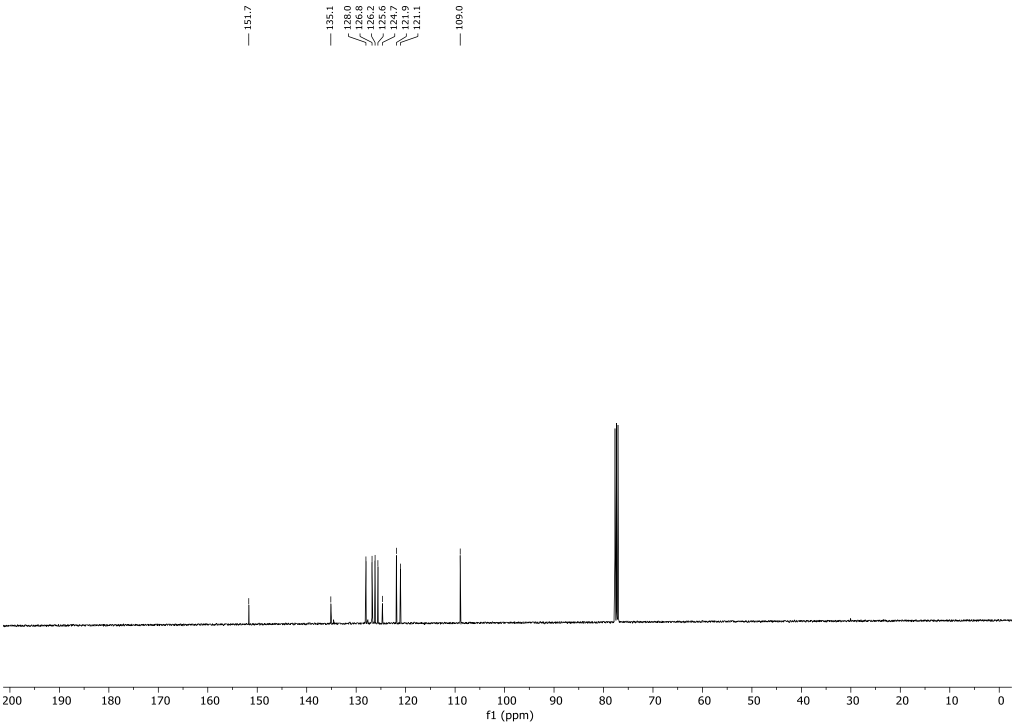

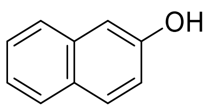
OH

2v

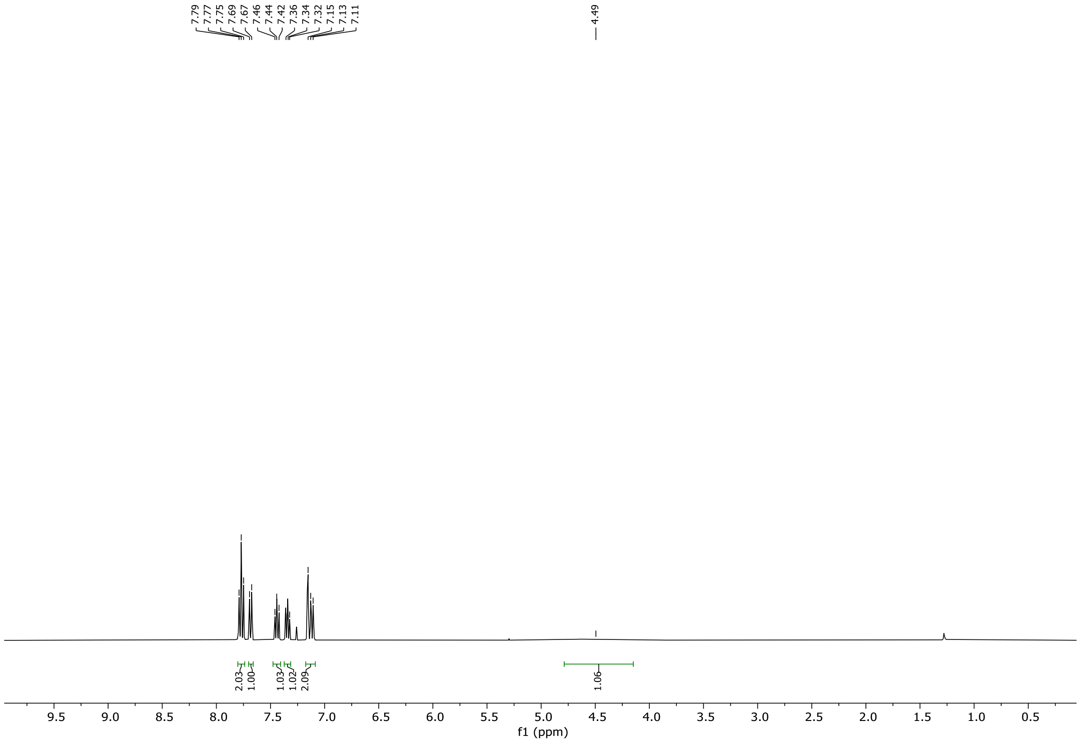
7.79
7.77
7.75
7.69
7.67
7.46
7.44
7.42
7.36
7.34
7.32
7.15
7.13
7.11
4.49
2.03
1.00
1.03
1.02
2.09
1.06
9.5
9.0
8.5
8.0
7.5
7.0
6.5
6.0
5.5
5.0
4.5
4.0
3.5
3.0
2.5
2.0
1.5
1.0
0.5
f1 (ppm)

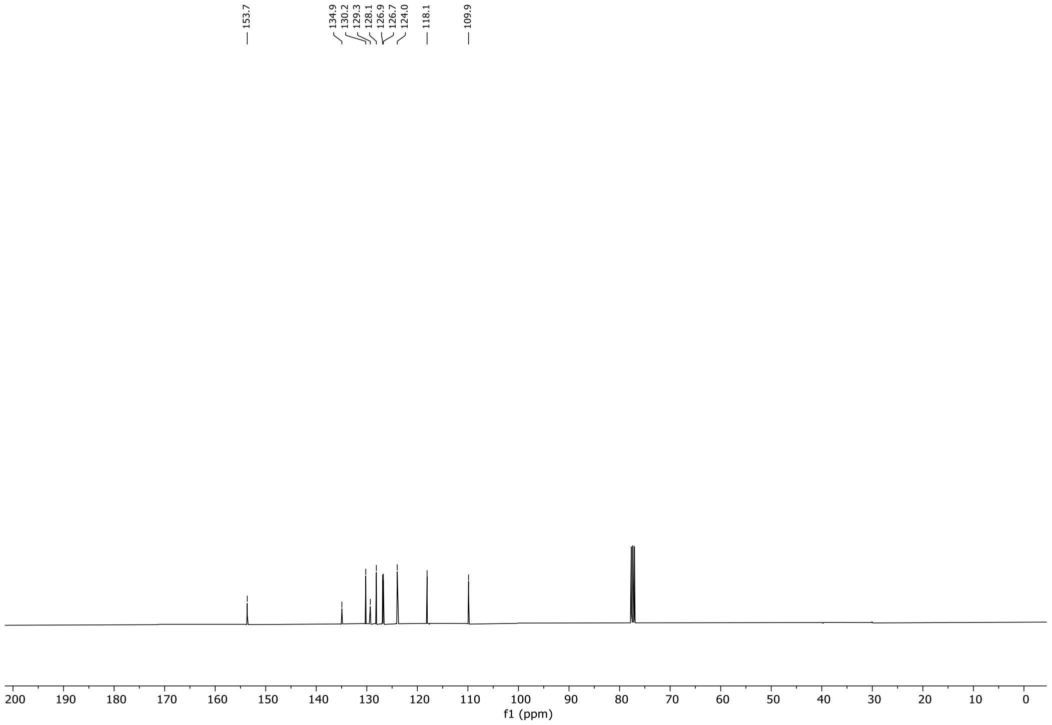
153.7
134.9
130.2
129.3
128.1
126.9
126.7
124.0
118.1
109.9
200
190
180
170
160
150
140
130
120
110
100
90
80
70
60
50
40
30
20
10
0
f1 (ppm)

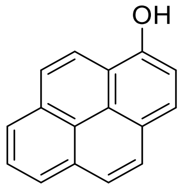
OH

2w

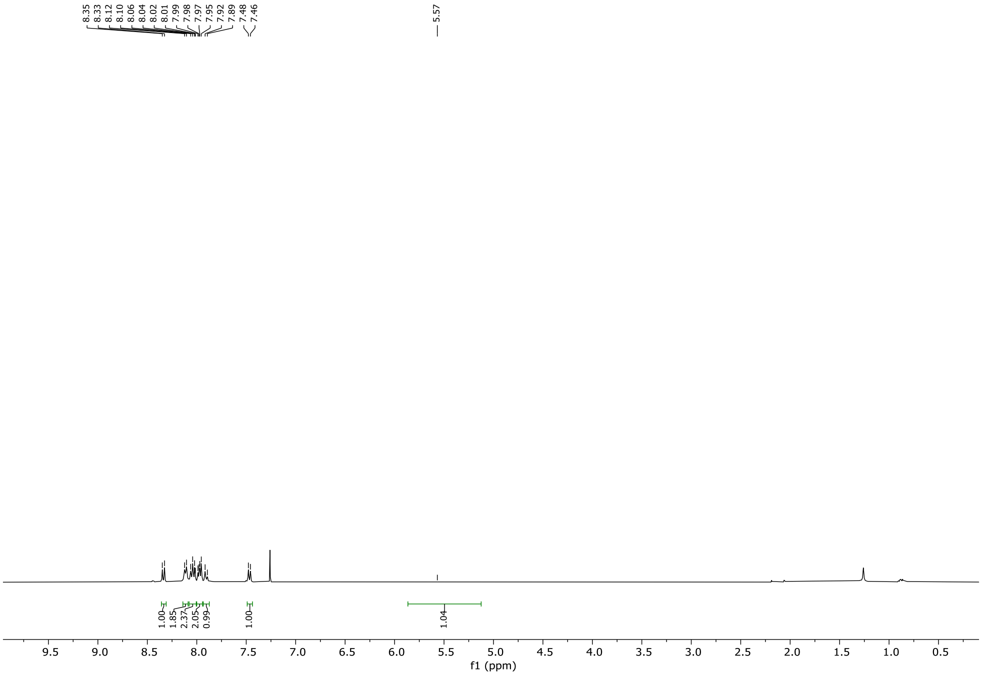
8.35
8.33
8.12
8.10
8.06
8.04
8.02
8.01
7.99
7.98
7.97
7.95
7.92
7.89
7.48
7.46
5.57
1.00
1.85
2.37
2.05
0.99
1.00
1.04
9.5 9.0 8.5 8.0 7.5 7.0 6.5 6.0 5.5 5.0 4.5 4.0 3.5 3.0 2.5 2.0 1.5 1.0 0.5
f1 (ppm)

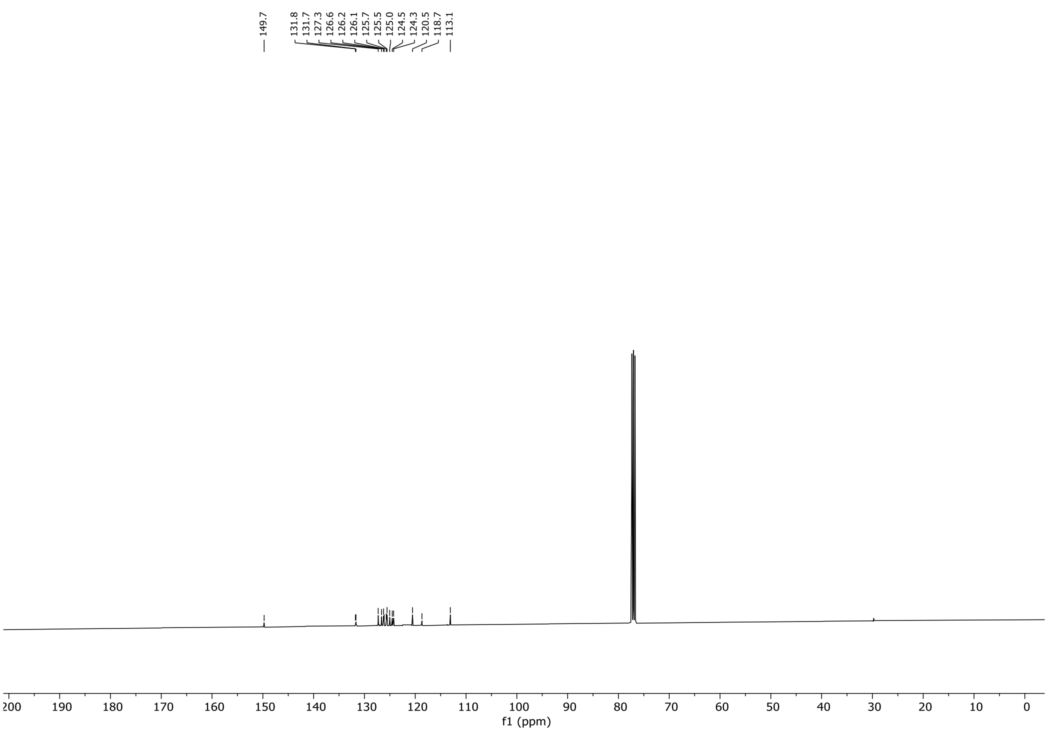
149.7
131.8
131.7
127.3
126.6
126.2
126.1
125.7
125.5
125.0
124.5
124.3
120.5
118.7
113.1
200 190 180 170 160 150 140 130 120 110 100 90 80 70 60 50 40 30 20 10 0
f1 (ppm)

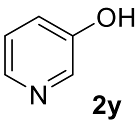
OH
N

2y

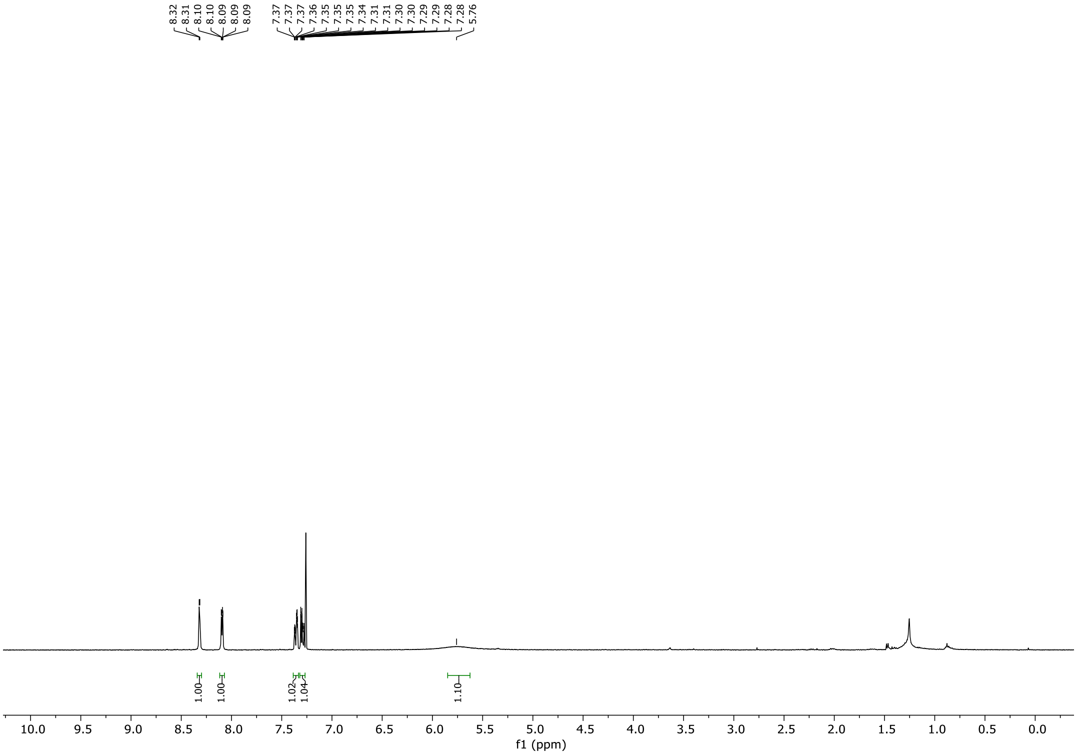
8.32
8.31
8.10
8.10
8.09
8.09
8.09
7.37
7.37
7.37
7.36
7.35
7.35
7.35
7.34
7.31
7.31
7.30
7.30
7.29
7.29
7.28
7.28
5.76
1.00
1.00
1.02
1.04
1.10
f1 (ppm)

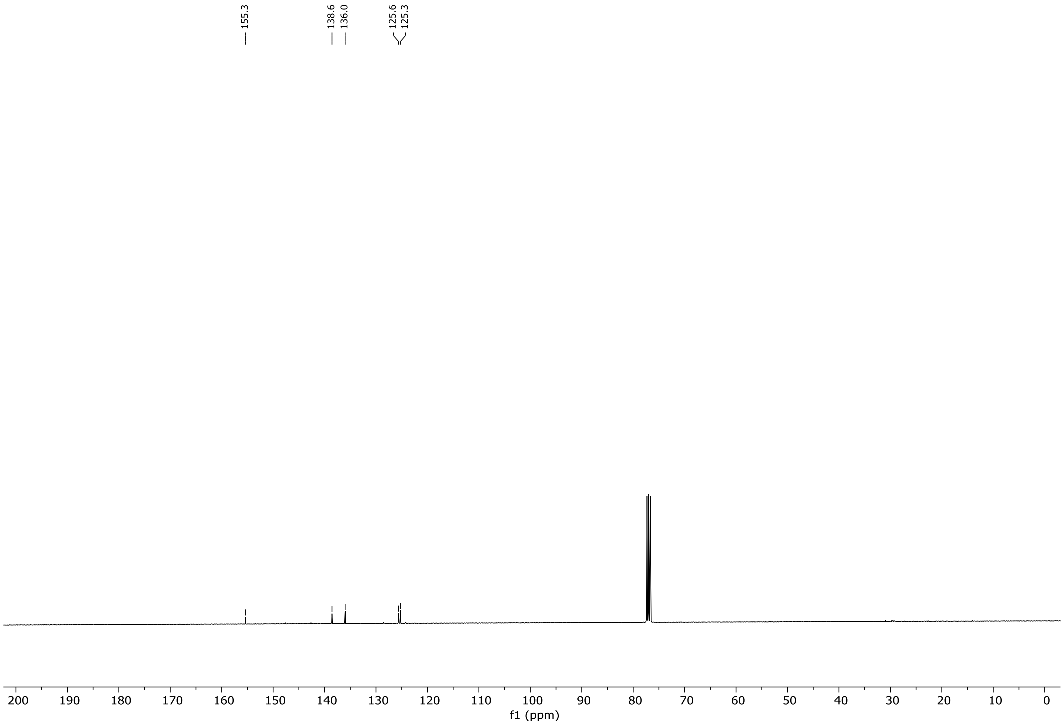
155.3
138.6
136.0
125.6
125.3
f1 (ppm)

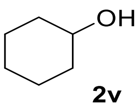
OH

2v

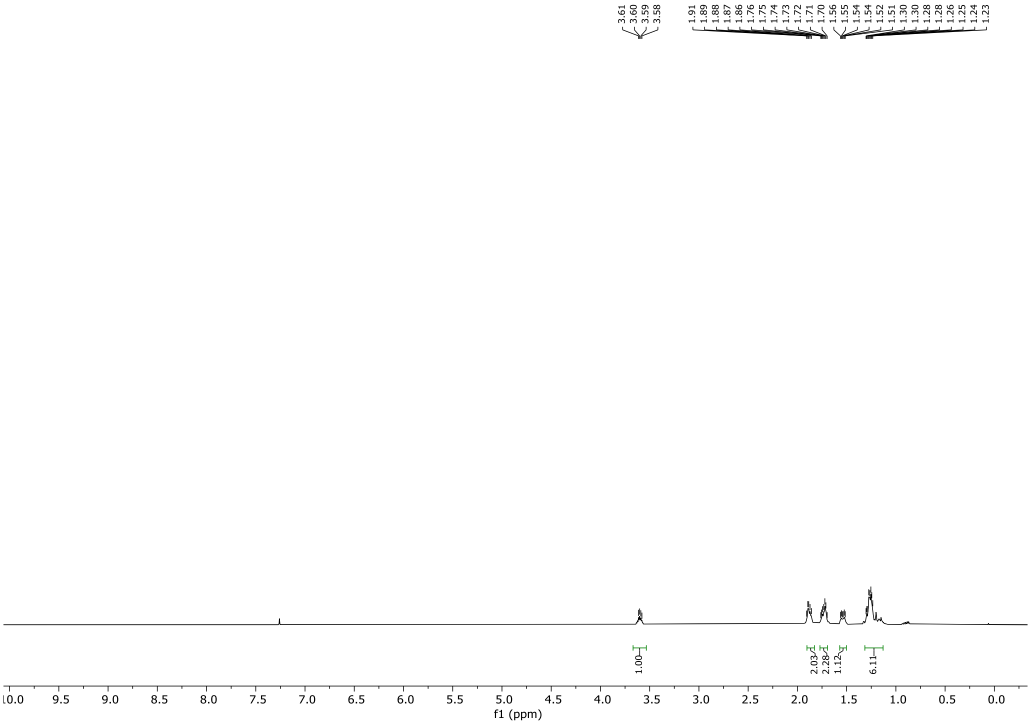
3.61
3.60
3.59
3.58
1.91
1.89
1.88
1.87
1.86
1.76
1.75
1.74
1.73
1.72
1.71
1.70
1.56
1.55
1.54
1.54
1.52
1.51
1.30
1.30
1.28
1.28
1.26
1.25
1.24
1.23
1.00
2.03
2.28
1.12
6.11
f1 (ppm)

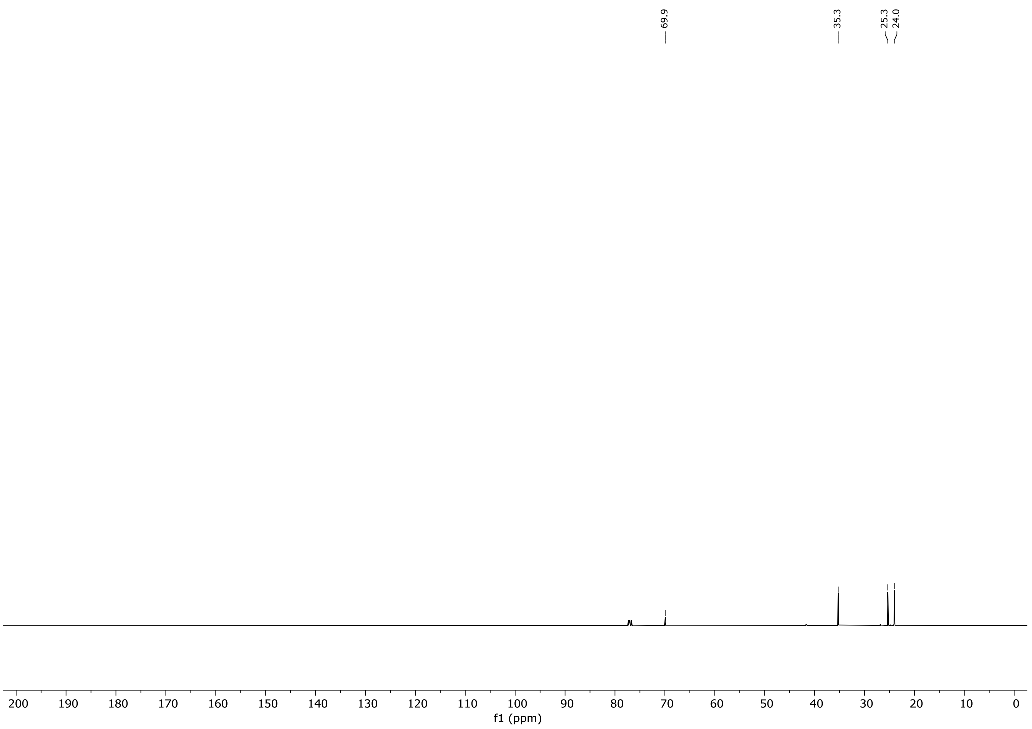
69.9
35.3
25.3
24.0
f1 (ppm)

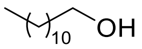
OH
10

2z

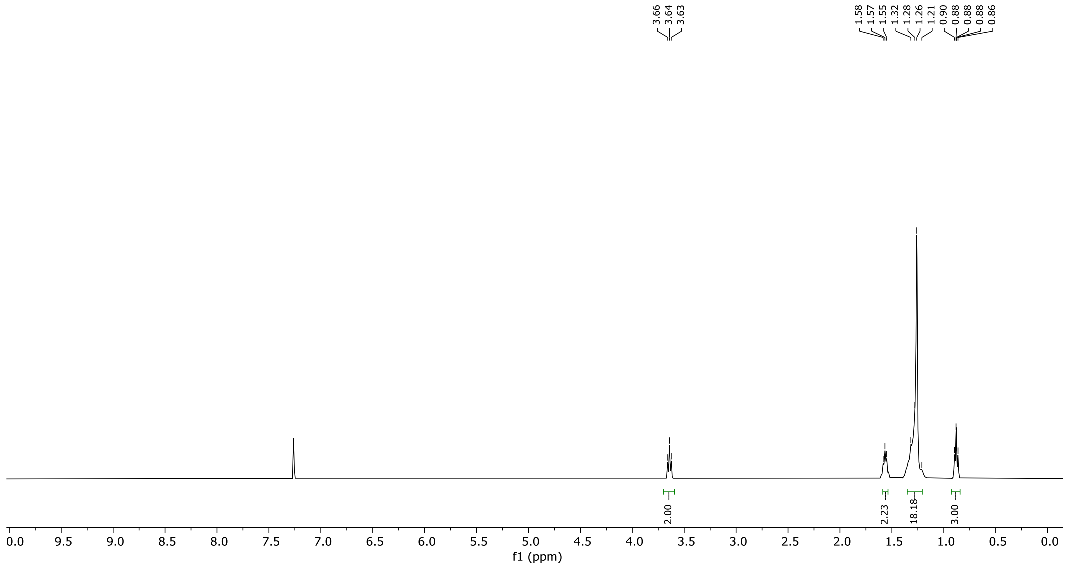
3.66
3.64
3.63
1.58
1.57
1.55
1.32
1.28
1.26
1.21
0.90
0.88
0.88
0.88
0.86
2.00
2.23
18.18
3.00
f1 (ppm)

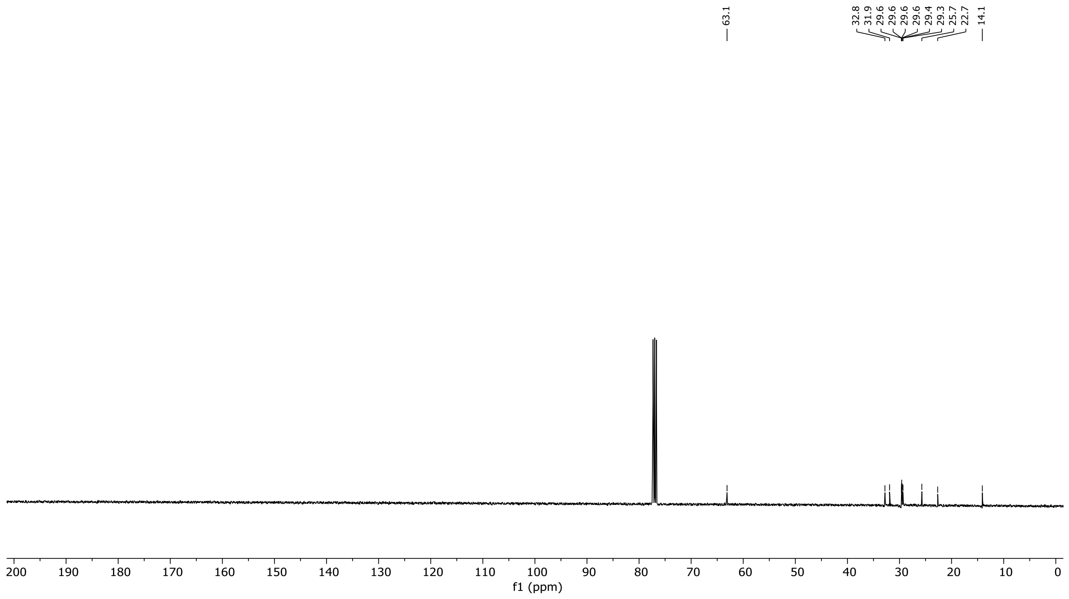
63.1
32.8
31.9
29.6
29.6
29.6
29.6
29.4
29.3
25.7
22.7
14.1
f1 (ppm)
